# Modelling the impact of increasing tuberculosis treatment coverage and addressing determinants of risk in men

**DOI:** 10.1038/s43856-026-01536-3

**Published:** 2026-03-27

**Authors:** Alexandra S. Richards, Mphatso D. Phiri, Jasper Nidoi, Jeremiah Chakaya, Peter MacPherson, Bruce J. Kirenga, John S. Bimba, Chukwuebuka Ugwu, Rhoda Pola, S. Bertel Squire, Katherine C. Horton

**Affiliations:** 1 Department of Infectious Disease Epidemiology, Faculty of Epidemiology and Public Health, London School of Hygiene and Tropical Medicine, London, UK; 2 TB Modelling Group, London School of Hygiene and Tropical Medicine, London, UK; 3 Malawi Liverpool Wellcome Research Programme, Blantyre, Malawi; 4 Clinical Sciences, Liverpool School of Tropical Medicine, Liverpool, United Kingdom of Great Britain and Northern Ireland; 5 Research and Innovation, Makerere University Lung Institute, Kampala, Uganda; 6 Department of Medicine, Dermatology and Therapeutics, Kenyatta University, Nairobi, Kenya; 7 Public Health and Research Unit, Respiratory Society of Kenya, Nairobi, Kenya; 8 School of Health & Wellbeing, University of Glasgow, Glasgow, UK; 9 Zankli Research Centre, Bingham University, Karu, Nasarawa Nigeria; 10 Faculty of Clinical Sciences & International Public Health, Liverpool School of Tropical Medicine, Liverpool, UK

**Keywords:** Tuberculosis, Epidemiology

## Abstract

**Background:**

Globally, the burden of tuberculosis falls more on men than women and children, and there are large gaps between men and women at all stages of exposure, disease incidence, and treatment. We examined the impact of addressing determinants of these gender gaps in Kenya, Malawi, Nigeria, and Uganda.

**Methods:**

We created a deterministic transmission model of tuberculosis, calibrated to country-specific data on prevalence, incidence, mortality, and notifications between 2010 and 2022. We examined the potential epidemiological impact of strategies to increase treatment coverage among men and decrease the effects of social and structural determinants that increase men’s risk of developing TB. We investigated the impact (overall and by age and sex) on projected incidence and mortality in 2035, and notification rates between 2025 and 2030.

**Results:**

Our modelling estimates that increasing treatment coverage among men could reduce incidence in 2035 between 2.4% [95% uncertainty interval (UI) 0.2-6.0%] in Malawi and 23.0% [UI 16.8-29.3%] in Nigeria. Reducing men’s excess risk of tuberculosis could similarly reduce incidence in 2035 between 9.8% [UI 7.5-12.6%] in Malawi and 30.1% [UI 24.1-40.5%] in Kenya. Impacts extend across the population with median estimates of country-level declines in incidence of between 0.9-17.8% and 1.4-22.2% in women and children, respectively, across the four countries.

**Conclusions:**

Strategies that prioritise increasing tuberculosis treatment coverage among men and mitigating men’s higher susceptibility to tuberculosis could reduce disease burden for men, women, and children. Such gender-responsive strategies are essential to ensure a person-centred tuberculosis response and accelerate global progress towards the EndTB targets

## Plain language summary

Globally, more men have tuberculosis than women or children, due to factors such as difficulty accessing care and increased exposure to risk factors such as high levels of alcohol consumption and tobacco smoking. We focussed on four countries, Kenya, Malawi, Nigeria, and Uganda, and built a mathematical model to understand the impact of increasing men’s access to diagnosis and treatment and the impact of reducing men’s exposure to risk factors. We found that strategies that focus on men not only reduce tuberculosis in men, but also across the entire population.

## Abstract (web summary)

Richards et al. use a compartmental transmission model of tuberculosis to understand the impact of strategies to improve men’s access to care and to reduce men’s excess risk of TB. In all four countries modelled, they find that these strategies could reduce incidence and mortality both in men and more widely across the population.

## Introduction

Tuberculosis (TB) is the leading infectious cause of death globally, with 10.8 million people developing TB disease and 1.25 million people dying from TB in 2023^1^. The greatest burden of TB falls on men (aged ≥15 years), who account for an estimated 55% of all cases globally, compared to 33% in women and 12% in children^1^. These sex differences exist through the course of the disease, with men having a greater exposure to *Mycobacterium tuberculosis (M.tb)*, a higher risk of infection, and a higher incidence of disease than women^2,3^. Men also experience longer durations of untreated disease due to social and structural barriers to healthcare, leading to higher prevalence to notification (P:N) ratios for men than women^1,4,5^. As such, men are estimated to make up two-thirds of the adults who developed TB but were unable to access diagnosis and treatment in 2023^1^.

People unable to access diagnosis and treatment represent a key failure in the TB response, as they remain outside the reach of lifesaving care and contribute to ongoing community transmission. Concerted efforts to reach these people are essential to end TB. Between 2015 and 2023, there were reductions in global incidence of 8.3% and mortality of 23%, but these gains fall well below the EndTB 2025 milestones of 50% and 75% reductions respectively^1^. The EndTB targets for 2035 go further, with an aim of a 90% reduction in incidence and 95% reduction in mortality, relative to levels in 2015; current trends are well below what is needed to achieve these targets^1^.

Tremendous progress in reducing the TB burden has been made in the World Health Organization (WHO) African region, with a 24% reduction in incidence since 2015^1^. However, this progress is driven primarily by a reduction in HIV-associated TB burden through the rapid expansion of HIV prevention and treatment during this time^1^. Whilst TB incidence in HIV-positive people has almost halved in the last eight years, the same is not true among HIV-negative people, where TB incidence has been increasing steadily since 2015 and is now 10% higher than it was in 2015^1^. As such, half of the WHO’s high TB burden countries are in the African region^1^.

Our work considers four countries with a high burden of TB and HIV from the African region: Kenya, Malawi, Nigeria, and Uganda^1,6^. Spread across east, south, and west Africa, each country presents a unique profile of the challenges faced to reduce the TB burden. The WHO considers Kenya, Nigeria, and Uganda to have a high TB burden; all four countries to have a high TB-HIV burden; and Nigeria to have a high drug-resistant TB burden^1^. Between 2012 and 2016, these countries each conducted a national TB prevalence survey that estimated the true burden of undiagnosed TB within the country^1,7^. These surveys also highlighted the gender disparities, with higher estimates for TB prevalence for men than women in each country^1,7^.

As in the rest of the world, these disparities in burden can partially be explained by gaps in access to treatment and diagnosis but may also be the result of other social and structural determinants of health that impact men more than women. At an individual level, there are likely biological differences that make men more susceptible to TB than women^8^. Interacting with these baseline differences are the social aspects, such as higher levels of alcohol consumption and tobacco smoking among men than women, and institutional aspects, such as occupational risks, limited social protection, higher rates of incarceration, and primary healthcare systems oriented towards maternal and child health^1,9–11^. Men also mix socially more with other men than with women, meaning that, alongside being more at risk of TB, they also have greater exposure to TB^12–15^.

Reducing the burden of disease in men by addressing these disparities could accelerate progress towards the EndTB targets. To investigate the potential epidemiological impact of strategies to improve men’s access to TB care and to reduce men’s excess risk of TB, we use mathematical modelling to examine the impact of each of these strategies on incidence and mortality at both a population level and among sub-populations, specifically men, women, and children. We measure the impact of the strategies in reductions of incidence and mortality and the change in notifications over time compared to a scenario where the strategies had not been implemented. Our modelling work highlights the population-level benefits of reaching men, a high-burden group under-represented in TB prevention and care in Kenya, Malawi, Nigeria, and Uganda.

## Methods

### Model

**Fig. 1:**
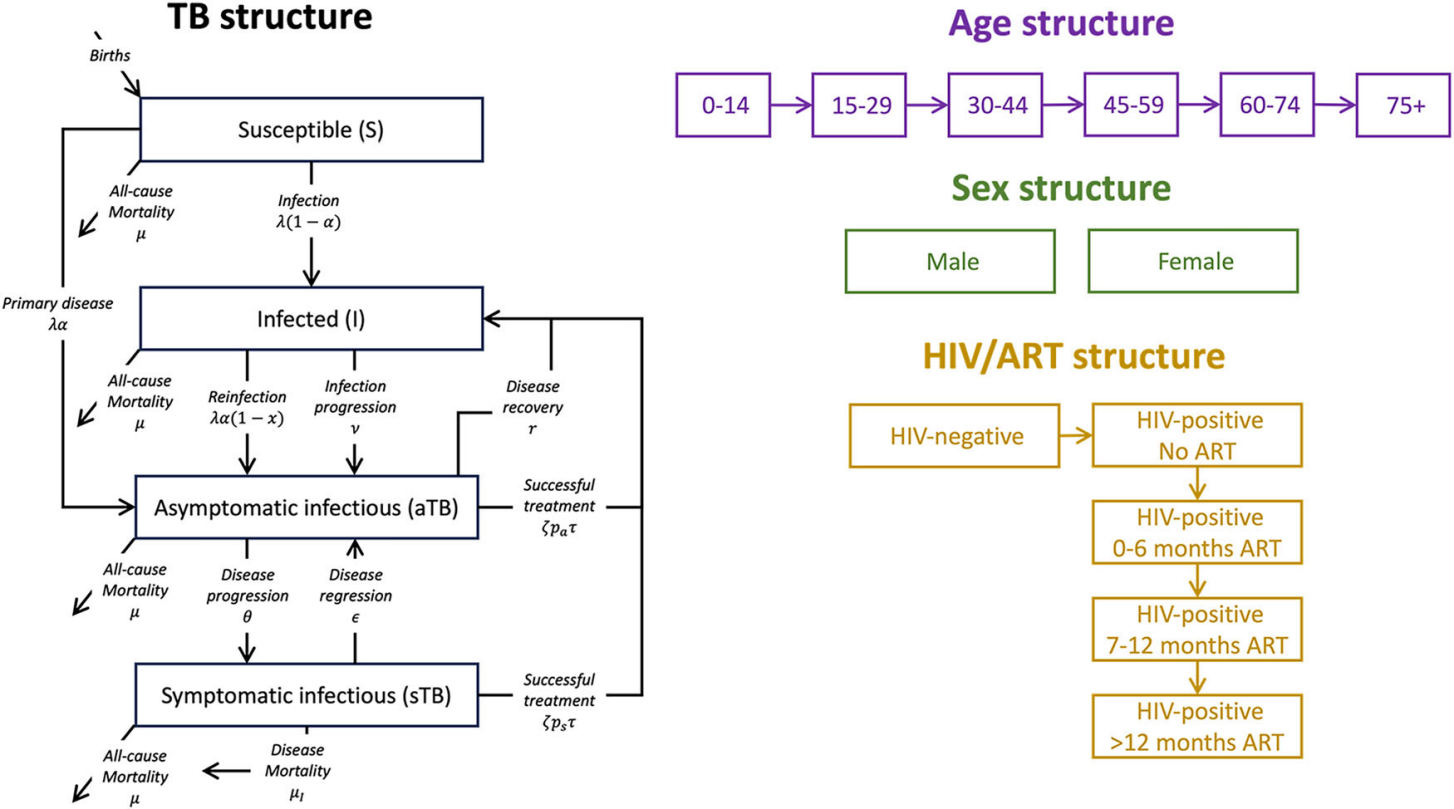
Model structure showing the TB transitions (left), the demographic transitions (top right), the sex strata (middle right) and the HIV/ART transitions (bottom right)

We developed a compartmental model of TB disease and *M.tb* transmission, building on previously published models (Supplementary Information, Section 1)^16,17^. As shown in Fig. 1, the model comprises four TB states: susceptible (uninfected), infection, asymptomatic infectious TB and symptomatic infectious TB (where infectious implies bacteriological positivity)^18^. The states of infection and disease are stratified by TB treatment history, and all compartments are further stratified by sex (male/female), age group (0–14, 15–29, …, 60–74, 75+), and HIV and antiretroviral treatment (ART) status (HIV negative, HIV positive no treatment, HIV positive 0-6 months treatment, HIV positive 7-12 months treatment, HIV positive >12 months treatment).

### Data

For each country, the population in each age group, HIV incidence and prevalence, and ART uptake incidence and prevalence were informed by time-series estimates, reported annually by sex in five-year age groups, from the DemProj and AIM models of the Spectrum suite^19–21^. At each time point, values from our model were cross-checked with AIM outputs, and the incidence of HIV and ART initiation required to match the estimate was calculated and applied^16^. This ensured that the sex differences in HIV burden and ART uptake in each country were accurately incorporated into the model. The model was also informed by annual country-level data on vaccination coverage (with the Bacillus Calmette-Guérin [BCG] vaccine), TB treatment coverage (defined as the proportion of estimated incidence that is notified each year), and TB treatment success (the proportion of people who successfully complete a course of TB treatment) for both HIV-negative and HIV-positive populations, as reported by the WHO^22–25^.

Sex- and age-assortative mixing patterns are reflected in the model with a social contact matrix between men (males ≥ 15 years), women (females ≥15 years), and children (both sexes age <15 years). Country-specific sex- and age-assortative matrices were only available for Kenya and Malawi; we used a summary contact matrix from all available surveys conducted within Africa for Nigeria and Uganda^12–15^.

Prior ranges for all parameters were informed by literature, where available. TB natural history parameters were informed by studies that have collated historical data to best represent the progression of *Mtb* infection and TB disease^26–29^. The impact of age, HIV, and ART on TB natural history parameters were based on previous estimates^16,17^. We assumed there were differential effects on treatment coverage from the population average depending on age (children less likely to access care for TB), sex (men less likely, and women more likely to access care for TB), symptom status (asymptomatic disease less likely to prompt care-seeking), and ART status (more likely to receive TB testing as part of ART care). To account for the increased frequency of TB disease among men compared to women, we incorporated a parameter to represent the quantifiable, but not specifically assigned, excess risk. A full list of parameters and prior ranges is available in the Supplementary Information, Section 3.

### Calibration

For each country, we calibrated the model to country-specific epidemiological targets of TB prevalence, incidence, mortality, and case notifications for the overall population and, where possible, for age, sex, and HIV strata^6,22,30–32^. TB prevalence targets, both overall and by sex, were informed by the national TB prevalence surveys^30,33–36^. TB incidence, overall and for men, women, and TB-HIV status, and TB mortality, overall and by TB-HIV status, were based on WHO estimates^22^. Notification rates, overall and by sex, age, and HIV status, were based on surveillance data reported by each country^31,32^.

The model was calibrated using a history matching and emulation algorithm (*hmer*) in R^37–39^. This algorithm is an iterative process of testing the outputs of a selection of parameter sets that span the available space against the calibration targets, calculating the implausibility of those parameter sets, and discarding the most implausible^37^. This results in a set of parameters that fit the calibration targets and span the available parameter space^37^. For each country, we ran the algorithm through up to 10 rounds of the iterative process (stopped earlier if there was no improvement between already successful rounds), and then used the emulators from the best performing round to generate at least 1000 parameter sets for each country^37^. The resulting parameter sets were randomly sampled to select 1000 parameter sets to be used for further analysis. Figures showing the progress of each round of calibration are included in the supplementary information for each country (Supplementary Figs. 6, 9, 12, and 15).

Results for the calibration are presented with plots of the distributions of the 1,000 parameter sets compared to the priors and median values of those parameters with 95% credible intervals (CrIs), along with plots illustrating how the calibrated model fitted the epidemiological targets over time (Supplementary Figs. 7, 10, 13, 16, Supplementary Tables 23–26, and Supplementary Figs. 8, 11, 14, 17). Results are presented with the median and 95% uncertainty intervals (UIs) of the outputs from these 1,000 parameter sets. We also calculated partial rank correlation coefficients to understand the dependence of the outcomes on each of the parameter values (Supplementary Information, Section 7.1).

### Gender-responsive strategies

We considered three main strategies: improving TB treatment coverage in men to match the levels achieved in women by 2030, reducing men’s excess risk of TB, and a combination of the two. Each strategy was implemented over a period of five years from 2025 to 2030 with an S-shaped uptake curve (further details can be found in the Supplementary Information, Section 6). Improving treatment coverage in men was modelled as increasing the calibrated parameter for treatment coverage specific to men to match the calibrated parameter for women’s treatment coverage. The reduction of excess risk was modelled as reducing the calibrated parameter representing men’s risk by 50% of the excess above 1^8^. The third strategy combined these two strategies over the same five-year period. Supplementary analyses of excess risk reductions by 25% and 75% have been included in the Supplementary Information, Section 7.5).

### Analysis

We evaluated each strategy by comparing the projected TB incidence, prevalence, mortality, and notifications (Supplementary Information, Section 7.2) in 2035 and 2050 to a business-as-usual (BAU) scenario. The BAU scenario assumed that the TB treatment coverage and other time-dependent parameters, such as treatment success and BCG coverage, would continue at similar levels to 2023, but that no new strategies for TB or HIV would be introduced. We also evaluated the same outcomes against a BAU scenario where TB treatment coverage continued to improve over time (Supplementary Information, Section 7.6). Unless otherwise specified, results are presented for the entire population. Additional supplementary analyses were included for halving the treatment coverage difference between men and women (Supplementary Information, Section 7.4) and varying the levels of men’s risk reduction to 25% and 75% (Supplementary Information, Section 7.5).

## Results

### Calibration

Each country was successfully calibrated to its respective set of epidemiological target ranges, with posterior parameter estimates and model estimates compared to calibration targets available in the Supplementary Information (Section 5.2.1). All four countries showed decreasing prevalence, incidence, and mortality over the period 2010-2022. In both Kenya and Malawi, the P:N ratio decreased slightly over time for both men and women. Nigeria and Uganda had relatively stable P:N ratios until each country began increasing active case finding initiatives in 2020 and 2022, respectively, when the P:N ratio decreased for men and women^40,41^. Despite these improvements in treatment coverage, in all four countries there was a small, but continued, increase in the male-to-female prevalence ratio from 2010 until 2022. Plots showing the calibrated model trends are shown in the supplementary information (Supplementary Figs. 8, 11, 14, 17). Partial rank correlation coefficients suggested that women’s treatment coverage and men’s excess risk parameters were most highly correlated with model outcomes (Supplementary Information, Section 7.1).

### Impact on burden

**Fig. 2:**
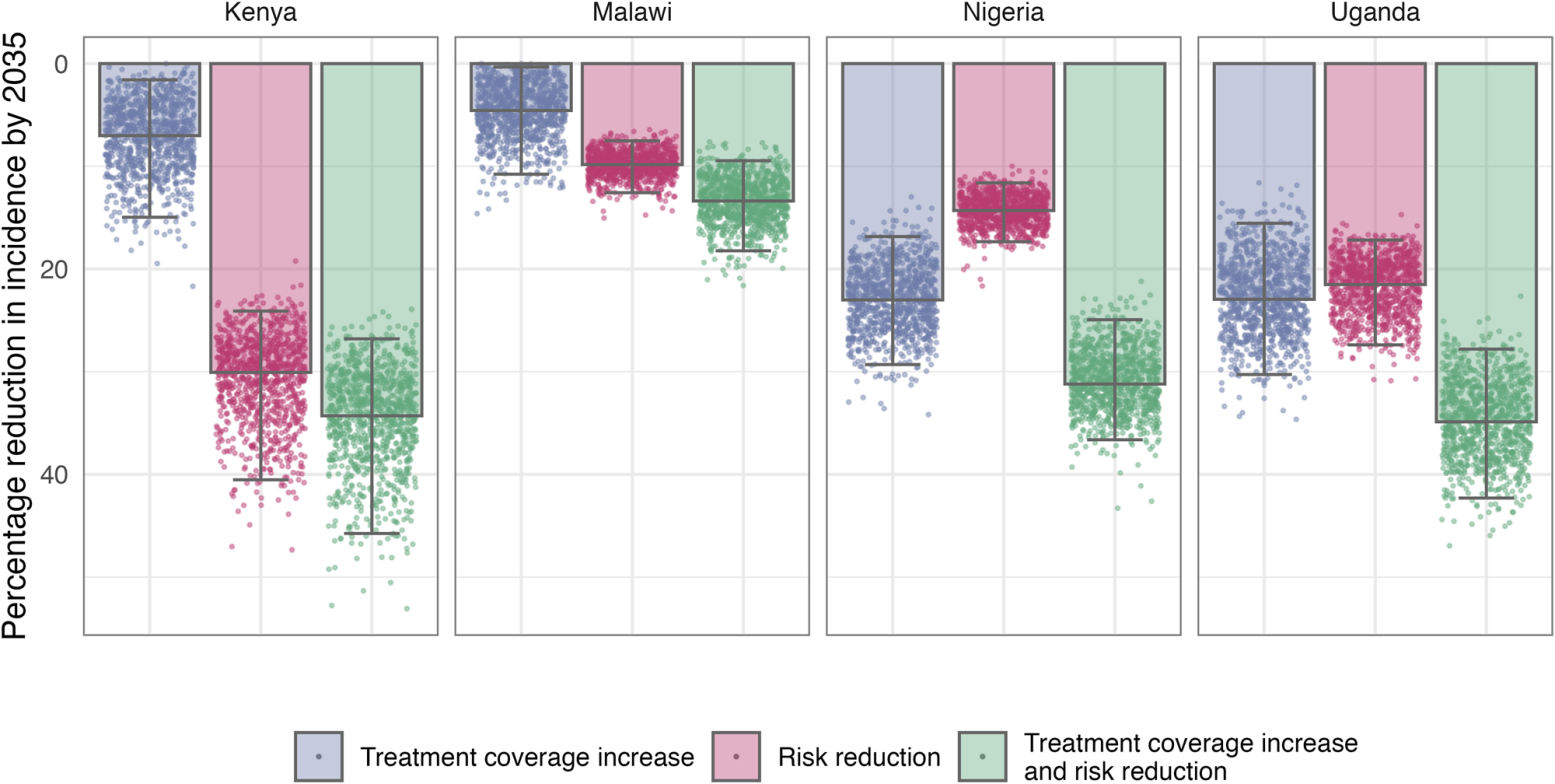
Percentage reductions in 2035 TB incidence, relative to a business-as-usual scenario, across the three strategies to address gender disparities in TB - increasing treatment coverage in men, reducing men’s excess risk of TB by 50%, and the combination of these two. Coloured bars show the median estimate and error bars show the 95% uncertainty interval, each dot represents the results of a single model run (1000 total).

Increasing men’s treatment coverage was projected to have the greatest impact on disease burden in Nigeria and Uganda, with a 23.0% [UI 16.8–29.3%] and 23.0% [UI 15.5–30.3%] reduction, respectively, in 2035 incidence relative to BAU. This strategy was expected to avert 72,433 [UI 51,127–95,821] TB deaths in Nigeria and 5216 [UI 3614–7481] TB deaths in Uganda between 2025 and 2035. The relative reduction in TB incidence was lower for this scenario in Kenya, with an 7.0% [UI 1.6–15.0%] reduction in 2035 incidence relative to BAU, and Malawi, where 2035 incidence was 4.6% [UI 0.3–10.8%] lower than BAU (Fig. 2). In Kenya, increasing men’s treatment coverage was estimated to prevent 8071 [UI 1750–18,405] TB deaths, while in Malawi, 1199 [UI 91–3400] TB deaths were averted between 2025 and 2035.

Reducing men’s excess risk of TB leads to similar relative reductions in 2035 incidence relative to BAU, at 30.1% [UI 24.1–40.5%] and 21.5% [UI 17.2–27.4%] in Kenya and Uganda, a respectively, with 27,749 [UI 19,980–40,356] and 3331 [UI 2362–4692] TB deaths averted, respectively. The impact of this strategy was projected to be more limited in Nigeria and Malawi, with 14.3% [UI 11.6–17.3%] and 9.8% [UI 7.5–12.6%] respective reductions in 2035 incidence relative to BAU. Between 2025 and 2035, reducing men’s excess TB risk was projected to avert 1670 [UI 1105–2601] TB deaths in Malawi and 29,480 [UI 22,038–38,740] TB deaths in Nigeria.

Combining the two strategies was projected to lead to greater reductions in TB burden. As with the individual strategies, the greatest relative reduction from the combined strategy was in Uganda, where we projected a 34.9% [UI 27.8–42.3%] reduction in 2035 incidence relative to BAU and 7081 [UI 5228–9634] TB deaths averted between 2025 and 2035. Reductions in 2035 incidence were similar in Nigeria and Kenya, 31.2% [UI 24.9–36.6%] and 34.3% [UI 26.8–45.8%] respectively. Due to differences in population size, the number of TB deaths averted from this strategy was much larger in Nigeria (89,635 [UI 66,340–113,820]) than in Kenya (33,310 [UI 23,644–49,129]). With a 13.4% [UI 9.5–18.2%] reduction in 2035 incidence relative to BAU, the impact was more limited in Malawi, but we project 2745 [UI 1537-4961] TB deaths would be averted here between 2025 and 2035.

### Impact on men, women, and children

**Fig. 3:**
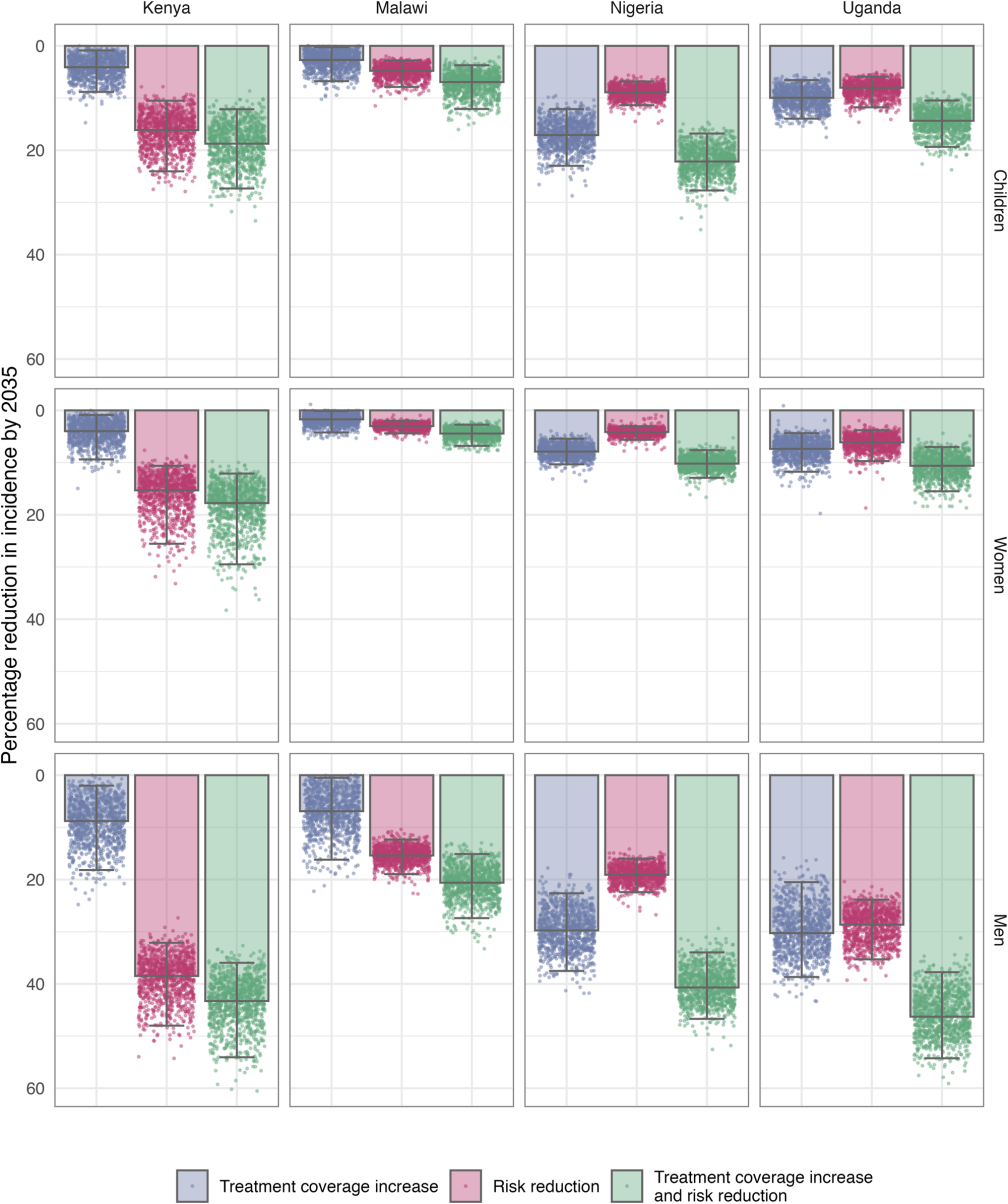
Percentage reductions in 2035 TB incidence in men, women, and children, relative to a business-as-usual scenario, across the three strategies to address gender disparities in TB - increasing treatment coverage in men, reducing men’s excess risk of TB by 50%, and the combination of these two. Coloured bars show the median estimate and error bars show the 95% uncertainty interval; each dot represents the results of a single model run (1000 total).

The impact of these strategies varied among men, women, and children (Fig. 3). As the strategies targeted men, as expected, the greatest impact was seen among men. The median estimate for the percentage reduction in incidence for men ranged between 18.1% (Malawi) and 46.3% (Uganda) in 2035 compared to BAU, translating to a percentage increase from the population level of between 26% (Malawi) and 54% (Kenya). In women, the combined strategy reduced incidence by between 3.8% (Malawi) and 17.8% (Kenya), equivalent to a percentage decrease from the population level of between 48% (Kenya) and 70% (Uganda). Children were estimated to have a median incidence reduction of between 6.0% (Malawi) and 22.2% (Nigeria), equating to a percentage decrease from the population level of between 29% (Nigeria) and 60% (Uganda). Despite the lower impact in women and children, all strategies provide a significant reduction in incidence.

### Impact on notifications

As expected, increasing treatment coverage in men initially increased notifications across all countries with peak increases of 3.6% [UI 0.7–7.8%] and 3.2% [UI 0.2–8.1%] in Kenya and Malawi, respectively, in 2029. These percentage increases translated to an additional 2103 [UI 432–4702] notifications in Kenya and an additional 413 [UI 32–1123] notifications in Malawi in 2029 compared to BAU. The peak increase in notifications in Nigeria and Uganda occurred a year earlier, but resulted in much greater increases at 17.4% [UI 12.0–23.2%] and 11.5% [UI 7.4–16.8%] respectively, equating to an additional 28,326 [UI 19,942–37,028] notifications in Nigeria and an additional 3201 [UI 2090–4369] notifications in Uganda in 2028 compared to BAU.

Conversely, reducing men’s risk of TB did not increase notifications at any point, with reductions observed from 2027 onwards. Combining these two interventions resulted in marginal increases in notifications in Kenya and Malawi (0.8% [UI −0.8–3.0%] and 1.3% [UI −1.2–5.9%] respectively), a year earlier than with increasing treatment coverage alone. Nigeria and Uganda still experienced a substantial increase in notifications (14.1% [UI 9.1–20.3%] and 7.8% [UI 3.8–12.0%] respectively), with an additional 23,608 [UI 15,305–33,166] notifications in Nigeria and an extra 2,146 [UI 1,064–3,157] notifications in Uganda, in 2028.

**Fig. 4:**
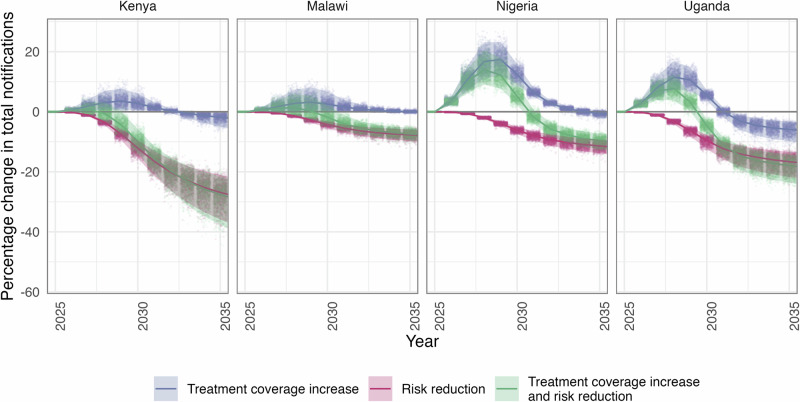
The percentage change in notifications from a baseline of no intervention for the five years before, the five years during, and the five years after the implementation of each strategy (increasing treatment coverage in men, reducing men’s excess risk of TB by 50%, and the combination of these two). The central lines show the median estimate for the relative percentage reduction, and the shaded regions show the 95% uncertainty interval; each dot represents the results of a single model run (1000 total).

While short-term impacts over the first two years of implementation showed increased notifications, longer-term impacts showed a reduction in notifications across strategies by 2035. Figure 4 shows that reductions in overall notifications by increasing treatment coverage among men had a substantial impact in Uganda (5.9% [UI 3.3–9.8%] reduction) but resulted in marginal changes in Kenya, Malawi, and Nigeria. Changes in notifications from both the risk reduction strategy and the combined strategy were similar, with greater reductions in Kenya and Uganda (26.9% [UI 21.1–35.9%] and 16.6% [UI 13.4–21.7%] respectively) than in Malawi and Nigeria (7.7% [UI 5.9–9.8%] and 11.4% [UI 9.3–13.8%] respectively).

### Sensitivity analyses

Halving the improvements in treatment coverage reduced the impact on incidence by slightly less than half, but had less overall impact when combined with a 50% reduction in excess risk (Supplementary Fig. 26). Sensitivity analyses were also conducted for a 25% and 75% reduction in excess risk. As expected, reducing excess risk by only 25% reduced the impact of risk reduction and the combined strategy, while reducing excess risk by 75% increased effects (Supplementary Figs. 27 and 28). Notable differences were that with a 25% reduction in excess risk, the risk reduction strategy alone became substantially less effective than the treatment coverage strategy in Nigeria, and with a 75% reduction in excess risk, the risk reduction strategy alone became substantially more effective than the treatment coverage strategy in Kenya.

Percentage reductions in each measure were similar across all four countries.

While the main results focus on the period through 2035, we also modelled predictions of impact through 2050 (Supplementary Figs. 24 and 25) and, with time, each intervention had a greater impact on incidence, likely reflecting non-linear effects. Reduction in excess risk in men yielded slightly greater improvements over time than improving treatment coverage in men, and there was more of an improvement of impact for women and children than there was for men. For each intervention, the number of notifications also continued to reduce over time, reflecting progressive reductions in disease burden, with the greatest improvement in the combined strategy in all four countries.

The sensitivity analysis considering future scenarios with increasing baseline treatment coverage found that proportional impacts on incidence and mortality are the same as the main analysis (Supplementary Information, Section 7.6).

## Discussion

Our results show that addressing gender disparities in access to TB care and determinants of risk may result in declines in TB incidence of between 13.4% [UI 9.5–18.2%] in Malawi and 34.9% [UI 27.8–42.3%] in Uganda. These reductions are greater than the reductions that have been achieved globally in the last 10 years and so have important implications for TB care, prevention, and programming^1^. In settings with large gaps in treatment coverage between men and women, such as Nigeria and Uganda, closing this gap may be particularly effective at reducing TB burden in men and subsequently throughout the whole population. Where gendered gaps persist beyond treatment coverage, such as in Kenya, strategies that focus on reducing men’s risk of TB may be more effective at reducing TB burden. Whilst these strategies focus solely on men, the impact extends across the population, with reductions in incidence and mortality in women and children.

These findings call for greater attention to gender in TB responses. An emerging body of evidence highlights strategies to improve men’s access to TB diagnosis and treatment in healthcare facilities, occupational, and community settings. In Uganda, recent work as part of the IGNITE study found that stigma and societal expectations of masculinity, paired with clinic hours being only available during work hours, prevented men from accessing care^42^. The proposed interventions included strategic clinic opening times, either outside of normal working hours, or on market days, and education to improve TB awareness among men^42^. In Nigeria, in the DESTINE study, a Delphi process found that targeted awareness campaigns and screening in men’s congregate settings were the preferred strategy to increase men’s access to care^43^. Both the proposals of clinics on market days and outside of working hours were found to be less preferred in this Nigerian setting^43^. Occupational screening has yielded promising results in Kenya, with twice as many men as women initiated on treatment, community involvement and adaptability of processes are required for success across workplaces^44^. Targeting male-dominated industries, such as the fishing industry, and prisons, where 95% of incarcerated individuals are male, was trialled in Uganda and resulted in increased notifications across all groups^45–47^. Other studies have looked at opening male-specific clinics in rural Kenya and suggested that improved labour regulations and payment during sick leave in Malawi could help increase men’s participation in healthcare^48,49^.

Our findings also highlight a need to address gendered dimensions of social and structural TB determinants. While the distribution of TB determinants varies across different countries, and each country’s capacity to implement strategies that reduce these risks will differ, addressing these determinants is key to reducing disease burden and disparities^50^. Men have a higher prevalence of key health-related TB risk factors, including undernutrition, tobacco smoking, and alcohol consumption^1,9^. Men are also more likely to face occupational hazards (e.g., in the mining industry) or experience incarceration, further increasing their risk of TB exposure and disease^10,11^. It is likely that biological sex characteristics also contribute, a complete reduction of excess risk was not considered in this work^8^. The relative contribution of different social and structural determinants varies across countries; as such, national responses should be informed by local data. While action to address the root causes of these risks may lie outside the scope of a national TB programme, multi-sectoral efforts to address these risks could be expected to produce health and societal benefits that extend beyond TB^51^.

The strategies highlighted here are gender-specific or gender-sensitive. However, many of the barriers that men face in accessing TB care and the drivers of social and structural determinants that increase men’s TB-associated risks are grounded in cultural gender norms and expectations. Cultural expectations and ideas of masculinity have previously been reported to prevent men from accessing care for TB^5,52–55^. These include a cultural expectation that men are “tough”, “resilient”, and “independent”, meaning that they are slow to acknowledge symptoms, and may even deliberately delay treatment start in order to remain in work or even to prove their strength^5,52–55^. Addressing gender disparities in TB risks and care requires an understanding of cultural gender norms, which may differ within and between countries and may change with time. An ambitious agenda would look beyond gender-specific and gender-sensitive strategies and aim for gender-transformative approaches to address these underlying norms and expectations^55^. Gender-transformative strategies for TB care are currently limited^55,56^. Evidence of gender-transformative approaches within other health areas, including HIV and gender-based violence, suggests that these strategies may lead to changes in gendered attitudes^57^. Men who engaged in such interventions reported reflections on the costs of masculinity, and some behaviour change and risk reduction strategies were reported^57^.

Our analysis omitted some impacts of these interventions that cannot be fully modelled. Firstly, there is potential that any strategy that increases men’s access to care or decreases their excess risk may also impact women and children. This could come in the form of increased awareness and reduced stigma, improving utilisation of TB services, or through, for example, decreased exposure to second-hand tobacco smoke that would reduce risk. While we cannot quantify this additional effect, it would likely increase reductions in burden estimated here, both overall and among women and children. Secondly, increasing men’s access to TB care may offer opportunities for earlier diagnosis and treatment of other conditions, such as hypertension and diabetes, and addressing social and structural determinants may also reduce risk for other infectious and chronic conditions^58,59^. These additional impacts are beyond the scope of this work, but may be useful to consider.

We did not explicitly model the impact of the COVID-19 pandemic and related non-pharmaceutical interventions that may have affected risks of transmission and reduced treatment coverage; rather we have calibrated our model to estimates before and after the pandemic^60^. In our BAU scenario, we recognise other ongoing work to improve treatment coverage and have assumed that treatment coverage will continue to increase for all four countries included in this work^61^. We do not expect the proportional reductions shown in our modelling to change without gender-responsive approaches in each country. Our results do not incorporate within-country heterogeneity in disease burden, so any strategies introduced to higher burden areas, or areas with greater sex disparities, using local knowledge could result in greater impacts on burden than have been presented here^62^. Country-specific social contact matrices were only available for Kenya and Malawi, so the combined contact matrix used for Nigeria and Uganda may not reflect the true distributions of contacts by age and sex. The model calibration reflects trajectories of disease that have been observed over a 15-year period, and despite reflecting a potential optimistic trajectory, at the higher end of target ranges in 2010 and lower ends of the ranges in 2020, the proportional reductions in incidence and mortality are similar regardless of future treatment coverage, and so the results likely provide a reasonable estimate of the impact of the strategies that have been implemented. Calibration targets include model estimates for incidence and mortality. In the absence of empirical data, these estimates are the best available to understand the disease burden in each country. Our model structure only reflects infectious (bacteriologically positive) disease, which does not capture extrapulmonary disease and much of the disease seen in children. Whilst this will not make a significant difference within our main results, greater reductions in infectious disease will lead to lower transmission, resulting in lower rates of disease in children.

Our modelling work highlights the population-level benefits of reaching men, a high-burden group under-represented in TB prevention and care in Kenya, Malawi, Nigeria, and Uganda. These findings likely have implications for other settings given the widespread nature of gender disparities in TB burden and care. Our findings suggest that implementation of strategies in line with those described here could reduce TB morbidity and mortality across the population, accelerating progress towards the EndTB milestones.

## Supplementary information

Supplementary Information

## Data Availability

Data frames of all model outputs for this study are available at the GitHub repository https://github.com/alexandrasrichards/LIGHT_modelling within the Intervention results folders^63^. Data frames for outputs reported in this paper are available at the same GitHub repository within the Outputs folder^63^.

## Code availability

All model development, calibration, and analyses were performed using R 4.5.0^39^. Calibration used the hmer package (version 1.6.0), data analysis and visualisation used ggplot (3.5.2) and other packages within the tidyverse (version 2.0.0)^37,64,65^. The code used can be found in the GitHub repository https://github.com/alexandrasrichards/LIGHT_modelling^63^.
