# Supplementary material for: Modelling the impact of increasing tuberculosis treatment coverage and addressing determinants of risk in men
Source: Commun Med (Lond). 2026 Mar 27;6:293. doi: 10.1038/s43856-026-01536-3 (PMC13194917; doi:10.1038/s43856-026-01536-3)
Supplement: Supplementary file 1 — Supplementary Information [file 43856_2026_1536_MOESM1_ESM.pdf]

Supplementary Information

Table of Contents

1 Model Structure 4
1.1 Tuberculosis model 4
1.2 Demographic model 6
1.3 HIV model 6
2 Model equations 7
3 Model parameters 9
3.1 Force of infection 9
3.2 Progression to disease 11
3.2.1 Rapid progression to disease 11
3.2.2 Progression from infection 14
3.2.3 Reinfection 15
3.3 Progression through disease 16
3.4 Regression through disease 16
3.5 Self-cure 17
3.6 Care cascade 17
3.6.1 Access to care 17
3.6.2 CAST in Uganda 19
3.7 Treatment completion 20
3.8 Mortality 21
4 Model implementation 23
5 Calibration 24
5.1 Epidemiological calibration targets 24
5.1.1 Data sources 24
5.1.2 Calculation notes 25
5.2 Calibration process 28
5.2.1 Successful calibrations 29
6 Implementation of interventions 46
6.1 Adjusting access to care 46
6.2 Reducing excess risk 47
7 Supplementary results 49
7.1 Partial Rank Correlation Coefficients 49
7.2 Impact of interventions on mortality and prevalence compared to incidence 51
7.3 Impact out to 2050 52
7.3.1 Incidence 52

7.3.2 Notifications 53
7.4 Impact of only halving the gap in access to care between men and women .. 53
7.5 Impact of interventions for varying levels of risk reduction 54
7.5.1 Incidence 54
7.5.2 Notifications 56
7.6 Increasing access to care 58
7.6.1 Incidence overall 58
7.6.2 Incidence Men/Women/Children 59
7.6.3 Notifications overall 60
8 References 61

1 Model Structure

1.1 Tuberculosis model

We developed a sex-stratified dynamic compartmental model of *Mtb* transmission and infectious tuberculosis (TB) disease, adapted from previously published models.[1–3] The core TB model has four states: susceptible, infection, asymptomatic bacteriologically confirmed disease, and symptomatic bacteriologically confirmed disease, as shown in Supplementary Figure 1. Unlike in other variants of the model, disease states are split by symptoms rather than smear status. [2–4]

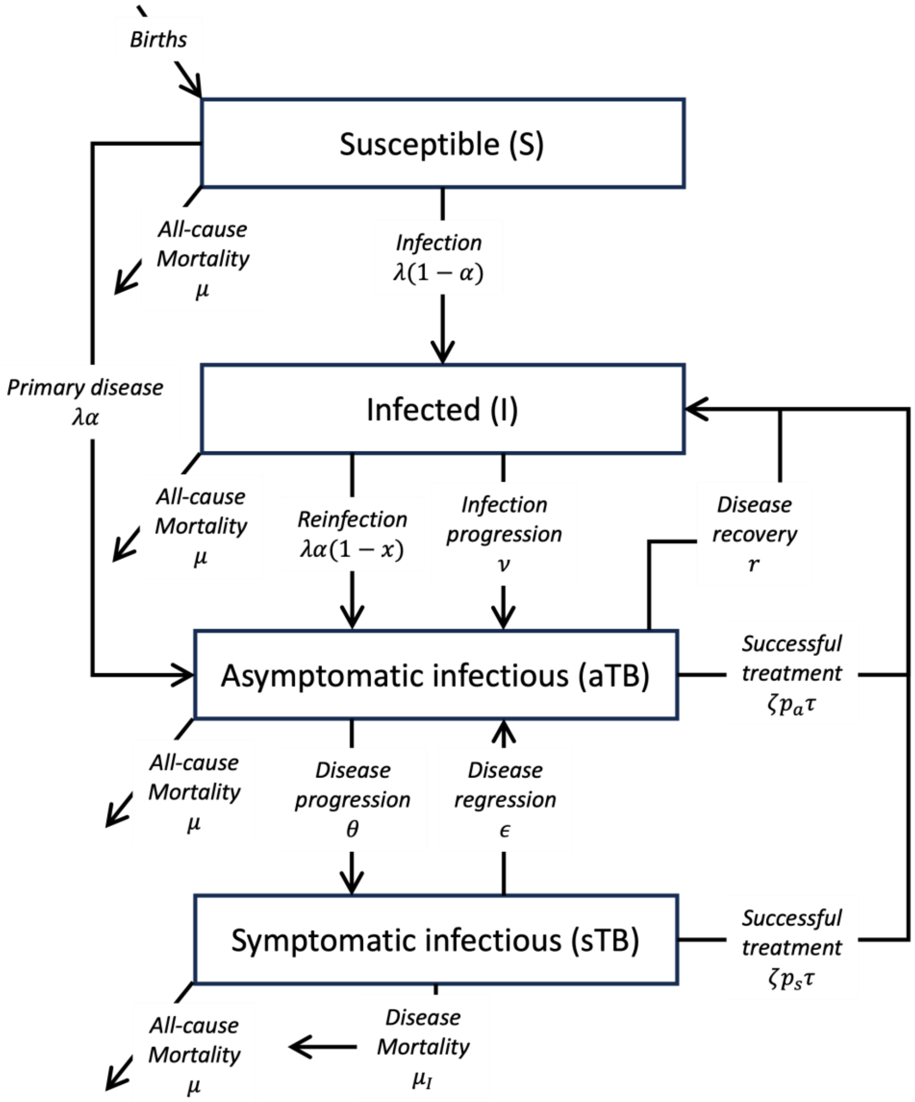

Supplementary Figure 1: The underlying TB model structure

Individuals enter the susceptible compartment (S) at birth and are at risk of all-cause mortality throughout the model.

Susceptible individuals (S) have a force of infection, λ. A proportion, α, of individuals progress directly to asymptomatic infectious disease, and the rest, $(1-\alpha)$, remain infected with *Mtb*. Infected individuals haven an annual rate of progression, ν, along

with a rate of progression through reinfection, at a rate $\lambda a(1-x)$, where x is the protection against progression to disease upon reinfection, provided by previous infections.

Individuals with asymptomatic infectious disease progress to symptomatic infectious disease at a rate θ, and they naturally clear disease at a rate r. Individuals with symptomatic infectious disease regress to asymptomatic infectious disease at a rate ϵ and die from disease at a rate μ_I.

Individuals who recover from TB disease return to the infected compartment, where they are at risk of reinfection and natural progression as described above. Natural recovery is only possible from asymptomatic disease, as described above, but recovery of disease through successful treatment is possible from both disease stages.

The infection and disease compartments (I, aTB, and sTB) are stratified by the treatment history, with treatment naive indicated by a subscript N and previously treated indicated by a subscript P. Individuals move from the treatment naive stratum to the previously treated stratum after any treatment, whether successful or unsuccessful, as shown in Supplementary Figure 2 and detailed in previous work. [1,5]

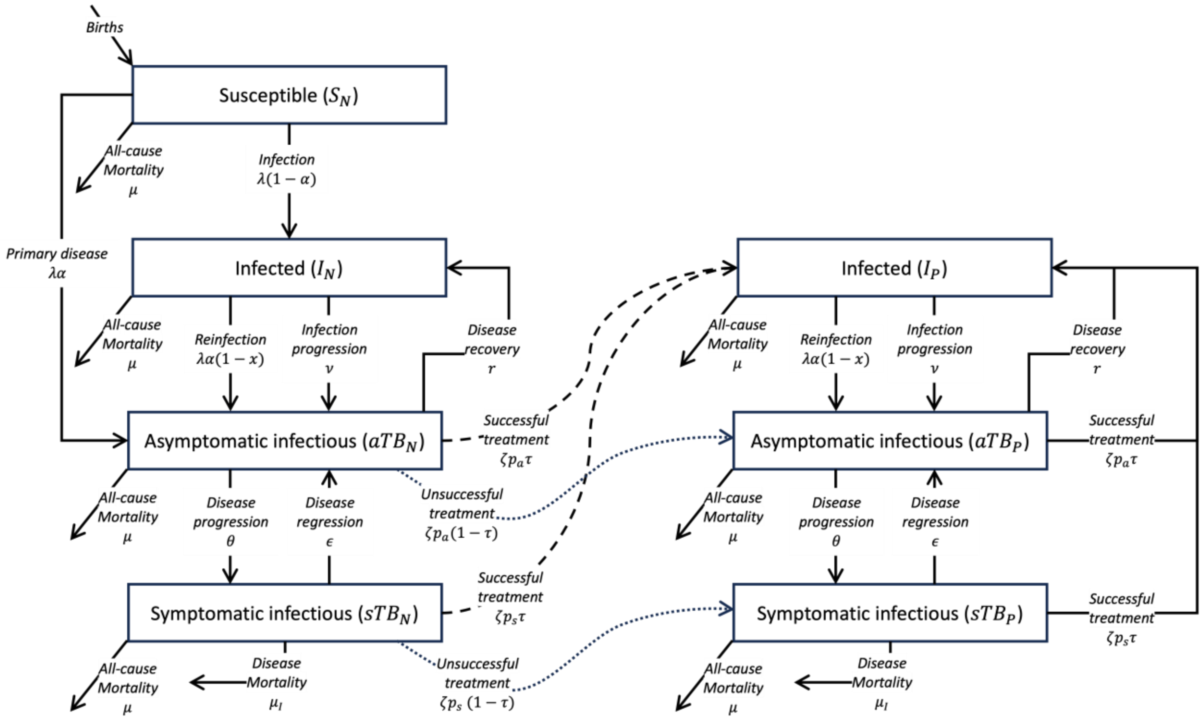

Supplementary Figure 2: The full TB treatment model structure

The full model is stratified by sex (male and female), age in fifteen-year age groups (0 - 14, 15 - 29, ... 60 - 74, ≥ 75), and HIV status (positive and negative), with the HIV-positive stratum further stratified by duration of anti-retroviral therapy (ART) (none, 0 - 6 months, 7 - 12 months, >12 months)

1.2 Demographic model

The demographic model is designed to reflect the demographic projection model (DemProj) of the Spectrum software suite.[6] The demographic model accounts for births, migration, and all-cause mortality. The population is split across the two sex strata (male and female) and 17 age strata split into five-year age groups (0 - 4, 5 - 9, ..., 75 - 79, ≥ 80). Sex is assumed constant throughout, but individuals age up between the different age groups. For this model we have combined the age groups to form 6 strata in fifteen-year age groups (0 - 14, 15 - 29, ... 60 - 74, ≥ 75).

Births are modelled using the crude birth rate from the United Nations (UN) Population Division estimates, whilst also acknowledging the sex distribution of new births, both sourced from UN World Population Prospects estimates.[7] New births are added to the susceptible compartment of the TB model and aging is modelled such that one-fifteenth of the population of each fifteen-year age group moves to the subsequent age group each year.

Migration is calculated by age and sex for each year according to adjusted DemProj estimates.[6] Spectrum provides the annual net migration by gender and the percentage distribution of migration in five-year age groups by gender.[6] The percentages are then summed over three five-year age groups to give the percentage for one fifteen-year age group, and the raw migration numbers are split across the ages by the appropriate percentages. Migration is considered independent of TB or HIV disease status and migrants are distributed across disease states based on the relative size of each compartment.

All-cause mortality is modelled using rates derived from UN Population Division life tables.[7] This is calculated using the proportion of cohort survival over 15 years (i.e., the number of survivors at year 15 compared to the number of survivors at year 0). As TB and HIV deaths are included in background mortality rates, duplicate TB- and HIV-associated deaths are removed at each time point.

1.3 HIV model

The HIV model has been simplified from previous work. [1,5] Individuals are either categorised as HIV-negative or HIV-positive. Those who are HIV-positive are further stratified into four stages of ART duration: none, 0 - 6 months, 7 - 12 months, >12 months). HIV incidence, ART initiation and duration, and HIV-associated mortality are unchanged from previous works, with the exception of the progression through CD4-count categories, as described elsewhere.[1,5]

2 Model equations

Susceptible

$$\frac{d\mathbf{S}_{g,a,h,k,t}}{dt} =$$

$$-\lambda_{g,a,t} \times \mathbf{S}_{g,a,h,k,t}$$

Infection (never previously treated)

$$\frac{d\mathbf{I}_{N_{g,a,h,k,t}}}{dt} =$$

$$\begin{aligned}
\left(\lambda_{g,a,t}\left(1-\alpha_{a,h,k,t}\right)\right) \times &\quad \mathbf{S}_{g,a,h,k,t} \\
-\left(\lambda_{g,a,t}\alpha_{a,h,j,t}\left(1-x_{a,h,k}\right)+\nu_{a,h,k,t}\right) \times &\quad \mathbf{I}_{N_{g,a,h,k,t}} \\
+r_h \times &\quad \mathbf{aTB}_{N_{g,a,h,k,t}}
\end{aligned}$$

Asymptomatic (never previously treated)

$$\frac{d\mathbf{aTB}_{N_{g,a,h,k,t}}}{dt} =$$

$$\begin{aligned}
\lambda_{g,a,t}\alpha_{a,h,k,t} \times &\quad \mathbf{S}_{g,a,h,k,t} \\
+\left(\lambda_{g,a,t}\alpha_{a,h,k,t}\left(1-x_{a,h,k}\right)+\nu_{a,h,k,t}\right) \times &\quad \mathbf{I}_{N_{g,a,h,k,t}} \\
-\left(\theta_h + r_h + \zeta p_{a,d=\mathbf{aTB},g,k}\tau_{h,k,t}\right) \times &\quad \mathbf{aTB}_{N_{g,a,h,k,t}} \\
+\epsilon_h \times &\quad \mathbf{sTB}_{N_{g,a,h,k,t}}
\end{aligned}$$

Symptomatic (never previously treated)

$$\frac{d\mathbf{sTB}_{N_{g,a,h,k,t}}}{dt} =$$

$$\begin{aligned}
\left(\theta_h\right) \times &\quad \mathbf{aTB}_{N_{g,a,h,k,t}} \\
-\left(\epsilon_h + \mu_{I_{a,h,k}} + \zeta p_{a,d=\mathbf{sTB},g,k}\tau_{h,k,t}\right) \times &\quad \mathbf{sTB}_{N_{g,a,h,k,t}}
\end{aligned}$$

Infected (previously treated)

$$\frac{d\mathbf{I}_{P_{g,a,h,k,t}}}{dt} =$$

$$\begin{aligned}
-\left(\lambda_{g,a,t}\alpha_{a,h,j,t}\left(1-x_{a,h,k}\right)+\nu_{a,h,k,t}\right) \times &\quad \mathbf{I}_{P_{g,a,h,k,t}} \\
\left(\zeta p_{a,d=\mathbf{aTB},g,k}\tau_{h,k,t}\right) \times &\quad \mathbf{aTB}_{N_{g,a,h,k,t}} \\
+\left(\zeta p_{a,d=\mathbf{aTB},g,k}\tau_{h,k,t} + r_h\right) \times &\quad \mathbf{aTB}_{P_{g,a,h,k,t}} \\
+\left(\zeta p_{a,d=\mathbf{sTB},g,k}\tau_{h,k,t}\right) \times &\quad \mathbf{sTB}_{N_{g,a,h,k,t}} \\
+\left(\zeta p_{a,d=\mathbf{sTB},g,k}\tau_{h,k,t}\right) \times &\quad \mathbf{sTB}_{P_{g,a,h,k,t}}
\end{aligned}$$

Asymptomatic (previously treated)

$$\frac{d\mathbf{aTB}_{P_{g,a,h,k,t}}}{dt} =$$

$$\begin{aligned}
&+\left(\lambda_{g,a,t}\alpha_{a,h,k,t}\left(1-x_{a,h,k}\right)+\nu_{a,h,k,t}\right)\times && \mathbf{I}_{P_{g,a,h,k,t}}\\
&-\left(\theta_h + r_h + \zeta p_{a,d=\mathbf{aTB},g,k}\tau_{h,k,t}\right)\times && \mathbf{aTB}_{P_{g,a,h,k,t}}\\
&+\zeta p_{a,d=\mathbf{aTB},g,k}\left(1-\tau_{h,k,t}\right))\times && \mathbf{aTB}_{N_{g,a,h,k,t}}\\
&+\epsilon_h \times && \mathbf{sTB}_{P_{g,a,h,k,t}}
\end{aligned}$$

Symptomatic (previously treated)

$$\frac{d\mathbf{sTB}_{P_{g,a,h,k,t}}}{dt} =$$

$$\begin{aligned}
&(\theta_h)\times && \mathbf{aTB}_{P_{g,a,h,k,t}}\\
&-\left(\epsilon_h + \mu_{I_{a,h,k}} + \zeta p_{a,d=\mathbf{sTB},g,k}\tau_{h,k,t}\right)\times && \mathbf{sTB}_{P_{g,a,h,k,t}}\\
&+\zeta p_{a,d=\mathbf{sTB},g,k}\left(1-\tau_{h,k,t}\right))\times && \mathbf{sTB}_{N_{g,a,h,k,t}}
\end{aligned}$$

3 Model parameters

3.1 Force of infection

The force of infection ($\lambda_{g,a,t}$) is a time-dependent parameter, specific to sex (g), age (a), and time (t). It acknowledges the heterogeneous patterns of social contacts between men, women, and children, as well as a constant sex-specific relative risk of infection attributable to biological and/or social factors. The force of infection is as follows:

$$\lambda_{g,a,t} = (z)(cscal)\left(RR_{sex_{g,a}}\right) \times \left(c_{M_{g,a}} \frac{\mathbf{sTB}_{g=M,a\geq15,t} + q\mathbf{aTB}_{g=M,a\geq15,t}}{T_M} + c_{F_{g,a}} \frac{\mathbf{sTB}_{g=F,a\geq15,t} + q\mathbf{aTB}_{g=F,a\geq15,t}}{T_F} + c_{C_{g,a}} \frac{\mathbf{sTB}_{a=0-14,t} + q\mathbf{aTB}_{a=0-14,t}}{T_C}\right)$$

for gender g, age a, and time step t. There are three sex-specific contact parameters: $c_{M_{g,a}}$, $c_{F_{g,a}}$, and $c_{C_{g,a}}$ and these are the average number of contacts with adult men, adult women, and children respectively for the given age group and sex. These are each multiplied by the sum of the number of people of that respective age and sex with symptomatic disease ($\mathrm{sTB}_{M,t}$, $\mathrm{sTB}_{F,t}$, $\mathrm{sTB}_{C,t}$) and the product of the relative infectiousness for asymptomatic disease, q, and the number of people of that respective age and sex with asymptomatic disease ($\mathrm{aTB}_{M,t}$, $\mathrm{aTB}_{F,t}$, $\mathrm{aTB}_{C,t}$) and divided through by the total population of the respective group (T_M, T_F, T_C). The sum of the sex-specific component is then further multiplied by the probability of *Mtb* transmission per respiratory contact between an infectious and an uninfected individual (z) , a scaling factor to account for uncertainty in the total number of contacts ($cscal$) and the relative risk of infection attributable to sex- or gender-based risks ($RR_{sex_{g,a}}$). Prior ranges and data sources for parameters are described below in Supplementary Table 1 and Supplementary Table 5.

Supplementary Table 1: Prior ranges and data sources for parameters related to the force of infection

Parameter	Description	Prior range or set value	References
q	Relative infectiousness of asymptomatic disease compared to symptomatic disease	0.62 - 1	[8]
z	Probability of transmission per respiratory contact between infectious and uninfected individuals	0.1	[1,9–11]
cscal	Scaling factor to adjust the total number of contacts	6 - 25	Assumption

3.1.0.1 Adjustments for heterogeneous mixing

The force of infection acknowledges heterogeneous mixing by sex and age by incorporating the average number of contacts between men (male, aged $\geq$ 15 years), women (female, $\geq$ 15 years), and children (both sexes, age $<$ 15 years). Estimates for the average number of contacts were, where possible, taken from social contact studies. The number of close contacts within a 24-hour period between each participant and each contact group (men, women, boys, and girls) and then we smoothed the matrix to ensure symmetry.[12] Estimates for the average number of contacts in Kenya are based on a social contact survey conducted in urban and rural areas in Mombassa and Kilifi counties in 2015 and the final smoothed contact matrix is shown in Supplementary Table 2. [13]

Supplementary Table 2: The contact matrix used for the Kenya model

Participants	Average number of close contacts		
	Men	Women	Children
Men	4.48	3.12	1.97
Women	1.70	3.57	2.17
Children	1.06	2.15	5.39

A similar survey was conducted in Blantyre, Malawi, and the resulting contact matrix is presented in Supplementary Table 3. [14]

Supplementary Table 3: The contact matrix used for the Malawi model

Participants	Average number of close contacts		
	Men	Women	Children
Men	3.36	1.78	3.81
Women	1.74	3.21	3.90
Children	1.65	1.72	8.40

We found no single social contact survey that covered both sexes and adults and children for either Nigeria or Uganda, so we amalgamated all social contact surveys from countries in Africa to create a more generic matrix for the two countries, as shown in Supplementary Table 4. [13–18]

Supplementary Table 4: The contact matrix used for both the Nigeria and the Uganda models

Participants	Average number of close contacts		
	Men	Women	Children
Men	4.03	3.02	1.40

Participants	Average number of close contacts		
	Men	Women	Children
Women	2.57	4.17	1.90
Children	2.39	3.80	8.70

3.1.0.2 Adjustments for sex- or gender-based risks

The relative risk of infection attributable to sex- or gender-based risks $\left(RR_{sex_{g,a}}\right)$ reflects the accumulation of risks through biological, social, economic, and health determinants that may contribute to men's increased risk of *M.tb* infection relative to women and children.[19] This term incorporates men's higher rates of key TB determinants - smoking (e.g., 16% in men vs. 2% in women in Malawi), alcohol consumption (e.g., 36% in men vs. 18% in women in Nigeria), undernutrition (e.g., 16% in men vs. 9% in women in Kenya), incarceration (0.8% in men vs. 0.04% in women in Uganda) - as well as other factors such as occupational risks, migration, and socialisation patterns.[20–23] In this model, the factors that contribute to these differences are considered constant over time, and the prior for the relative risk of infection in men, relative to women and children is a range of between 1 and 7.5, as shown in Supplementary Table 5.

Supplementary Table 5: Prior ranges and data sources for relative risks of M.tb infection attributable to sex- or gender-based risks

Parameter	Description	Prior range or set value	References
$RR_{sex_{g=M,a\geq15}}$	Relative risk of infection attributable to additional sex- or gender-based risks in men	1 - 7.5	Assumption
$RR_{sex_{g=F,a\geq15}}$	Relative risk of infection attributable to additional sex- or gender-based risks in women	1	Assumption
$RR_{sex_{g,a=0-14}}$	Relative risk of infection attributable to additional sex- or gender-based risks in children	1	Assumption

3.2 Progression to disease

3.2.1 Rapid progression to disease

A proportion of all new infections progress direct to asymptomatic disease ($\alpha_{a,h,k,t}$) and this is a time-dependent parameter specific to age, HIV status, and ART duration.

The proportion of new infections progressing directly to asymptomatic disease in HIV-negative individuals is defined as:

$$\alpha_{a,t} = \alpha_a RR_{BCG_{a,t}}$$

for age a, and time-step t, where α_a is the base proportion of new infections progressing directly to asymptomatic disease and RR_{BCG_a} is the relative risk of

progression from *M.tb* infection to disease attributable to BCG vaccination. The baseline values for α_a are contained in Supplementary Table 6. As children are much more likely to have disseminated or lymph node disease than infectious pulmonary disease, we require that the proportion of infections that rapidly progress to disease in children is less than in adults.[24]

Supplementary Table 6: Prior ranges and data sources for the proportion of infections that progress rapidly to asymptomatic disease

Parameter	Description	Prior range or set value	References
$\alpha_{a\geq 15}$	Base proportion of rapid progression to asymptomatic disease in adults	0.08 - 0.15⁺	[1,5,25–27]
$\alpha_{a=0-14}$	Base proportion of rapid progression to asymptomatic disease in adults	0.08 - 0.15	[1,5,26–28]

⁺$\alpha_{a\geq 15}$ must be greater than $\alpha_{a=0-14}$

3.2.1.1 Adjustments for BCG vaccination

The relative risk of progression from *M.tb* infection to disease attributable to protection from BCG vaccination is defined as follows:

$$RR_{BCG_{a,t}} = \left(1 - BCG_{cov_t} \times BCG_{eff_a}\right)$$

for age a and time t, where BCG_{cov_t} is the coverage of BCG vaccination in infants over time, and BCG_{eff_a} is the efficacy of BCG vaccination.

Coverage of BCG vaccination in infants for each country is shown in Supplementary Figure 3. For each country, vaccine coverage is assumed to increase linearly from 1970 until the data becomes available - 1983 for Kenya, 1981 for the other three countries. The data used are the WHO/UNICEF Estimates of National Immunization Coverage which provides the percentage of live births who have received one dose of the BCG vaccine in a given year. [29] The assumed coverage from 2023 to 2050 represents the likely achievable coverage based om the coverage for the years prior to 2023. BCG coverage is assumed to be equal by sex as a 2009 WHO Expanded Programme on Immunizations survey found similar coverage for boys and girls. [30]

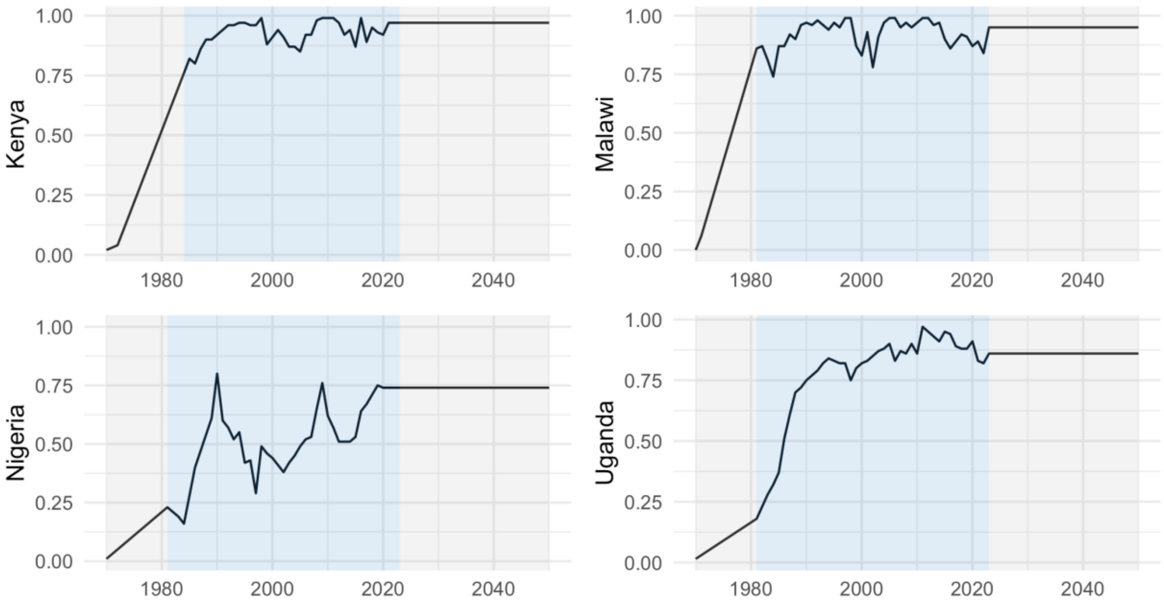

Supplementary Figure 3: The BCG vaccination coverage in infants over time from 1970 to 2050. Data is available for the years shaded in blue, and the years shaded in grey are assumed coverage.

The efficacy of BCG vaccination is assumed to vary by age, as shown in Supplementary Table 7.

Supplementary Table 7: Prior ranges and data sources for the efficacy of BCG vaccination

Parameter	Description	Prior range or set value	References
$BCG_{eff_{a\geq 15}}$	Efficacy of BCG vaccination in ages ≥ 15	0	Assumption
$BCG_{eff_{a=0-14}}$	Efficacy of BCG vaccination in ages 0 - 14 years	0.39 - 0.72	[1,5,31]

3.2.1.2 HIV-positive and ART strata

The proportion of new infections developing primary disease is adjusted for individuals with HIV as follows:

$$\alpha_h = \alpha \times RR_{1_\alpha}$$

where α is the baseline parameter for rapid progression, and RR_{1_α} is the risk ratio of progression for individuals with HIV. The value for the risk ratio is in Supplementary Table 8.

ART reduces the effect of HIV infection on rapid progression to disease, bringing the value closer to that for an individual without HIV:

$$\alpha_{h,k}^{A} = \max(\alpha_h^H \times (1 - ART_k), \alpha)$$

where α is the baseline parameter for rapid progression and RR_{1_α} is the relative risk of progression for individuals with HIV. Subscripts h and k are the HIV status and ART duration respectively, and superscripts A and H refer to HIV-positive individuals on ART and not on ART respectively. The values of these HIV- and ART-specific parameters are in Supplementary Table 8.

Supplementary Table 8: Prior ranges and data sources for the risk ratio associated with being HIV-positive and the reduction of risk from ART

Parameter	Description	Prior range or set value	References
RR_{1_α}	The risk ratio for new infections rapidly progressing to asymptomatic disease due to HIV co-infection	2.11 - 20	[1,5,27,32]
$ART_{TB_{t<6}}$	Protective effect of ART treatment against developing disease for treatment duration < 6 months	0.16 - 0.27	[5,33]
$ART_{TB_{t=6-12}}$	Protective effect of ART treatment against developing disease for treatment duration 6 - 12 months	0.30 - 0.73	[5,33]
$ART_{TB_{t\geq12}}$	Protective effect of ART treatment against developing disease for treatment duration ≥ 12 months	0.54 - 0.92[+]	[5,33]

[+] $ART_{TB_{t\geq12}}$ must be greater than $ART_{TB_{t=6-12}}$

3.2.2 Progression from infection

There is a fixed rate of progression from infection to disease, independent of time since infection, $\nu_{a,h,k}$, that is dependent on age, HIV status, and ART duration. The progression rate in HIV-negative individuals is ν_a, dependent only on age. As before, as children are much more likely to have disseminated or lymph node disease than infectious pulmonary disease, we require that the proportion of infections that progress to disease in children is less than in adults.[24] The relevant values for progression from infection are included in Supplementary Table 9

3.2.2.1 HIV-positive and ART strata

The rate of progression to asymptomatic disease is adjusted for HIV in the same way as rapid progression in Section 3.2.1.2, using the parameter RR_{1_ν}. The prior range and references for this parameter are included in Supplementary Table 9.

The rate of progression to asymptomatic disease is adjusted for ART duration in the same way as rapid progression in Section 3.2.1.2, using the values described in Supplementary Table 8.

Supplementary Table 9: Prior ranges and data sources for the annual rate of progression to asymptomatic disease in HIV-negative individuals and the risk ratio associated with being HIV-positive

Parameter	Description	Prior range or set value	References
$\nu_{a\geq 15}$	The annual rate of progression from infection to asymptomatic disease in ages ≥ 15	0.0001 - 0.0025	[1,5,25–27,34]
$\nu_{a=0-14}$	The annual rate of progression from infection to asymptomatic disease in ages 0 - 14	0.0001 - 0.0025	[1,5,25–27,34]
RR_{1_ν}	The risk ratio for infection progression or reactivation in HIV-positive individuals	2.11 - 20	[1,5,25,27,32,35]

3.2.3 Reinfection

Previous infection confers some protection against reinfection, $x_{a,h,k}$, a constant specific to age, HIV status, and ART duration. The progression rate in HIV-negative individuals is x_a, dependent only on age. The relevant values for progression from infection are included in Supplementary Table 10

3.2.3.1 HIV-positive and ART strata

The rate of progression to asymptomatic disease is adjusted for HIV in the same way as rapid progression in Section 3.2.1.2, using the parameter RR_{1_x}. The prior range and references for this parameter are included in Supplementary Table 10.

The rate of progression to asymptomatic disease is adjusted for ART duration in the same way as rapid progression in Section 3.2.1.2, using the values described in Supplementary Table 8.

Supplementary Table 10: Prior ranges and data sources for the annual rate of progression to asymptomatic disease in HIV-negative individuals and the risk ratio associated with being HIV-positive

Parameter	Description	Prior range or set value	References
$x_{a\geq 15}$	Proportion of protection against progression to asymptomatic disease due to previous infection in adults	0.37 - 0.9	[1,5,25,26,36,37]
$x_{a=0-14}$	Proportion of protection against progression to asymptomatic disease due to previous infection in children	0.37 - 0.9	[1,5,25,26,36,37]
RR_{1_x}	The risk ratio for protection from reinfection in HIV-positive individuals	0 - 1	[1,5,25,26,37]

3.3 Progression through disease

Progression from asymptomatic to symptomatic disease occurs at a rate θ. This parameter is only dependent on HIV status and is described in Supplementary Table 11.

Although people living with HIV tend to have lower smear grade and less cavitation than HIV-negative people, this seems not to align with symptoms as the WHO 4-symptom screen has a high sensitivity in people living with HIV, and recent understanding of asymptomatic TB has found that there is little difference between the proportion of people without symptoms regardless of HIV status, and there may even be an increasing prevalence of symptomatic TB with decreasing CD4 counts. [38–40]The rate of progression from asymptomatic to symptomatic disease is adjusted for HIV in the same way as rapid progression in Section 3.2.1.2, using the parameter RR_{1_θ}. The prior range and references for this parameter are included in Supplementary Table 11.

There are no adjustments for ART within the progression from asymptomatic to symptomatic disease.

Supplementary Table 11: Prior ranges and data sources for the annual rate of progression from asymptomatic disease to symptomatic disease in HIV-negative individuals and the risk ratio associated with being HIV-positive

Parameter	Description	Prior range or set value	References
θ	Rate of progression from asymptomatic to symptomatic disease in HIV-negative individuals	0.56 - 0.94	[2,3,5]
RR_{1_θ}	Risk ratio for progression from asymptomatic to symptomatic disease in HIV-positive individuals	2.11 - 20	[1,5,25,27,32,35]

3.4 Regression through disease

Regression from symptomatic to asymptomatic disease is described by ϵ_h, a parameter only dependent on HIV status as described in Supplementary Table 12.

The rate of regression from symptomatic to asymptomatic disease in HIV-positive individuals is not adjusted in the same way. Instead it has a prior range, as shown in Supplementary Table 11, with a lower bound half that of the lower bound of $\epsilon_{h=HIV-}$ and a restriction that $\epsilon_{h=HIV+} < \epsilon_{h=HIV-}$.

There are no adjustments for ART within the regression from symptomatic to asymptomatic disease.

Supplementary Table 12: Prior ranges and data sources for the annual rate of regression from symptomatic disease to asymptomatic disease in HIV-negative individuals and HIV-positive individuals

Parameter	Description	Prior range or set value	References
$\epsilon_{h=HIV-}$	Rate of regression from symptomatic to asymptomatic disease in HIV-negative individuals	0.46 - 0.73	[2,3,5]
$\epsilon_{h=HIV+}$	Rate of regression from symptomatic to asymptomatic disease in HIV-positive individuals	0.23 - 0.73^{+}	[2,3,5]

$^{+}\epsilon_{HIV}$ must be les than ϵ

3.5 Self-cure

Self-cure from disease, without treatment, occurs from asymptomatic disease, and returns individuals to infection. Self-cure is described by r_h, a time-independent parameter, dependent on HIV status, as shown in Supplementary Table 13.

Supplementary Table 13: Prior ranges and data sources for the parameters describing self-cure from disease

Parameter	Description	Prior range or set value	References
$r_{h=HIV-}$	Rate of self-cure from asymptomatic disease in HIV-negative individuals	0.1 - 0.306	[2,3,5,26,27,41,42]
$r_{h=HIV+}$	Rate of self-cure from asymptomatic disease in HIV-positive individuals	0.06 - 0.16^{+}	[5,26,37]

$^{+}r_{h=HIV+}$ must be less than $r_{h=HIV-}$

3.6 Care cascade

The care cascade is represented by three separate values: access to care, ζ, an adjustment of access to care for different strata, $p_{a,d,g,k}$, and treatment completion, $\tau_{h,k,t}$.

$$R_{a,d,g,h,k,t} = \zeta \times p_{a,d,g,k} \times \tau_{h,k,t}$$

3.6.1 Access to care

Access to care is informed by the treatment coverage in each country, which is a country-specific, time-dependent parameter. Spikes or dips in notifications reflect changes in programmatic activities (screening campaigns, diagnostic algorithms, etc.) over time. Data for each country is available from 2000 onwards, and before 2000 we assumed the growth of access to care based on the change in treatment coverage in the available time period from 2000 to 2022.[43] For Kenya we chose an approximately

S-shaped curve to bring treatment coverage up from a low starting point, but then to stabilise between 2000 and 2015. Malawi had a more consistent change with time, so we chose a linear increase from a similarly low starting point. Nigeria had very low treatment coverage in 2000, and so we assumed a flat but low coverage for all the years prior to 2000. Uganda had consistently high treatment coverage from 2000 to 2020, with an increase in 2022, and prior to 2000 we assumed a low flat coverage until 1990 followed by a gradually increasing increase to meet the trajectory of coverage in 2000. All these trends can be seen in Supplementary Figure 4. Projecting into the future, we used the last reported value in 2023 and assumed a fixed level of access to care going forwards, but also conducted a supplementary analysis where access to care increased in-line with the expectations of Kenya's National Strategic Plan.[44]

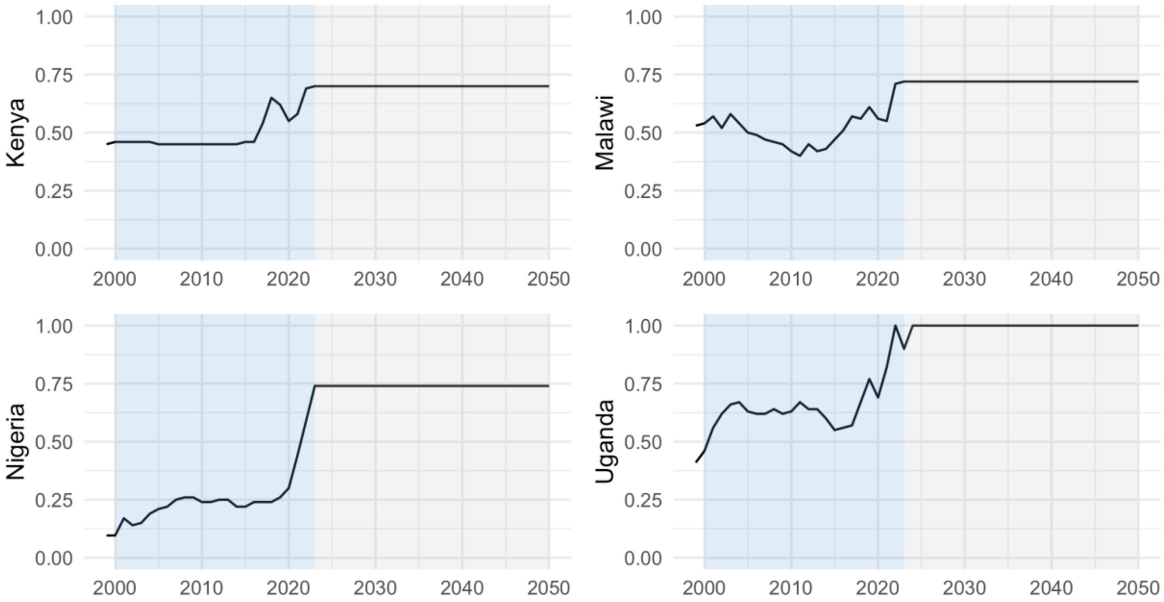

Supplementary Figure 4: Access to care over time, with assumed access in the grey area and treatment coverage data in the blue area.

3.6.1.1 Adjustments to treatment coverage

Access to care varies across the population, and by using treatment coverage to estimate this parameter, we have introduced further uncertainty. To account for this, we introduced time-independent modifiers to the access to care value dependent on the demographic and disease state group, $p_{a,d,h,k}$. These are then multiplied with the access to care parameter to represent the overall treatment coverage at that time for the given subgroup. The values for these modifiers are in Supplementary Table 14.

$$p_{a,d,h,k} = \left(\begin{cases} (1+p_a) & \text{if } a < 15 \\ (1+p_k) & \text{if on ART} \\ (1+p_d) \times (1+p_g) & \text{otherwise} \end{cases} \right)$$

Supplementary Table 14: Prior ranges for the demographic- and disease-specific modifiers to treatment coverage

Parameter	Description	Prior range or set value	References
$p_{d=\mathbf{sTB}}$	Adjustment to treatment coverage for symptomatic disease in HIV-negative individuals and HIV-positive individuals who are not on ART	0	Assumption
$p_{d=\mathbf{aTB}}$	Adjustment to treatment coverage for asymptomatic disease in HIV-negative individuals and HIV-positive individuals who are not on ART	$-1 - 0$	Assumption
$p_{k=ART}$	Adjustment to treatment coverage for symptomatic and asymptomatic disease in HIV-positive individuals who are receiving ART and have been for any duration	$0 - 1$	Assumption
$p_{k=noART}$	Adjustment to treatment coverage for symptomatic and asymptomatic disease in HIV-positive individuals who are not receiving ART	0	Assumption
$p_{g=M}$	Adjustment to treatment coverage for men, regardless of disease state	$-0.75/0.5 - 0.5^{+}$	Assumption
$p_{g=F}$	Adjustment to treatment coverage for women, regardless of disease state	$-0.5 - 0.5/1$	Assumption
$p_{a=0-14}$	Adjustment to treatment coverage for children, regardless of disease state	$-1 - 0$	Assumption
$p_{a\geq15}$	Adjustment to treatment coverage for adults, regardless of disease state	0	Assumption

$^{+}p_{g=M}$ must be less than $p_{g=F}$

3.6.2 CAST in Uganda

From 2022, Uganda have conducted a twice-yearly active case finding strategy that significantly increased their case-notification numbers. The first two rounds in 2022 screened over 1.25 million and over 5 million people respectively, creating an impact much larger than that of the general care cascade pathway in the model.[45] We therefore adjusted the model with further additions to the demographic-specific modifiers. As this is community-based screening, people on ART are no more likely to be screened than anyone else, unlike in classic treatment circumstances where people on ART are regularly attending clinics.[45] As this programme was only focussed on symptom screening, there was also no adjustment for asymptomatic disease.[45] Case notifications between 2021 (before CAST was implemented) and 2022 (the first year of CAST), increased by 38% in women, 29% in men, and 3% in children.[43] Community screening strategies are rarely uniform across the population, with screening often less

likely to meet women as seen here and in examples from Zimbabwe where door-to-door screening reached 48% fewer men than women and mobile van screening reached 18% fewer men than women.[46] To account for this, adjustments to the access to care were included for men, women, and children, from 2022 onwards with the adjustment to the care cascade equation as below, and the prior values for $f_{a,g}$ in Supplementary Table 15.

$$R_{a,d,g,h,k,t} = \zeta \times (p_{a,d,g,k} + f_{a,g}) \times \tau_{h,k,t}$$

Supplementary Table 15: Prior ranges for the parameters to adjust the care cascade to represent the CAST programme in Uganda

Parameter	Description	Prior range or set value	References
$f_{a=0-14}$	Adjustment to care cascade for children	0 - 1.5	Assumption
$f_{a\geq 15,g=M}$	Adjustment to care cascade for men	0 - 2	Assumption
$f_{a\geq 15,g=F}$	Adjustment to care cascade for women	1 - 4	Assumption

3.7 Treatment completion

Treatment completion is a time-dependent parameter. Data is available on treatment completion for HIV-negative individuals from 1995 to 2022, and for HIV-positive individuals it is available from 2007 in Kenya, 2015 in Malawi, 2009 in Nigeria, and 2013 in Uganda.[47] In the years before 1995, we have assumed that treatment success for HIV-negative individuals is lower and constant, with values for each country dependent on the available data during the period of 1995 to 2022. Where data is available, treatment completion in HIV-positive individuals tracks closely, albeit slightly lower, to treatment completion for HIV-negative individuals. As such, when filling in the years before data was available, we maintained a gap of minimum 4% between HIV-negative and HIV-positive before 1995, and then between 1995 and the first available data point, treatment completion for HIV-positive individuals could not be higher than the lowest value achieved for HIV-negative individuals in the future years. The resulting time trends can be seen in Supplementary Figure 5. We have not separated treatment completion rates between men and women as, based on the limited data available from the four countries, there is not much difference. In 2022, the treatment success rate was 90% for women and 88% for men in Kenya, and 91% for women and 89% for men in Uganda.[48] While sex-disaggregated data are not available for Malawi or Nigeria for 2022, the overall treatment success rates were high ($\geq$ 90% for both countries).[49]

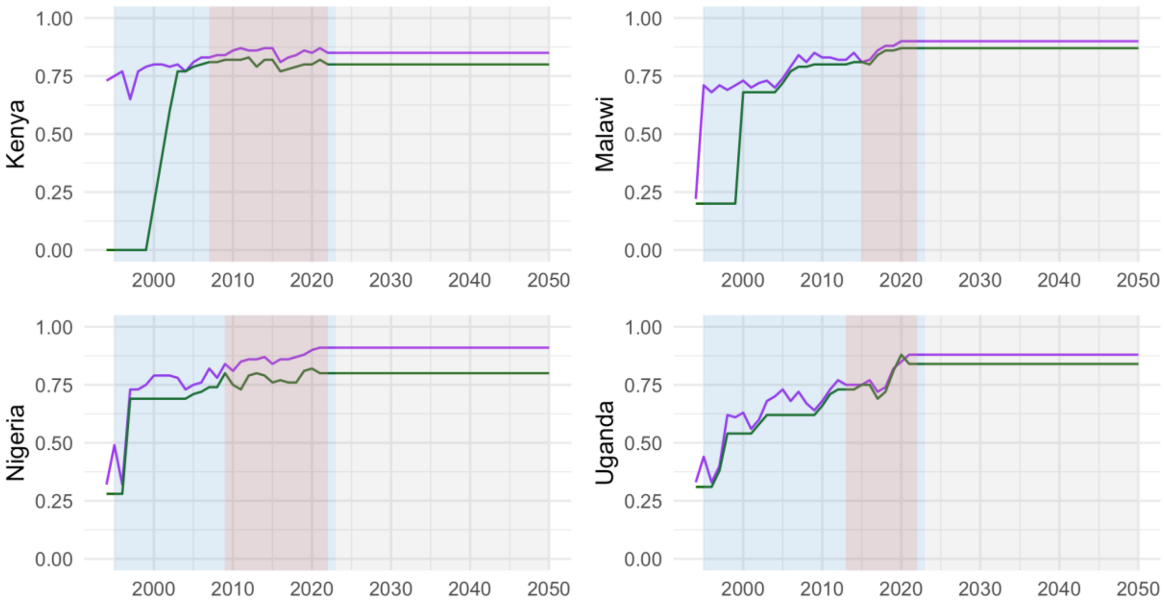

Supplementary Figure 5: The treatment completion for each of the four countries over time. The purple line is for HIV-negative treatment completion and the green line is for HIV-positive treatment completion. The blue areas are where data is available for HIV-negative treatment completion, and the red area is where data is available for both HIV-negative and HIV-positive treatment completion

3.8 Mortality

Disease-specific mortality is only possible from symptomatic disease ($\mu_{\mathbf{sTB}_{a,h,k}}$), with a time-independent parameter that is specific to age, HIV status, and ART duration. The prior range for TB mortality in HIV-negative individuals is shown in Supplementary Table 16.

The mortality rate for children ages 0-14 is unchanged to that in adults, but there is scope in the model to adjust it:

$$\mu_{\mathbf{sTB}_{a=0-14}} = \mu_{\mathbf{sTB}} RR_{\mu_{\mathbf{sTB}_0}}$$

where μ_I is the baseline mortality for HIV-negative adults, and $RR_{\mu_{I_0}}$ is the relative risk of symptomatic disease mortality in children. $RR_{\mu_{I_0}}$ is fixed at 1 in this model, as shown in Supplementary Table 16.

References [2,3,5,50] and [5]

Supplementary Table 16: Prior ranges for the parameters for TB mortality

Parameter	Description	Prior range or set value	References
$\mu_{\mathbf{sTB}}$	Annual rate of TB mortality from symptomatic disease	0.26 - 0.449	Assumption

Parameter	Description	Prior range or set value	References
$RR_{\mu_{\mathbf{sTB}_0}}$	Relative risk of TB mortality from symptomatic disease in children ages 0 - 14	1	Assumption

3.8.0.1 HIV-positive and ART strata

The mortality rate in HIV-positive individuals can change compared to HIV-negative individuals:

$$\mu_{\mathbf{sTB}_{h=HIV+}} = \mu_{\mathbf{sTB}} RR_{\mu_H}$$

where μ_I is the baseline mortality for HIV-negative adults, and RR_{μ_H} is the relative risk of symptomatic disease mortality in HIV-positive individuals. The prior range for RR_{μ_H} is shown in Supplementary Table 17.

The mortality rate is adjusted for ART duration in the same way as rapid progression in Section 3.2.1.2, using the values described in Supplementary Table 17.

Supplementary Table 17: Prior ranges and data sources for the risk ratio associated with being HIV-positive and the reduction of risk from ART

Parameter	Description	Prior range or set value	References
RR_{μ_H}	Risk ratio of TB mortality from symptomatic disease in HIV-positive individuals	1/1.5 - 2.5/3	[5]
$ART_{\mu_{\mathbf{sTB}_{t<6}}}$	Protective effect of ART treatment against TB/HIV death for treatment duration < 6 months	0.11 - 0.28	[1,5,33]
$ART_{\mu_{\mathbf{sTB}_{t=6-12}}}$	Protective effect of ART treatment against TB/HIV death for treatment duration ≥ 12 months	0.51 - 0.75	[1,5,33]
$ART_{\mu_{\mathbf{sTB}_{t\geq 12}}}$	Protective effect of ART treatment against TB/HIV death for treatment duration 12 months	0.64 - 0.95^{+}	[1,5,33]

$^{+}ART_{\mu_{\mathbf{sTB}_{t\geq 12}}}$ must be greater than $ART_{\mu_{\mathbf{sTB}_{t=6-12}}}$

4 Model implementation

The model is initialised with the 1970 population, with sex and age structure and 50 symptomatic, treatment-naive TB cases in the age group 30 - 44 years. The model is then run for 200 years with all parameters set at the 1970 values and no HIV.

The resulting population is then re-scaled to the 1970 population and the model is run through to 2050 with time-dependent parameter values and HIV and ART.

The model is implemented in R.[51] Model equations are implemented as ordinary differential equations with a time step of 0.5 years and are solved using the fourth order Runge-Kutte integration method.[52]

5 Calibration

5.1 Epidemiological calibration targets

5.1.1 Data sources

Each of these four countries has conducted a prevalence survey which provides empirical data on TB prevalence targets for the respective year of the survey in each country.[53–56] Male to female ratios and prevalence to notification ratios were similarly sourced for the respective prevalence survey years, using reported case notifications from each country in the same year.[57] Targets also included incidence and mortality estimates for the overall population in 2010 and 2020 to ensure that the model calibration followed the time trend observed in the countries, for incidence and mortality in individuals with HIV in 2020, and for incidence in men and women in 2022 (as estimates are only disaggregated by sex for the most recent year). [43,58–60] These are derived from WHO models, with the gender split in incidence estimates informed by national prevalence surveys across the region.[4,61] Calibration targets also included overall reported case notifications in 2010 and 2022, and among men, women, children, and HIV-positive individuals in 2022. [43,59,60,62–65]

Supplementary Table 18: The country-level targets used to calibrate the models

<table>
<tr><th rowspan="2">Measure</th><th rowspan="2">Population</th><th rowspan="2">Year</th><th colspan="4">Target Ranges</th><th rowspan="2">Notes</th><th rowspan="2">References</th></tr>
<tr><th>Kenya</th><th>Malawi</th><th>Nigeria</th><th>Uganda</th></tr>
<tr><td rowspan="4">Prevalence per 100k</td><td>Total</td><td rowspan="6">K: 2016
M: 2014
N: 2012
U: 2015</td><td>347-504</td><td>258-468</td><td>239-406</td><td>191-315</td><td></td><td rowspan="3">[53–56]</td></tr>
<tr><td>Men</td><td>656-962</td><td>335-757</td><td>538-965</td><td>554-914</td><td></td></tr>
<tr><td>Women</td><td>258-460</td><td>246-501</td><td>213-505</td><td>109-248</td><td></td></tr>
<tr><td>MtoF</td><td>1.79-2.83</td><td>0.94-1.87</td><td>1.52-2.96</td><td>2.84-5.81</td><td></td><td>[57]</td></tr>
<tr><td rowspan="2">Prevalence-to-notification ratio</td><td>Men</td><td>3.04-4.46</td><td>1.65-4.05</td><td>4.99-9.51</td><td>2.81-4.64</td><td></td><td rowspan="2">[57]</td></tr>
<tr><td>Women</td><td>2.5-4.46</td><td>1.25-3.14</td><td>2.51-6.74</td><td>1.24-2.83</td><td></td></tr>
<tr><td rowspan="5">Incidence per 100k (annual)</td><td rowspan="2">Total</td><td>2010</td><td>174-1080</td><td>55-870</td><td>143-311</td><td>135-301</td><td></td><td rowspan="2">[43]</td></tr>
<tr><td>2020</td><td>155-367</td><td>73-225</td><td>143-311</td><td>120-298</td><td></td></tr>
<tr><td>Men</td><td>2022</td><td>139-769</td><td>10.6-466.2</td><td>175.3-682.3</td><td>102-651.5</td><td rowspan="2">Section 5.1.2.1</td><td>[58,59]</td></tr>
<tr><td>Women</td><td>2022</td><td>64-346</td><td>6.5-293.3</td><td>100.4-390.4</td><td>61.4-389.5</td><td>[58,60]</td></tr>
<tr><td>HIV-positive</td><td>2020</td><td>37-90</td><td>34-101</td><td>0.1-0.15</td><td>39-98</td><td></td><td>[43]</td></tr>
<tr><td>Proportion of incidence</td><td>Children (%)</td><td>2022</td><td>-</td><td>-</td><td>11-24</td><td>-</td><td>Only Nigeria</td><td>[58]</td></tr>
<tr><td rowspan="3">Mortality per 100k (annual)</td><td rowspan="2">Total</td><td>2010</td><td>124-247</td><td>77-233</td><td>61-120</td><td>39-76</td><td></td><td rowspan="3">[43]</td></tr>
<tr><td>2020</td><td>41-82</td><td>26-57</td><td>46-105</td><td>28-55</td><td></td></tr>
<tr><td>HIV-positive</td><td>2020</td><td>13-32</td><td>13-38</td><td>6-14</td><td>13-29</td><td></td></tr>
</table>

Measure	Population	Year	Target Ranges				Notes	References
			Kenya	Malawi	Nigeria	Uganda		
Notifications per 100k (annual)	Total	2010	191-298	114-172	41-87	106-159	Section 5.1.2.2	[43,62–64]
		2019	-	-	-	123-184	Section 5.1.2.2 Only Uganda	
	Men	2019	-	-	-	254-382	Section 5.1.2.3 Only Uganda	[59,62–65]
	Women	2019	-	-	-	139-208		[60,62–65]
	Children	2019	-	-	-	33.1-49.7		[59,60,62,65]
Proportion of total notifications	HIV-positive (%)	2019	-	-	-	0.313-0.47		[62,65]
Notifications per 100k (annual)	Total	2022	131-205	70-106	103-215	159-240	Section 5.1.2.2	[43,62–64]
	Men	2022	253-395	144-217	194-406	308-462	Section 5.1.2.3	[59,62–65]
	Women	2022	120-188	84-127	142-298	181-271		[60,62–65]
	Children	2022	-	-	-	57.1-85.7	Section 5.1.2.3 Only Uganda	[59,60,62,65]
Proportion of total notifications	Children (%)	2022	0.096-0.144	0.064-0.096	0.056-0.084	-	Section 5.1.2.4	[62,65]
	HIV-positive (%)	2022	0.192-0.288	0.384-0.576	0.045-0.068	26.4-39.6		[62,65]

5.1.2 Calculation notes

5.1.2.1 Incidence in men and women

The WHO provides the estimated incidence of TB disease for men and women within the *TB incidence disaggregated by age group, sex, and risk factor* file, where this data is provided for the latest year available (2022).[58] These are raw numbers for the lower and upper bounds, and we transformed them into incidence per 100,000 population to use in the model. The UN World Population Prospects provides estimates for the population split by age groups and sex.[59,60] The "middle variant" estimate for future projections matches the total population estimate reported by the WHO, so we have used the "middle variant" estimate for the sex-disaggregated populations.[43]

The calculation for incidence in men and women is then:

$$\frac{\text{Incidence Estimate}}{\text{Population estimate} \times 1000} \times 100{,}000$$

using the data as presented in Supplementary Table 19

Supplementary Table 19: Calculations for the incidence targets by sex

Country	Sex	Lower incidence estimates	Upper incidence estimates	Population estimates	Lower estimate/100k	Upper estimate/100k
Kenya	*Male*	23,000	127,000	16,524.716	139.2	768.5
	Female	11,000	59,000	17,074.203	64.4	345.6
Malawi	*Male*	590	26,000	5,577.175	10.6	466.2
	Female	400	18,000	6,136.975	6.5	293.3
Nigeria	*Male*	110,000	428,000	62,732.636	175.3	682.3
	Female	62,000	241,000	61,738.365	100.4	390.4
Uganda	*Male*	13,000	83,000	12,740.267	102.0	651.5
	Female	8,200	52,000	13,350.299	61.4	389.5

5.1.2.2 Total notifications

Notification data is reported as a point value of notifications reported per year, and thus do not take into account under-reporting (for example due to private healthcare diagnoses) or over-reporting (due to misdiagnoses where someone does not have TB), so there may be some uncertainty in the reported numbers.[63,64] The WHO provides notification numbers for each country from 1980.[62] We have used notifications from 2010, but to avoid any impact of COVID-19 on TB notifications, we have used 2022 as the second time-point instead of 2020. The notifications values are transformed to notifications per 100,000 by using the estimated total population for each year, provided by the WHO.[43]

To incorporate uncertainty into our targets, each country has been given an upper and lower multiplier. For countries with no, or limited, under-reporting, this is $\pm 20\%$ using multipliers of 1.2 and 0.8 respectively (Malawi and Uganda). For Kenya and Nigeria, the lower estimate is still -20% but the upper estimate takes into account the under-reporting. Kenya is reported to have 20% under-reporting, and so to bring this up to 100% we have used an upper-estimate multiplier of 1.25.[63] Nigeria is reported to have 40% under-reporting, which requires an upper-estimate multiplier of 1.67.[64]

The calculation for notification estimates is:

$$\left(\frac{\text{reported notifications}}{\text{estimated population}} \times 100{,}000\right) \times \text{multiplier}$$

Supplementary Table 20: Calculations for the notifications targets for each country

Country	Year	Notifications	Population	Lower multiplier	Upper multiplier	Point value	Lower estimate	Upper estimate
Kenya	2010	99,272	41,517,895	0.8	1.25	239.11	191.3	298.9
	2022	88,690	54,027,487			164.16	131.3	205.2

Country	Year	Notifications	Population	Lower multiplier	Upper multiplier	Point value	Lower estimate	Upper estimate
Malawi	2010	21,092	14,718,422	0.8	1.20	143.30	114.6	172.0
	2022	18,025	20,405,317			88.33	70.7	106.0
Nigeria	2010	84,121	160,952,853	0.8	1.67	52.26	41.8	87.3
	2022	282,184	218,541,212			129.12	103.3	215.6
	2010	42,885	32,341,728	0.8		132.60	106.1	159.1
Uganda	2019	65,897	42,949,080		1.20	153.43	122.7	184.1
	2022	94,457	47,249,585	0.8		199.91	159.9	239.9

5.1.2.3 Notifications in men and women (and children in Uganda)

The proportion of notifications in men and women are available from the WHO case notifications file and are reported on the WHO TB profile for each country.[62,65]

To calculate the range of notifications per 100,000 for each target, we take the total number of notifications, and the proportion of those notifications that were in men and women aged 15 and older, and multiply each to fund the number of notifications for men and women respectively. This number of notifications is then divided by the respective population size for each sex.[59,60] As with total notifications, the range is calculated using lower and upper multipliers to account for potential over- and under-reporting.[63,64]

The calculation for sex-specific notifications is:

$$\left(\frac{\text{proportion of notifications} \times \text{total reported notifications}}{\text{population of sex}} \times 100000\right) \times \text{multiplier}$$

Supplementary Table 21: Calculations for notifications by sex for each country, and age for Uganda

Sex	Country	Year	Proportion	Notifications	Population	Lower multiplier	Upper multiplier	Point value	Lower estimate	Upper estimate
**Male**	***Kenya***	2022	0.59	88,690	16,524,716	0.8	1.25	316.66	253.3	395.8
	Malawi	2022	0.56	18,025	5,577,175	0.8	1.20	180.99	144.8	217.2
	Nigeria	2022	0.54	282,184	62,732,636	0.8	1.67	242.90	194.3	405.6
	Uganda	2019	0.54	65,897	11,265,950	0.8	1.20	315.86	252.7	379.0
		2022	0.52	94,457	12,740,268	0.8	1.20	385.53	308.4	462.6
**Female**	***Kenya***	2022	0.29	88,690	17,074,204	0.8	1.25	150.64	120.5	188.3
	Malawi	2022	0.36	18,025	6,136,975	0.8	1.20	105.74	84.6	126.9
	Nigeria	2022	0.39	282,184	61,738,365	0.8	1.67	178.26	142.6	297.7

Sex	Country	Year	Proportion	Notifications	Population	Lower multiplier	Upper multiplier	Point value	Lower estimate	Upper estimate
	Uganda	2019	0.31	65,897	11,840,341	0.8	1.20	172.53	138.0	207.0
		2022	0.32	94,457	13,350,299	0.8	1.20	226.41	181.1	271.7
**Children**	***Uganda***	2019	0.12	65,897	19,842,790	0.8	1.20	41.41	33.1	49.7
		2022	0.16	94,457	21,159,019	0.8	1.20	71.43	57.1	85.7

5.1.2.4 Proportion of notifications in children and people living with HIV

As in Section 5.1.2.3, the proportion of notifications in children and for people living with HIV are available to calculate from the WHO case notifications file and are reported on the WHO TB profile app.[62,65] These measures have been kept as a proportion of all notifications for the targets. As before, error bounds of $\pm 20\%$ have been aded to the point values available, as shown in Supplementary Table 22.

Supplementary Table 22: Calculations of proportion of notifications for children and people living with HIV

	**Notifications in children**			**Notifications in people living with HIV**		
	**Point value**	**Lower estimate**	**Upper estimate**	**Point value**	**Lower estimate**	**Upper estimate**
Kenya	12	9.6	14.4	24.0	19.2	28.8
Malawi	8	6.4	9.6	48.0	38.4	57.6
Nigeria	7	5.6	8.4	5.7	4.6	6.8
Uganda				33.0	26.4	39.6

5.2 Calibration process

Each country was calibrated separately using the parameter ranges discussed above in section Section 3 to the unique target ranges for each country from Supplementary Table 18. We calibrated each model in R using the hmer package (history matching and emulation in R).[51,66,67] For each country, we ran an initial wave of test parameters from two sets of latin hypercube sampling (LHS) across the parameter space, one set for training and one set for validation. Each set used 10 times the number of parameters for each country, giving a total of 640 parameter sets for Kenya, Malawi, and Nigeria, and 700 parameter sets for Uganda across both the training and validation sets. Using the output from the training parameter sets, we created emulators for the model. It was possible to check the accuracy of the model by comparing the output of the validation sets against the emulators from the training sets.

As outlined in Section 3, there are some parameter constraints, specified below in (1).

$\alpha_{a\geq15} > \alpha_{a=0-14}$	rate of progression to disease is higher in adults
$r > r_H$	rate of self-cure is higher in HIV-negative individuals
$eps > eps_H$	rate of disease regression is higher in HIV-negative individuals
$ART_{TB_{t\geq12}} > ART_{TB_{t=6-12}}$	reduction in disease risk greater for longer ART durations
$RR_{\mu_{\mathbf{sTB}_{t\geq12}}} > RR_{\mu_{\mathbf{sTB}_{t=6-12}}}$	reduction in mortality risk is greater for longer ART durations
$p_{g=F} > p_{g=M}$	treatment coverage for women is higher than for men
$p_{g=M} > p_{d=\mathbf{aTB}}$	treatment coverage for men is higher than for asymptomatic disease
$p_{k=ART} > p_{g=F}$	treatment coverage for people on ART is higher than for women

Non-implausible points were generated based on the emulators created from the training data and were checked against these parameter constraints. Again, we were looking for 20 times the number of parameters to feed into the next wave of the process. For the next, and each successive waves, those non-implausible points were run through the model and then randomly sampled to split in half for the training and validation data.

We ran this process until more than 40% of the parameter sets hit all targets. After a minimum of 5 waves, we set a further argument in the non-implausible points selection of `nth = 1` which requires that the non-implausible points selection only aims for parameter sets that hit all of the targets rather than allowing for one or two of the targets to be missed. This allowed for the full space to be explored before refining the parameter space.

Once the required number of targets were hit, we ran another wave and used the emulators from the final wave to sample parameters for the model. We ran three or four consecutive parameter selections from the same emulators, each selecting 1000 parameters. We ran these parameters through the model to select parameter sets that hit the targets, and then randomly sampled 1000 parameter sets that we have used for the remainder of the analysis. The distribution of the chosen parameter sets against the priors for each country are shown in Section 5.2.1

5.2.1 Successful calibrations

For each of the countries we present the wave output of the hmer calibration, showing the progress of each country's calibration over successive waves. We then present a plot comparing the priors and posteriors for each parameter and a table comparing the numerical values with the median and 95%UIs for the posterior parameters. Finally, we show the time series plots for each of the calibration targets.

5.2.1.1 Kenya

Over ten waves, the calibration for Kenya reached 535/640 of the tested parameter sets hitting all 20 targets (Supplementary Figure 6). We used the emulators from wave 10 to find suitable parameter sets.

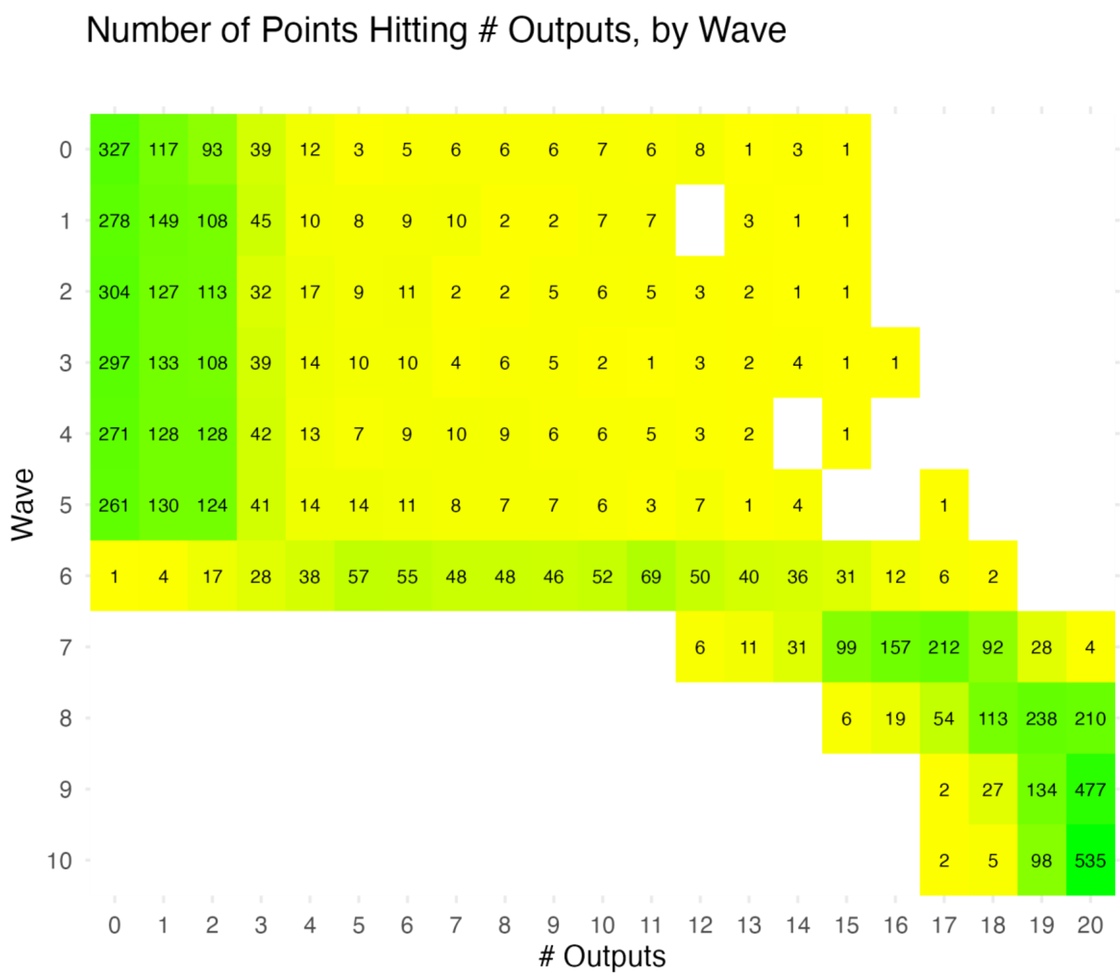

Supplementary Figure 6: The wave progression of the hmer process for the calibration of the Kenyan model

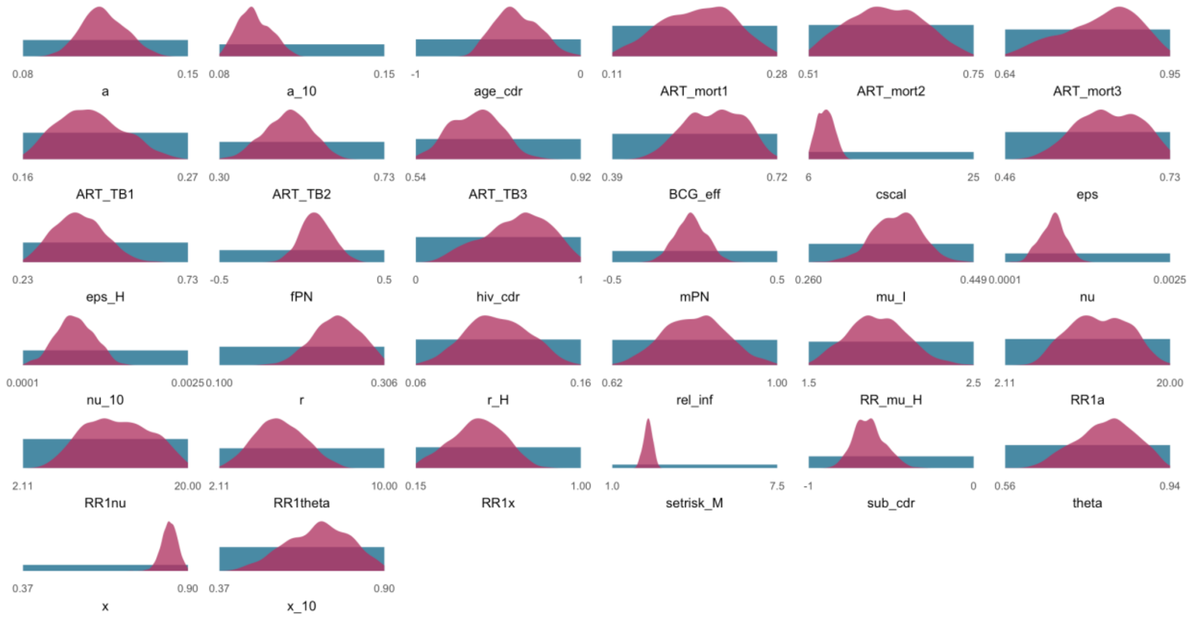

Supplementary Figure 7: The prior ranges and posterior distributions for each parameter for the 1000 selected parameter sets for Kenya

Supplementary Table 23: The prior ranges and posterior central estimates and 95% uncertainty intervals from the 1000 calibrated parameter sets for Kenya

Parameters	Symbols	Description	Priors	Posteriors
a	$\alpha_{a\geq15}$	Progression to disease, adults	0.080-0.15	0.114 [UI 0.098-0.133]
a_10	$\alpha_{a=0-14}$	Progression to disease, children	0.080-0.15	0.094 [UI 0.083-0.109]
age_cdr	$p_{a=0-14}$	Treatment coverage adjustment, children	-1.00-0.00	-0.40 [UI -0.62--0.13]
ART_mort1	$ART_{\mu_{\mathbf{sTB}_{t<6}}}$	ART mortality adjustment, 0-6 months	0.11-0.28	0.20 [UI 0.12-0.27]
ART_mort2	$ART_{\mu_{\mathbf{sTB}_{t=6-12}}}$	ART mortality adjustment, 6-12 months	0.51-0.75	0.62 [UI 0.53-0.72]
ART_mort3	$ART_{\mu_{\mathbf{sTB}_{t\geq12}}}$	ART mortality adjustment, 12+ months	0.64-0.95	0.83 [UI 0.68-0.93]
ART_TB1	$ART_{TB_{t<6}}$	ART risk reduction, 0-6 months	0.16-0.27	0.20 [UI 0.17-0.25]
ART_TB2	$ART_{TB_{t=6-12}}$	ART risk reduction, 6-12 months	0.30-0.73	0.48 [UI 0.36-0.59]
ART_TB3	$ART_{TB_{t\geq12}}$	ART risk reduction, 12+ months	0.54-0.92	0.68 [UI 0.57-0.78]
BCG_eff	$BCG_{eff_{a\geq15}}$	BCF efficacy	0.39-0.72	0.60 [UI 0.48-0.69]
cscal	$cscal$	Contact scaling parameter	6.00-25.00	7.92 [UI 6.25-9.82]
eps	$\epsilon_{h=HIV-}$	Regression through disease, HIV-	0.46-0.73	0.63 [UI 0.53-0.72]
eps_H	$\epsilon_{h=HIV+}$	Regression through disease, HIV+	0.23-0.73	0.40 [UI 0.27-0.55]
fPN	$p_{g=F}$	Treatment coverage adjustment, women	-0.50-0.50	0.086 [UI -0.089-0.284]
hiv_cdr	$p_{k=ART}$	Treatment coverage adjustment, ART	0.00-1.00	0.64 [UI 0.22-0.93]
mPN	$p_{g=M}$	Treatment coverage adjustment, men	-0.50-0.50	-0.026 [UI -0.205-0.155]
mu_I	$\mu_{\mathbf{sTB}}$	TB mortality from sympomatic disease	0.26-0.45	0.36 [UI 0.31-0.41]
nu	$\nu_{a\geq15}$	Progression from infection, adults	0.00010-0.0025	0.00081 [UI 0.00044-0.00112]
nu_10	$\nu_{a=0-14}$	Progression from infection, children	0.00010-0.0025	0.00085 [UI 0.00032-0.00136]
r	$r_{h=HIV-}$	Self cure, HIV-	0.10-0.31	0.25 [UI 0.19-0.30]
r_H	$r_{h=HIV+}$	Self cure, HIV+	0.060-0.16	0.108 [UI 0.075-0.144]
rel_inf	q	Relative infectiousness, asymptomatic disease	0.62-1.00	0.81 [UI 0.67-0.95]
RR_mu_H	RR_{μ_H}	Relative risk of mortality, HIV+	1.50-2.50	1.91 [UI 1.59-2.27]
RR1a	$RR1_{\alpha}$	Relative risk of progression to disease, HIV+	2.11-20.00	12.19 [UI 6.71-18.03]

Parameters	Symbols	Description	Priors	Posteriors
RR1nu	$RR_{1_{\nu}}$	Relative risk of progression from infection, HIV+	2.11-20.00	12.21 [UI 6.02-18.82]
RR1theta	$RR_{1_{\theta}}$	Relative risk of progression through disease, HIV+	2.11-10.00	4.96 [UI 2.83-7.36]
RR1x	$RR_{1_{x}}$	Relative risk of reinfection, HIV+	0.15-1.00	0.47 [UI 0.20-0.72]
setrisk_M	$RR_{sex_{g=M,a\geq 15}}$	Excess risk for men	1.00-7.50	2.42 [UI 2.06-2.74]
sub_cdr	$p_{d=\mathbf{aTB}}$	Treatment coverage adjustment, asymptomatic disease	-1.00-0.00	-0.64 [UI -0.80--0.44]
theta	θ	Progression through disease	0.56-0.94	0.80 [UI 0.66-0.91]
x	$x_{a\geq 15}$	Reinfection, adults	0.37-0.90	0.84 [UI 0.79-0.89]
x_10	$x_{a=0-14}$	Reinfection, children	0.37-0.90	0.69 [UI 0.48-0.87]

All selected parameter sets have model outputs that fit within the target ranges (Supplementary Figure 8. The prevalence estimates lie on the higher end of the calibration targets, whilst incidence and mortality lie on the lower ends. P:N ratios are relatively stable over time whilst the M:F prevalence ratio increases slightly over time.

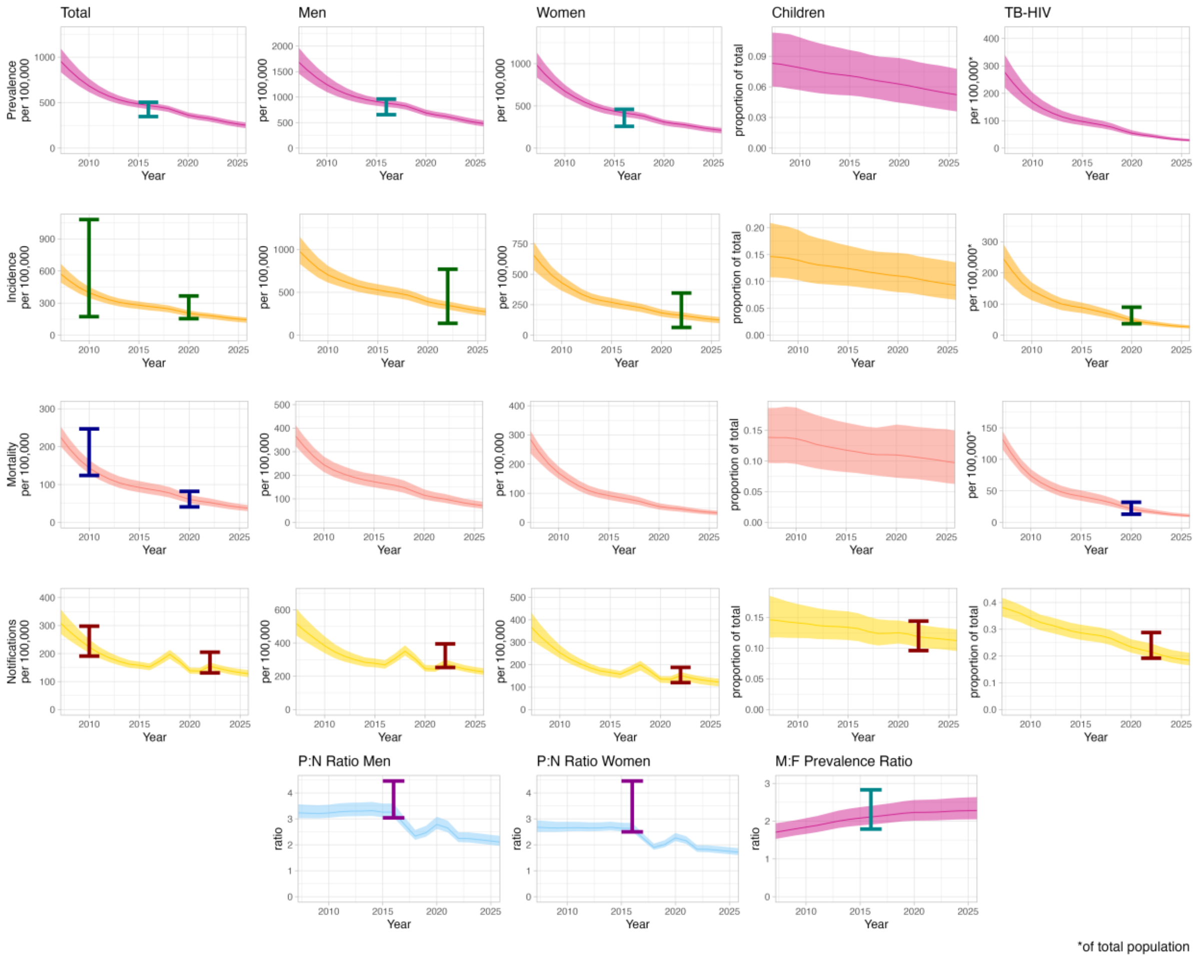

Supplementary Figure 8: Calibration plots for the Kenyan calibration targets using the 1000 selected parameters. The targets are the coloured error bars, the lines are the median estimates and the shaded regions are the 95% uncertainty intervals

5.2.1.2 Malawi

Over twelve waves, the calibration for Malawi reached 462/640 of the tested parameter sets hitting all 20 targets (Supplementary Figure 9. We used the emulators from wave 12 to find suitable parameter sets.

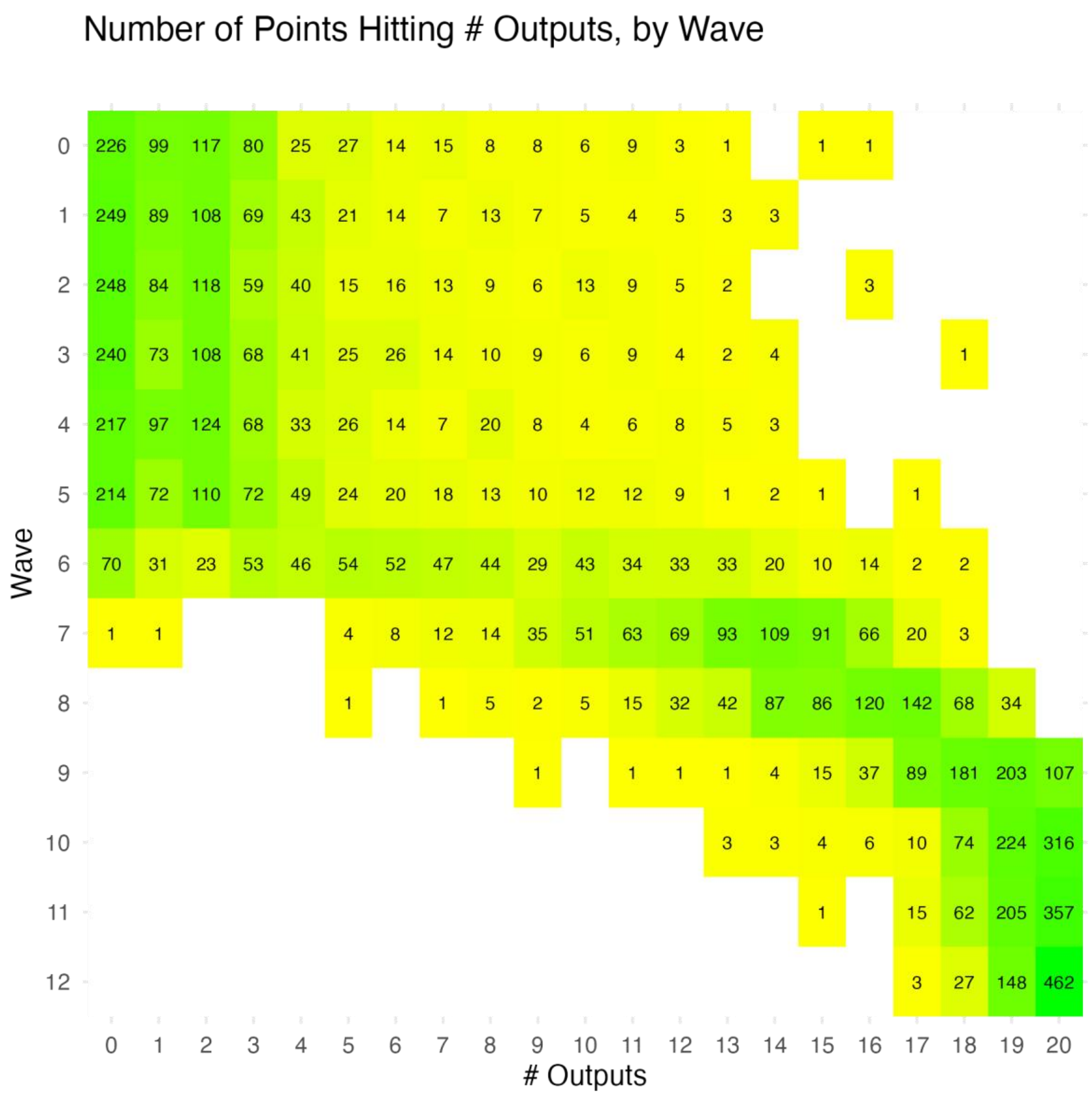

Supplementary Figure 9: The wave progression of the hmer process for the calibration of the Malawian model

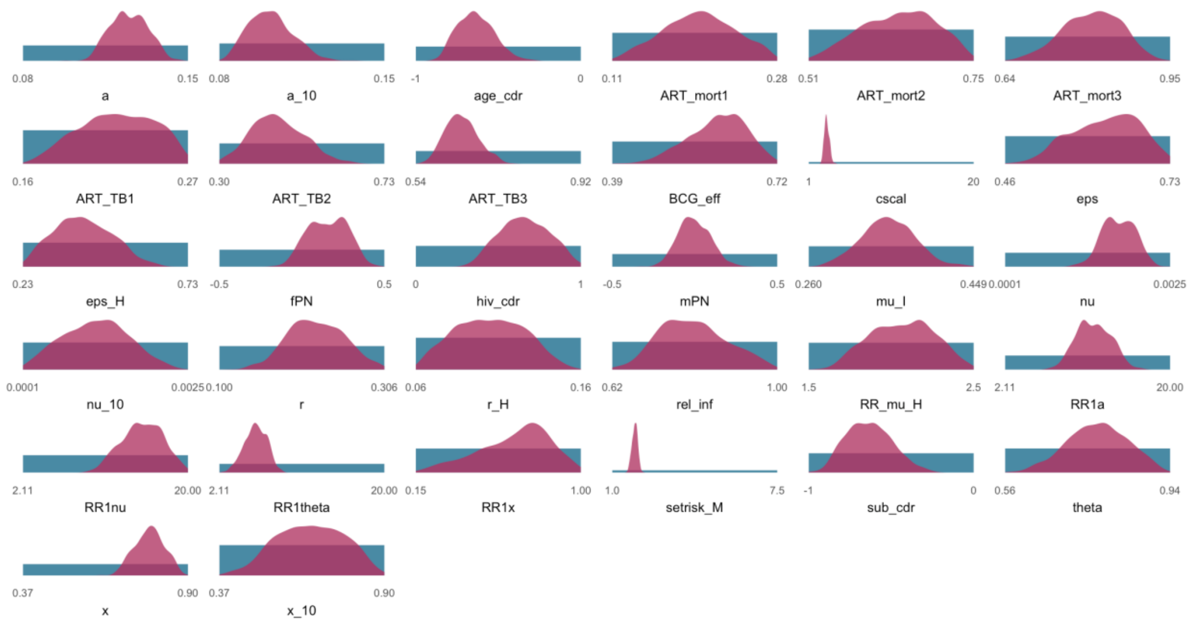

Supplementary Figure 10: The prior ranges and posterior distributions for each parameter for the 1000 selected parameter sets for Malawi

Supplementary Table 24: The prior ranges and posterior central estimates and 95% uncertainty intervals from the 1000 calibrated parameter sets for Malawi

Parameters	Symbols	Description	Priors	Posteriors
a	$\alpha_{a\geq15}$	Progression to disease, adults	0.080-0.15	0.12 [UI 0.11-0.14]
a_10	$\alpha_{a=0-14}$	Progression to disease, children	0.080-0.15	0.101 [UI 0.086-0.120]
age_cdr	$p_{a=0-14}$	Treatment coverage adjustment, children	-1.00-0.00	-0.64 [UI -0.82--0.41]
ART_mort1	$ART_{\mu_{\mathbf{sTB}_{t<6}}}$	ART mortality adjustment, 0-6 months	0.11-0.28	0.20 [UI 0.13-0.27]
ART_mort2	$ART_{\mu_{\mathbf{sTB}_{t=6-12}}}$	ART mortality adjustment, 6-12 months	0.51-0.75	0.64 [UI 0.54-0.73]
ART_mort3	$ART_{\mu_{\mathbf{sTB}_{t\geq12}}}$	ART mortality adjustment, 12+ months	0.64-0.95	0.81 [UI 0.69-0.90]
ART_TB1	$ART_{TB_{t<6}}$	ART risk reduction, 0-6 months	0.16-0.27	0.22 [UI 0.18-0.26]
ART_TB2	$ART_{TB_{t=6-12}}$	ART risk reduction, 6-12 months	0.30-0.73	0.45 [UI 0.33-0.60]
ART_TB3	$ART_{TB_{t\geq12}}$	ART risk reduction, 12+ months	0.54-0.92	0.64 [UI 0.58-0.72]
BCG_eff	$BCG_{eff_{a\geq15}}$	BCF efficacy	0.39-0.72	0.61 [UI 0.48-0.70]
cscal	$cscal$	Contact scaling parameter	1.00-20.00	3.06 [UI 2.59-3.67]
eps	$\epsilon_{h=HIV-}$	Regression through disease, HIV-	0.46-0.73	0.63 [UI 0.52-0.72]
eps_H	$\epsilon_{h=HIV+}$	Regression through disease, HIV+	0.23-0.73	0.41 [UI 0.26-0.59]
fPN	$p_{g=F}$	Treatment coverage adjustment, women	-0.50-0.50	0.158 [UI -0.062-0.362]
hiv_cdr	$p_{k=ART}$	Treatment coverage adjustment, ART	0.00-1.00	0.66 [UI 0.39-0.93]

Parameters	Symbols	Description	Priors	Posteriors
mPN	$p_{g=M}$	Treatment coverage adjustment, men	-0.50-0.50	-0.0077 [UI -0.1764-0.1975]
mu_I	$\mu_{\mathbf{sTB}}$	TB mortality from sympomatic disease	0.26-0.45	0.35 [UI 0.29-0.41]
nu	$\nu_{a\geq15}$	Progression from infection, adults	0.00010-0.0025	0.0018 [UI 0.0013-0.0022]
nu_10	$\nu_{a=0-14}$	Progression from infection, children	0.00010-0.0025	0.00116 [UI 0.00028-0.00207]
r	$r_{h=HIV-}$	Self cure, HIV-	0.10-0.31	0.22 [UI 0.15-0.29]
r_H	$r_{h=HIV+}$	Self cure, HIV+	0.060-0.16	0.105 [UI 0.068-0.144]
rel_inf	q	Relative infectiousness, asymptomatic disease	0.62-1.00	0.80 [UI 0.67-0.96]
RR_mu_H	RR_{μ_H}	Relative risk of mortality, HIV+	1.50-2.50	2.07 [UI 1.71-2.42]
RR1a	RR_{1_α}	Relative risk of progression to disease, HIV+	2.11-20.00	11.67 [UI 8.34-15.22]
RR1nu	RR_{1_ν}	Relative risk of progression from infection, HIV+	2.11-20.00	15.13 [UI 10.86-19.06]
RR1theta	RR_{1_θ}	Relative risk of progression through disease, HIV+	2.11-20.00	6.05 [UI 3.58-8.11]
RR1x	RR_{1_x}	Relative risk of reinfection, HIV+	0.15-1.00	0.69 [UI 0.26-0.95]
setrisk_M	$RR_{sex_{g=M,a\geq15}}$	Excess risk for men	1.00-7.50	1.89 [UI 1.67-2.09]
sub_cdr	$p_{d=\mathbf{aTB}}$	Treatment coverage adjustment, asymptomatic disease	-1.00-0.00	-0.64 [UI -0.87--0.35]
theta	θ	Progression through disease	0.56-0.94	0.77 [UI 0.63-0.90]
x	$x_{a\geq15}$	Reinfection, adults	0.37-0.90	0.78 [UI 0.69-0.86]
x_10	$x_{a=0-14}$	Reinfection, children	0.37-0.90	0.67 [UI 0.46-0.85]

All selected parameter sets have model outputs that fit within the target ranges (Supplementary Figure 11. The prevalence estimates lie in the centre of the calibration targets, whilst incidence and mortality lie on the lower ends. P:N ratios decrease over time whilst the M:F prevalence ratio is relatively stable.

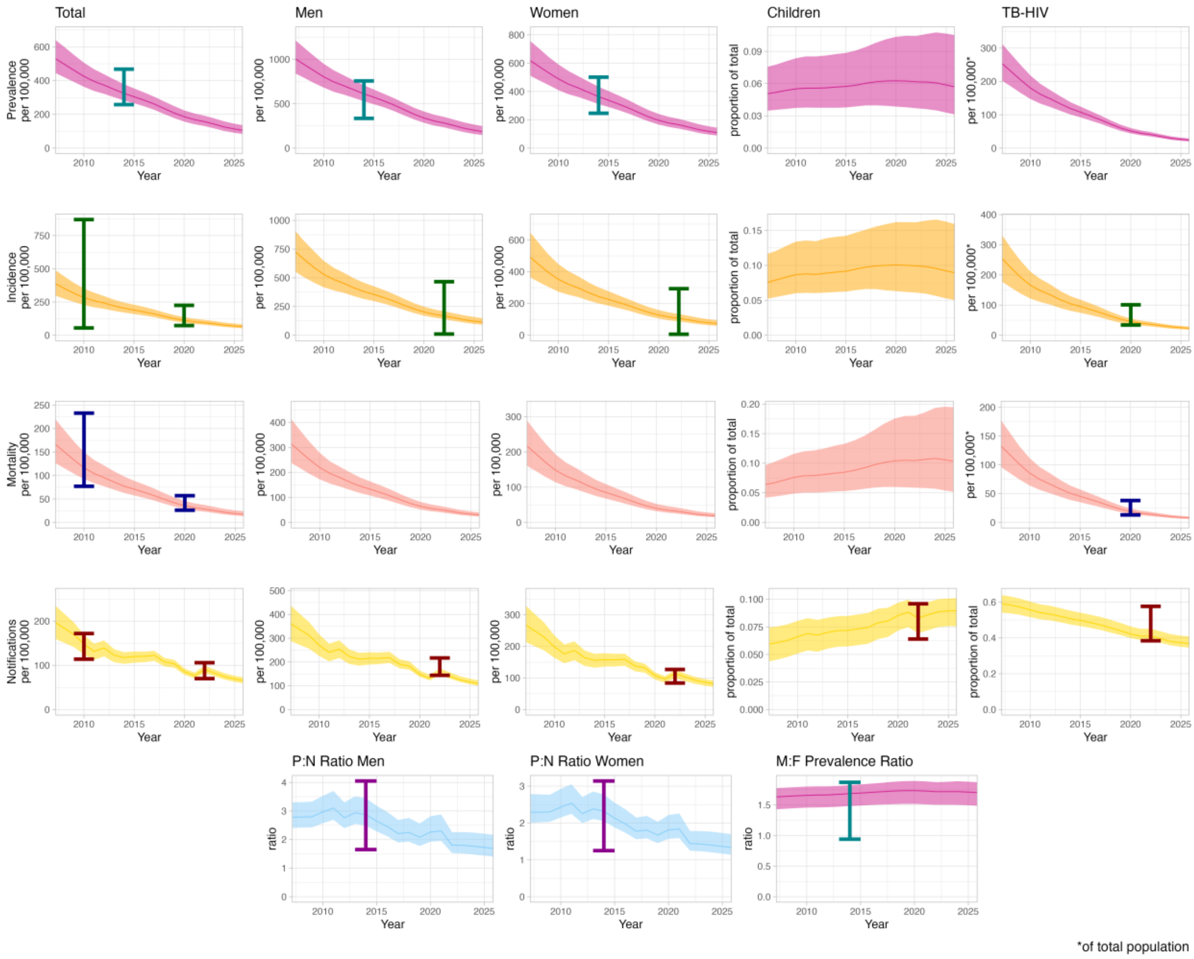

Supplementary Figure 11: Calibration plots for the Malawian calibration targets using the 1000 selected parameters. The targets are the coloured error bars, the lines are the median estimates and the shaded regions are the 95% uncertainty intervals

5.2.1.3 Nigeria

Over nineteen waves, the calibration for Nigeria reached 353/640 of the tested parameter sets hitting all 21 targets (Supplementary Figure 12. We used the emulators from wave 19 to find suitable parameter sets.

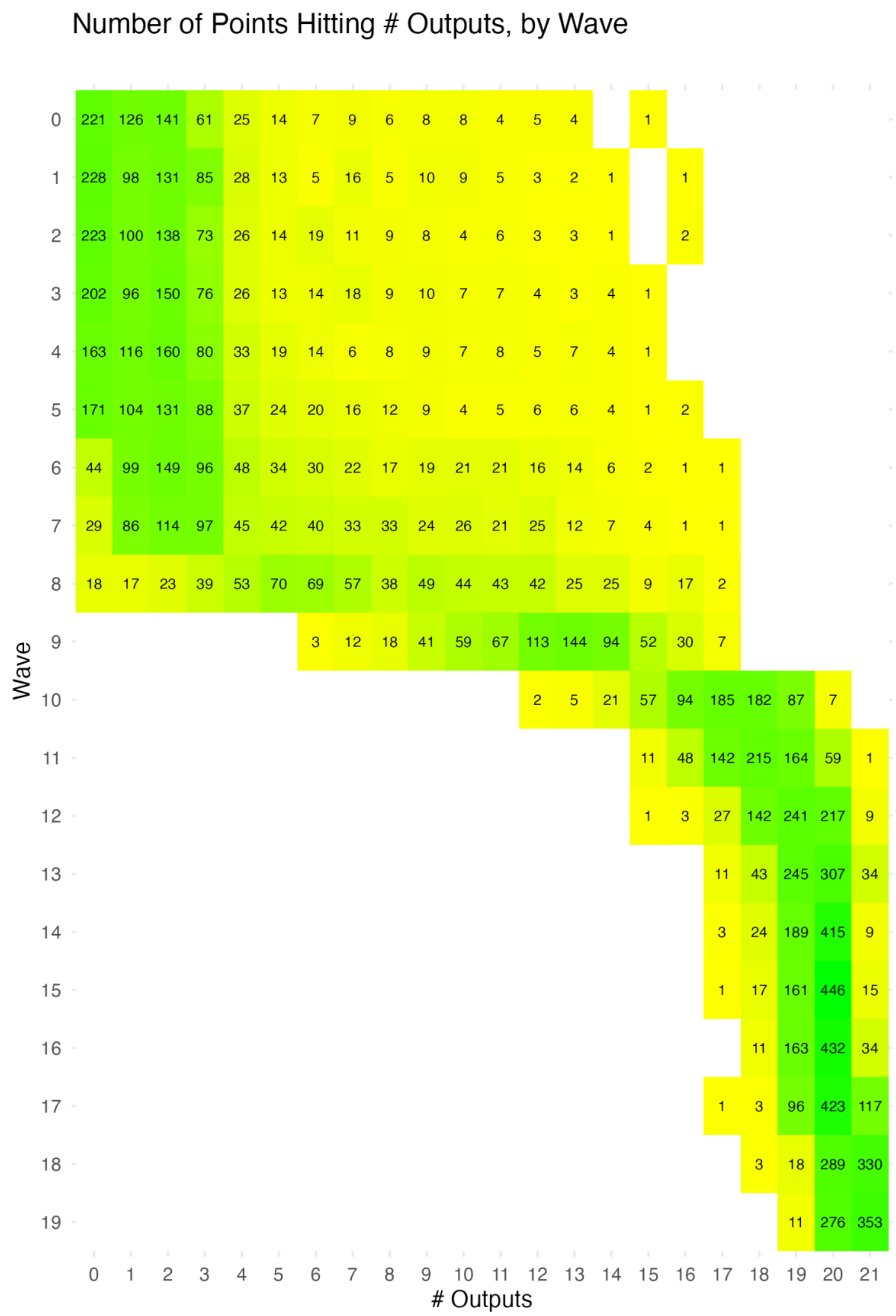

Supplementary Figure 12: The wave progression of the hmer process for the calibration of the Nigerian model

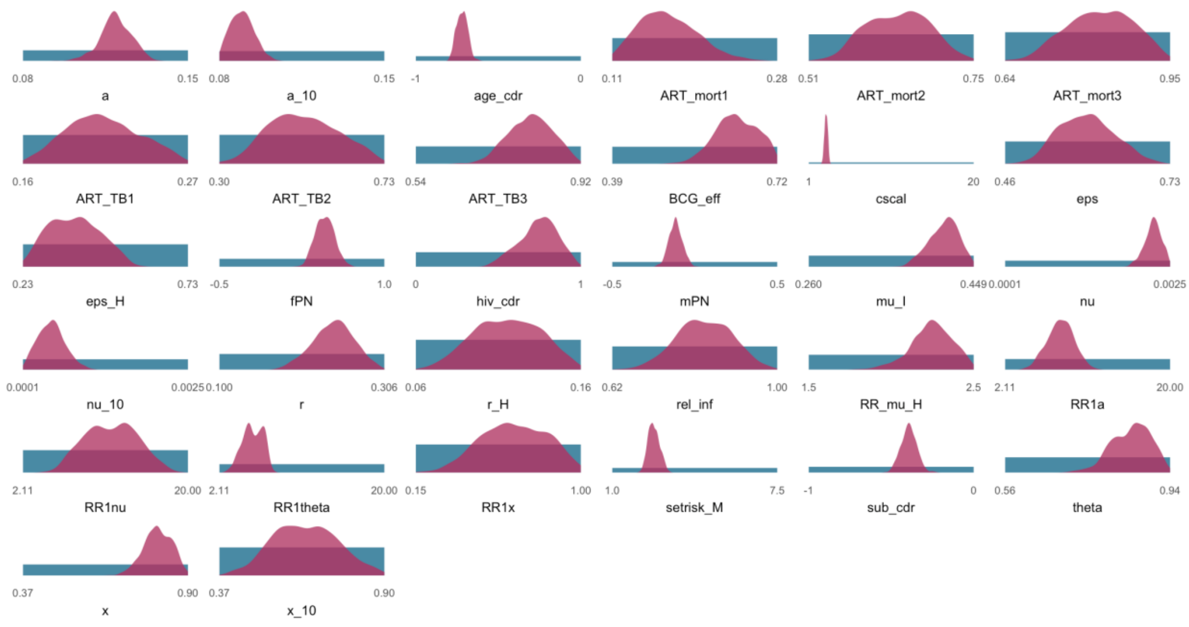

Supplementary Figure 13: The prior ranges and posterior distributions for each parameter for the 1000 selected parameter sets for Nigeria

Supplementary Table 25: The prior ranges and posterior central estimates and 95% uncertainty intervals from the 1000 calibrated parameter sets for Nigeroa

Parameters	Symbols	Description	Priors	Posteriors
a	$\alpha_{a\geq15}$	Progression to disease, adults	0.080-0.15	0.12 [UI 0.11-0.13]
a_10	$\alpha_{a=0-14}$	Progression to disease, children	0.080-0.15	0.090 [UI 0.081-0.100]
age_cdr	$p_{a=0-14}$	Treatment coverage adjustment, children	-1.00-0.00	-0.72 [UI -0.78--0.66]
ART_mort1	$ART_{\mu_{\mathbf{sTB}_{t<6}}}$	ART mortality adjustment, 0-6 months	0.11-0.28	0.16 [UI 0.12-0.23]
ART_mort2	$ART_{\mu_{\mathbf{sTB}_{t=6-12}}}$	ART mortality adjustment, 6-12 months	0.51-0.75	0.64 [UI 0.56-0.72]
ART_mort3	$ART_{\mu_{\mathbf{sTB}_{t\geq12}}}$	ART mortality adjustment, 12+ months	0.64-0.95	0.82 [UI 0.70-0.92]
ART_TB1	$ART_{TB_{t<6}}$	ART risk reduction, 0-6 months	0.16-0.27	0.21 [UI 0.17-0.26]
ART_TB2	$ART_{TB_{t=6-12}}$	ART risk reduction, 6-12 months	0.30-0.73	0.52 [UI 0.36-0.69]
ART_TB3	$ART_{TB_{t\geq12}}$	ART risk reduction, 12+ months	0.54-0.92	0.81 [UI 0.71-0.89]
BCG_eff	$BCG_{eff_{a\geq15}}$	BCF efficacy	0.39-0.72	0.64 [UI 0.56-0.71]
cscal	$cscal$	Contact scaling parameter	1.00-20.00	3.01 [UI 2.66-3.34]
eps	$\epsilon_{h=HIV-}$	Regression through disease, HIV-	0.46-0.73	0.59 [UI 0.51-0.68]
eps_H	$\epsilon_{h=HIV+}$	Regression through disease, HIV+	0.23-0.73	0.38 [UI 0.25-0.53]
fPN	$p_{g=F}$	Treatment coverage adjustment, women	-0.50-1.00	0.46 [UI 0.30-0.63]
hiv_cdr	$p_{k=ART}$	Treatment coverage adjustment, ART	0.00-1.00	0.75 [UI 0.51-0.94]

Parameters	Symbols	Description	Priors	Posteriors
mPN	$p_{g=M}$	Treatment coverage adjustment, men	-0.50-0.50	-0.116 [UI -0.193--0.037]
mu_I	$\mu_{\mathbf{sTB}}$	TB mortality from sympomatic disease	0.26-0.45	0.42 [UI 0.38-0.44]
nu	$\nu_{a\geq 15}$	Progression from infection, adults	0.00010-0.0025	0.0023 [UI 0.0020-0.0025]
nu_10	$\nu_{a=0-14}$	Progression from infection, children	0.00010-0.0025	0.00051 [UI 0.00018-0.00092]
r	$r_{h=HIV-}$	Self cure, HIV-	0.10-0.31	0.24 [UI 0.19-0.29]
r_H	$r_{h=HIV+}$	Self cure, HIV+	0.060-0.16	0.116 [UI 0.077-0.153]
rel_inf	q	Relative infectiousness, asymptomatic disease	0.62-1.00	0.83 [UI 0.71-0.95]
RR_mu_H	RR_{μ_H}	Relative risk of mortality, HIV+	1.50-2.50	2.25 [UI 1.97-2.46]
RR1a	RR_{1_α}	Relative risk of progression to disease, HIV+	2.11-20.00	8.14 [UI 5.35-10.82]
RR1nu	RR_{1_ν}	Relative risk of progression from infection, HIV+	2.11-20.00	11.73 [UI 6.65-16.98]
RR1theta	RR_{1_θ}	Relative risk of progression through disease, HIV+	2.11-20.00	5.69 [UI 3.61-7.45]
RR1x	RR_{1_x}	Relative risk of reinfection, HIV+	0.15-1.00	0.65 [UI 0.33-0.96]
setrisk_M	$RR_{sex_{g=M,a\geq 15}}$	Excess risk for men	1.00-7.50	2.62 [UI 2.24-3.09]
sub_cdr	$p_{d=\mathbf{aTB}}$	Treatment coverage adjustment, asymptomatic disease	-1.00-0.00	-0.39 [UI -0.48--0.30]
theta	θ	Progression through disease	0.56-0.94	0.86 [UI 0.76-0.92]
x	$x_{a\geq 15}$	Reinfection, adults	0.37-0.90	0.80 [UI 0.71-0.88]
x_10	$x_{a=0-14}$	Reinfection, children	0.37-0.90	0.64 [UI 0.45-0.85]

All selected parameter sets have model outputs that fit within the target ranges (Supplementary Figure 14). Overall prevalence estimates lie on the higher end of the calibration targets, whilst prevalence estimates for men and women are more central, along with incidence and mortality estimates. P:N ratios are relatively stable over time until 2020 when active case finding strategies were implemented, at which point they reduce.[68] At the point P:N ratios decrease, the M:F prevalence ratio starts increasing slightly.

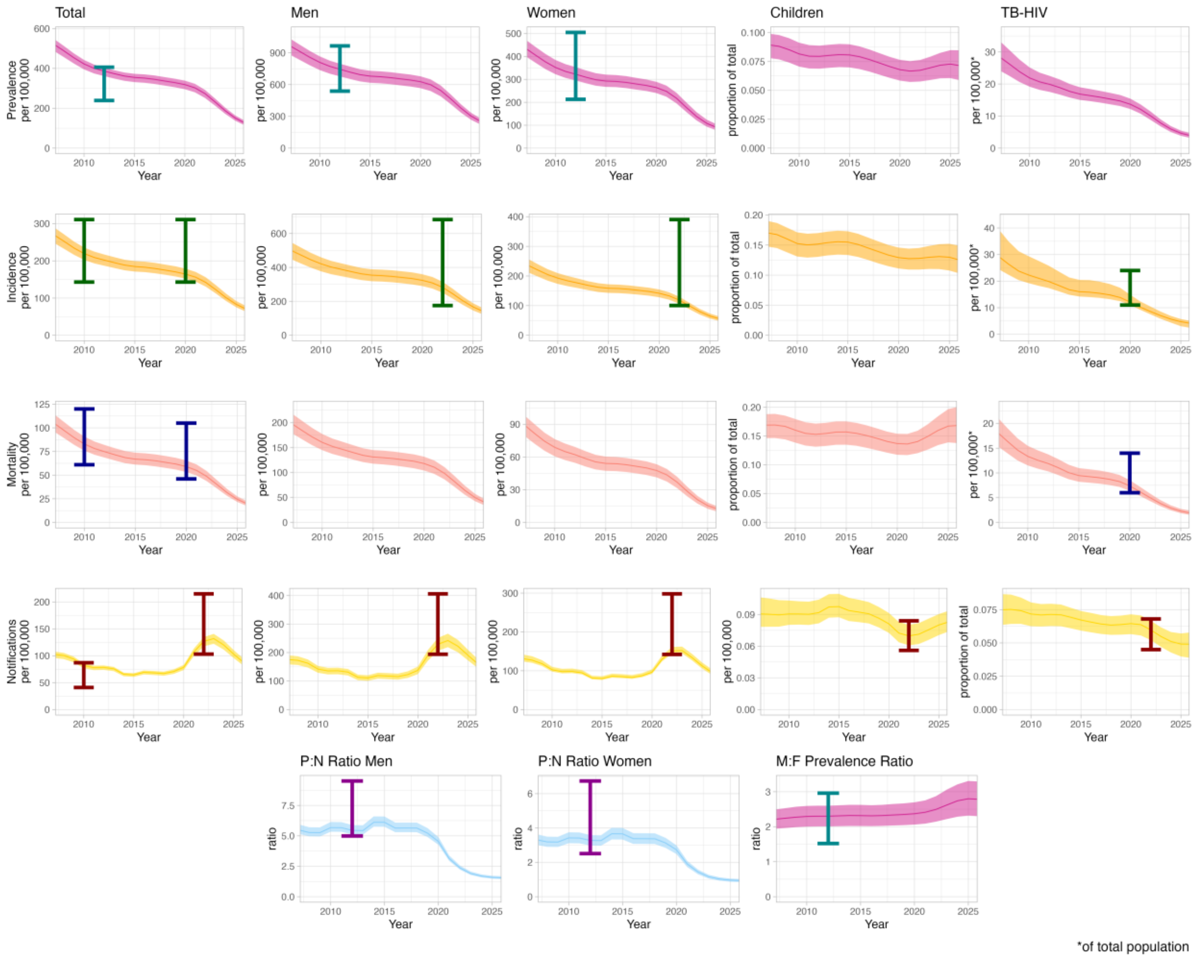

Supplementary Figure 14: Calibration plots for the Nigerian calibration targets using the 1000 selected parameters. The targets are the coloured error bars, the lines are the median estimates and the shaded regions are the 95% uncertainty intervals

5.2.1.4 Uganda

Over thirteen waves, the calibration for Uganda reached a maximum of 306/700, but running further waves reduced this slightly and we used the emulators from wave 13 to fund suitable parameter sets, which had 262/700 of the tested parameter sets hitting all 25 targets (Supplementary Figure 15.

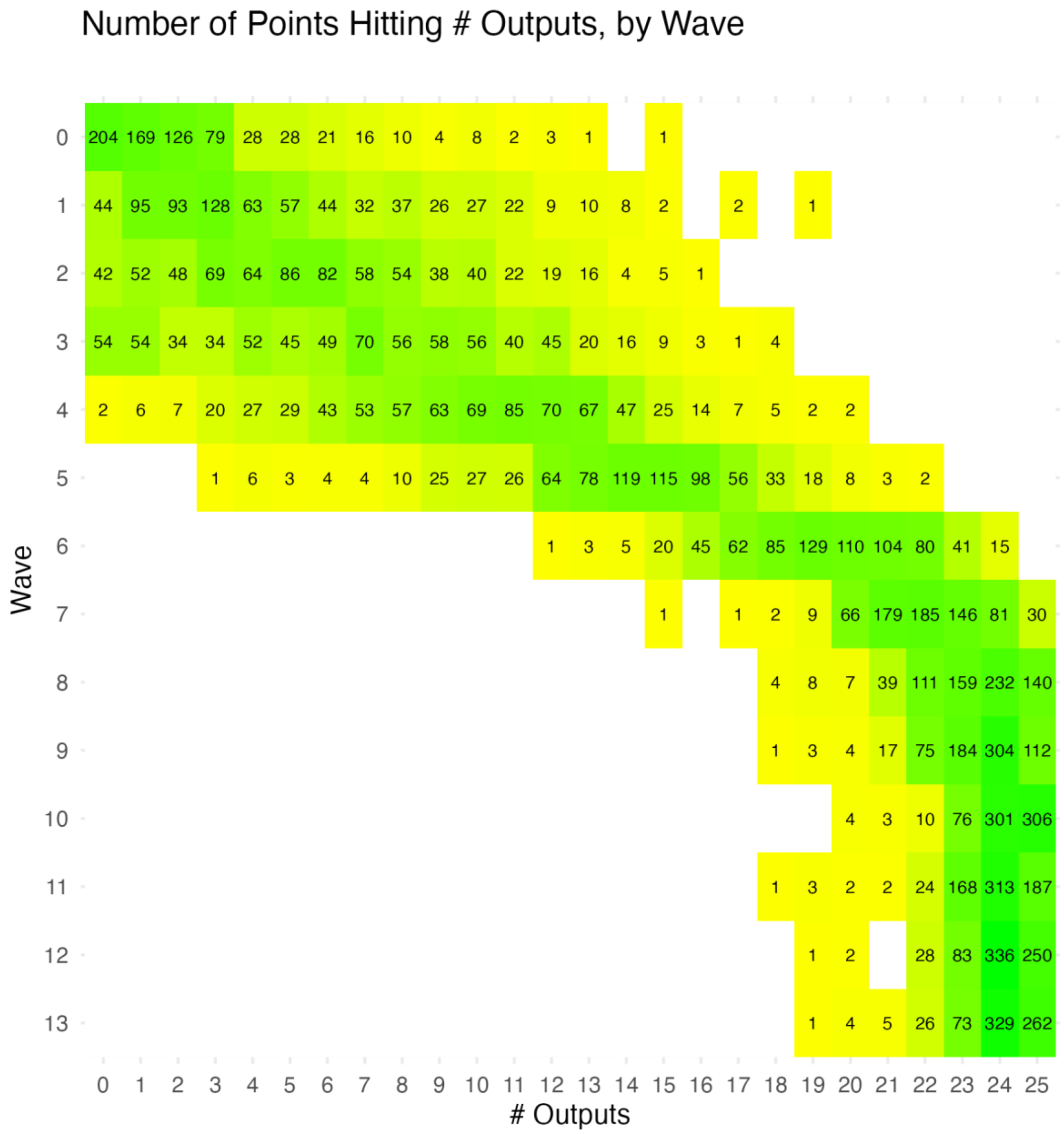

Supplementary Figure 15: The wave progression of the hmer process for the calibration of the Ugandan model

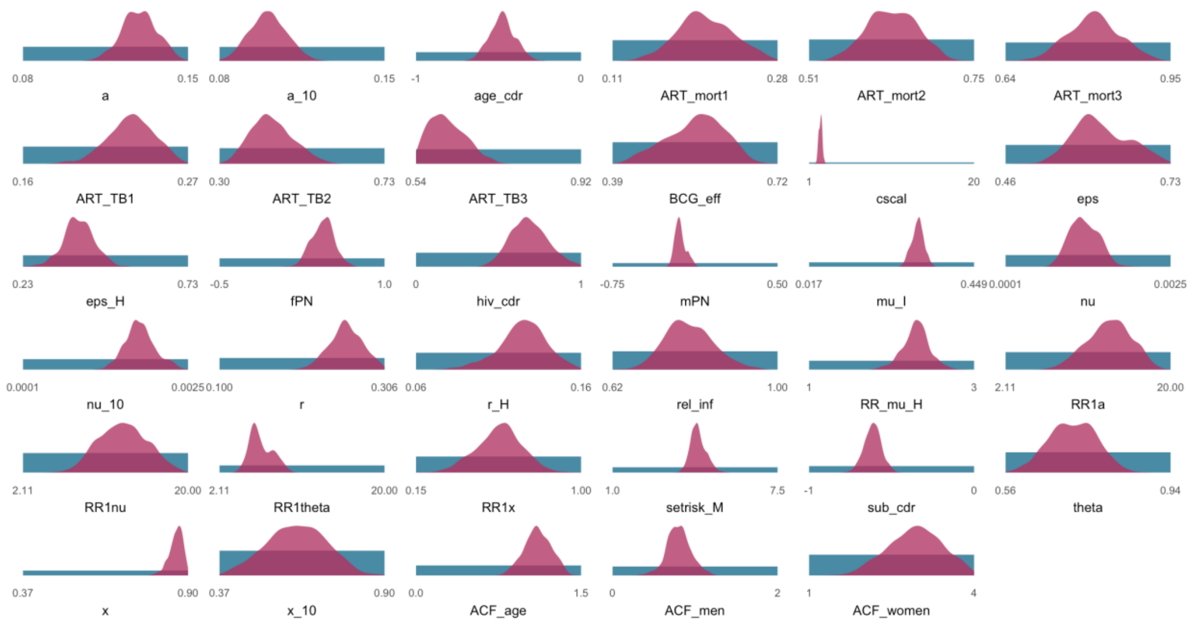

Supplementary Figure 16: The prior ranges and posterior distributions for each parameter for the 1000 selected parameter sets for Uganda

Supplementary Table 26: The prior ranges and posterior central estimates and 95% uncertainty intervals from the 1000 calibrated parameter sets for Uganda

Parameters	Symbols	Description	Priors	Posteriors
a	$\alpha_{a\geq15}$	Progression to disease, adults	0.080-0.15	0.13 [UI 0.11-0.14]
a_10	$\alpha_{a=0-14}$	Progression to disease, children	0.080-0.15	0.100 [UI 0.086-0.116]
age_cdr	$p_{a=0-14}$	Treatment coverage adjustment, children	-1.00-0.00	-0.48 [UI -0.62--0.33]
ART_mort1	$ART_{\mu_{\mathbf{sTB}_{t<6}}}$	ART mortality adjustment, 0-6 months	0.11-0.28	0.20 [UI 0.15-0.26]
ART_mort2	$ART_{\mu_{\mathbf{sTB}_{t=6-12}}}$	ART mortality adjustment, 6-12 months	0.51-0.75	0.63 [UI 0.56-0.70]
ART_mort3	$ART_{\mu_{\mathbf{sTB}_{t\geq12}}}$	ART mortality adjustment, 12+ months	0.64-0.95	0.81 [UI 0.71-0.90]
ART_TB1	$ART_{TB_{t<6}}$	ART risk reduction, 0-6 months	0.16-0.27	0.23 [UI 0.20-0.26]
ART_TB2	$ART_{TB_{t=6-12}}$	ART risk reduction, 6-12 months	0.30-0.73	0.43 [UI 0.34-0.55]
ART_TB3	$ART_{TB_{t\geq12}}$	ART risk reduction, 12+ months	0.54-0.92	0.61 [UI 0.55-0.70]
BCG_eff	$BCG_{eff_{a\geq15}}$	BCF efficacy	0.39-0.72	0.56 [UI 0.44-0.65]
cscal	$cscal$	Contact scaling parameter	1.00-20.00	2.33 [UI 1.94-2.70]
eps	$\epsilon_{h=HIV-}$	Regression through disease, HIV-	0.46-0.73	0.61 [UI 0.54-0.71]
eps_H	$\epsilon_{h=HIV+}$	Regression through disease, HIV+	0.23-0.73	0.40 [UI 0.31-0.49]
fPN	$p_{g=F}$	Treatment coverage adjustment, women	-0.50-1.00	0.44 [UI 0.25-0.63]
hiv_cdr	$p_{k=ART}$	Treatment coverage adjustment, ART	0.00-1.00	0.68 [UI 0.49-0.92]

Parameters	Symbols	Description	Priors	Posteriors
mPN	$p_{g=M}$	Treatment coverage adjustment, men	-0.75-0.50	-0.24 [UI -0.31--0.14]
mu_I	$\mu_{\mathbf{sTB}}$	TB mortality from sympomatic disease	0.017-0.45	0.30 [UI 0.27-0.33]
nu	$\nu_{a\geq15}$	Progression from infection, adults	0.00010-0.0025	0.00122 [UI 0.00087-0.00164]
nu_10	$\nu_{a=0-14}$	Progression from infection, children	0.00010-0.0025	0.0018 [UI 0.0014-0.0023]
r	$r_{h=HIV-}$	Self cure, HIV-	0.10-0.31	0.26 [UI 0.22-0.30]
r_H	$r_{h=HIV+}$	Self cure, HIV+	0.060-0.16	0.125 [UI 0.093-0.151]
rel_inf	q	Relative infectiousness, asymptomatic disease	0.62-1.00	0.79 [UI 0.70-0.91]
RR_mu_H	RR_{μ_H}	Relative risk of mortality, HIV+	1.00-3.00	2.30 [UI 1.95-2.63]
RR1a	RR_{1_α}	Relative risk of progression to disease, HIV+	2.11-20.00	13.48 [UI 8.60-17.65]
RR1nu	RR_{1_ν}	Relative risk of progression from infection, HIV+	2.11-20.00	12.91 [UI 8.17-17.87]
RR1theta	RR_{1_θ}	Relative risk of progression through disease, HIV+	2.11-20.00	6.31 [UI 4.67-9.17]
RR1x	RR_{1_x}	Relative risk of reinfection, HIV+	0.15-1.00	0.58 [UI 0.34-0.80]
setrisk_M	$RR_{sex_{g=M,a\geq15}}$	Excess risk for men	1.00-7.50	4.34 [UI 3.85-5.04]
sub_cdr	$p_{d=\mathbf{aTB}}$	Treatment coverage adjustment, asymptomatic disease	-1.00-0.00	-0.61 [UI -0.72--0.51]
theta	θ	Progression through disease	0.56-0.94	0.72 [UI 0.61-0.81]
x	$x_{a\geq15}$	Reinfection, adults	0.37-0.90	0.86 [UI 0.81-0.89]
x_10	$x_{a=0-14}$	Reinfection, children	0.37-0.90	0.62 [UI 0.43-0.79]
ACF_age	$f_{a=0-14,g}$	Impact of CAST, children	0.00-1.50	1.10 [UI 0.86-1.33]
ACF_men	$f_{a\geq15,g=M}$	Impact of CAST, men	0.00-2.00	0.80 [UI 0.50-1.10]
ACF_women	$f_{a\geq15,g=F}$	Impact of CAST, women	1.00-4.00	2.95 [UI 1.97-3.80]

All selected parameter sets have model outputs that fit within the target ranges (Supplementary Figure 17). Overall prevalence estimates lie on the higher end of the calibration targets, whilst prevalence estimates for men and women are more central, along with incidence and mortality estimates. P:N ratios start to reduce from 2015 onwards to 2022 after which they become more stationary, whilst the M:F prevalence ratio increases slightly over time, until 2023 when it reduces slightly.

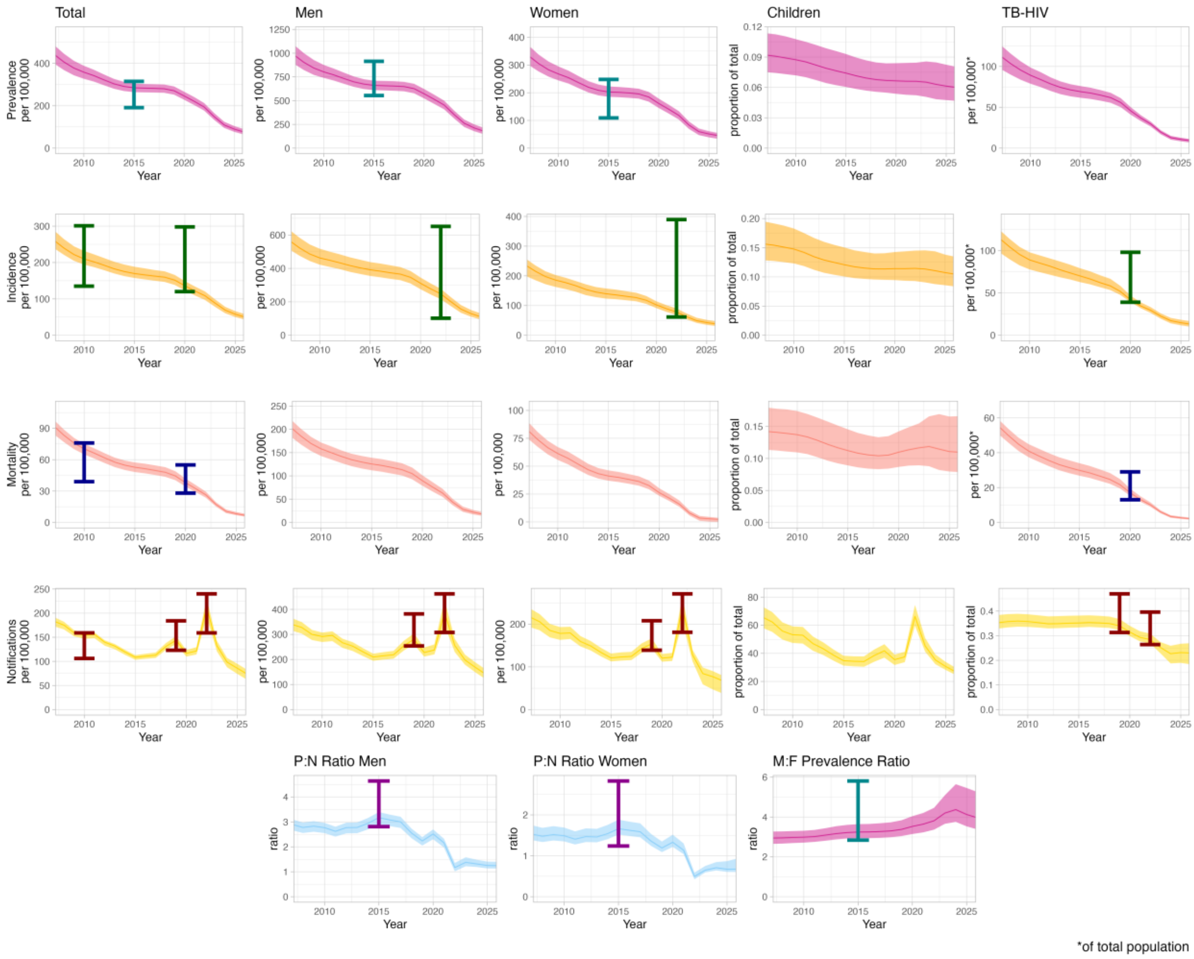

Supplementary Figure 17: Calibration plots for the Ugandan calibration targets using the 1000 selected parameters. The targets are the coloured error bars, the lines are the median estimates and the shaded regions are the 95% uncertainty intervals

6 Implementation of interventions

The purpose of this model is to find the potential impact of implementing interventions specifically focussed on men. We have approached this with three different methods.

6.1 Adjusting access to care

We know that men often do not access care at the same rate as women, and have included this in our assumptions around access to care in Section 3.6.1.1. We have assumed that it is possible to increase men's access to care to the same level achieved in women. Within the model, this is represented as increasing $p_{g=M}$ to equal $p_{g=F}$. Acknowledging that this change would not be possible overnight, we introduced a time dependent parameter to drive the change, δ_p, that varies from 0 before 2025 to 1 after 2030. Over 5 years, from 2025 to 2030, the difference between the two parameters is decreased with an approximately S-shaped curve, as seen in Supplementary Figure 18. The curve is derived from the equation $3x^2 - 2x^3$ with the values for each year taken from the evaluation of the curve at $\{0.2,0.4,0.6,0.8\}$. Mathematically, this changes the formula for access to care to be

$$p_{a,d,h,k} = \left(\begin{cases} 1 + p_a & \text{if } a < 15 \\ 1 + p_k & \text{if on ART} \\ (1 + p_d) \times (1 + p_{g=F}) & \text{for women} \\ (1 + p_d) \times (1 + p_{g=M}) & \text{for men if } t < 2025 \\ (1 + p_d) \times \left(1 + \left(p_{g=M} + \left(p_{g=F} - p_{g=M}\right) \times \delta_p\right)\right) & \text{for men if } t \geq 2025 \end{cases} \right)$$

Beyond 2030, the formula for men and women (not on ART) is identical ($(1 + p_d) \times (1 + p_{g=F})$) as when $\delta_p = 1$, the expression $p_{g=M} + \left(p_{g=F} - p_{g=M}\right) \times \delta_p$ simplifies to $p_{g=F}$.

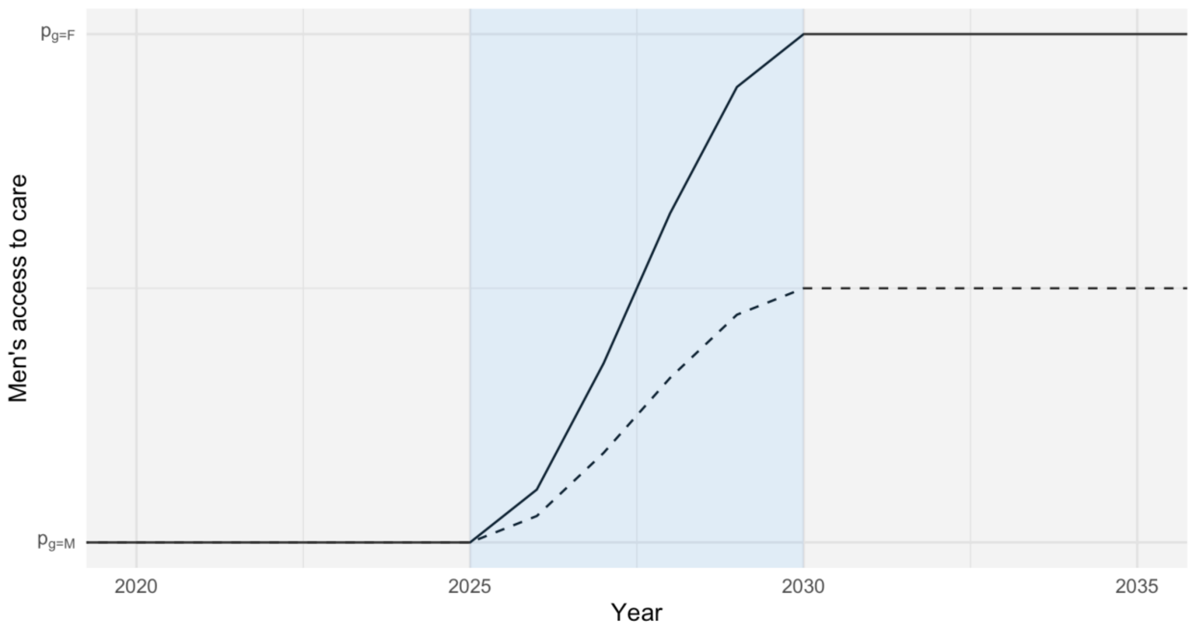

Supplementary Figure 18: The improvement of men's access to care over time, compared to women's access to care, the supplementary analysis of closing the gap by 50% is shown by a dashed line

6.2 Reducing excess risk

Men also have an excess risk of infection, ascribed to social and structural determinants, increased contact with men compared to women and children, and underlying biological factors. It will not be possible to eliminate the excess risk in men, however, it may be possible to reduce it through interventions focussed on the social and structural determinants that particularly differentially impact men. These could include smoking and drinking cessation interventions, social protection measures, or nutritional support. We are not stating which intervention, or combination of interventions, is being implemented, but instead looking at the broader statement of what is the impact if these interventions can reduce the risk by a given percentage. As with improving the access to care in Section 6.1, reducing the excess risk is achieved by introducing another time-dependent variable, $\delta_{RR_{sex}}$, which also uses an approximate S-shaped curve. The same equation for the curve is used as in Section 6.1, adapted to decrease from 1 to 0 and incorporate fractional decreases rather, $1 - (3x^2 - 2x^3) \times w$, where w is the proportion of risk reduction, with the values for each year taken from the evaluation of the curve at $\{0.2,0.4,0.6,0.8\}$. With this implementation, $RR_{sex_{g=M,a\geq15}}$ becomes a time-dependent parameter calculated as:

$$RR_{sex_{g=M,a\geq15,t}} = 1 + \left(RR_{sex_{g=M,a\geq15}} - 1\right) \times \delta_{RR_{sex}}$$

Our main analyses have been conducted assuming a 50% reduction in risk is achievable, but sensitivity analyses have been conducted with 25% and 75% reductions.

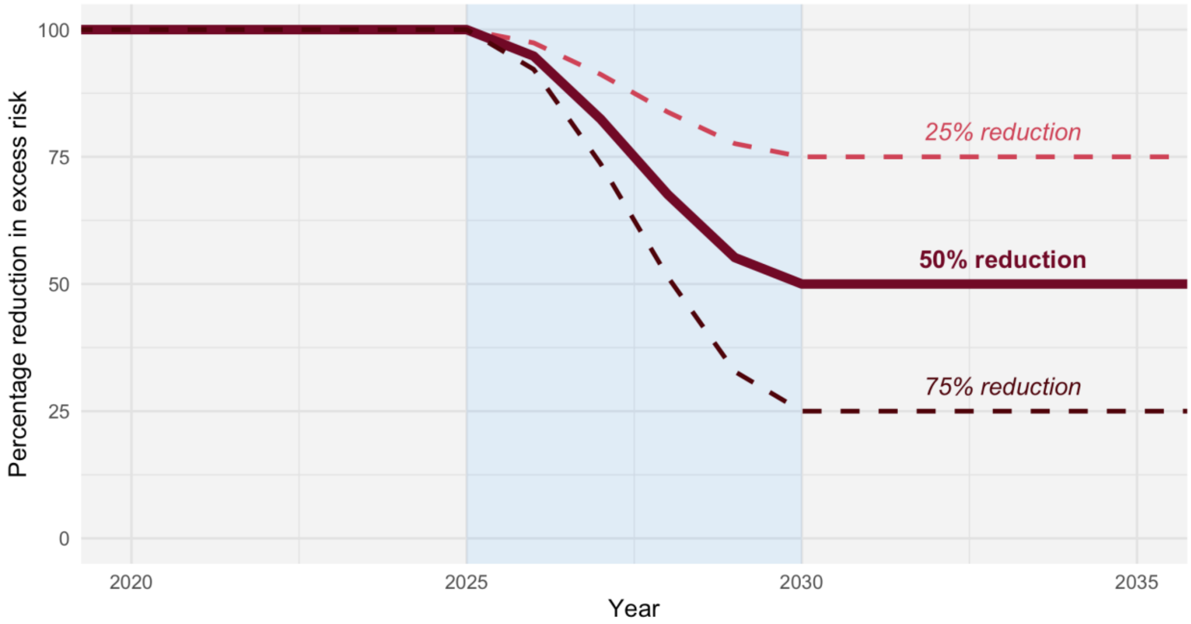

Supplementary Figure 19: The reduction in men's excess risk over time

7 Supplementary results

7.1 Partial Rank Correlation Coefficients

The partial rank correlation coefficients highlights the dependence of the outcomes on each of the parameter values within the model. Common thresholds for PRCC are ± 0.3 for weak correlations, ± 0.5 for moderate correlations, and ± 0.7 for strong correlations. Below we show plots that represent this calculation for each of the parameters, for each country, for the two main outcomes of interest: the percentage reduction in incidence in 2035 and the percentage reduction in notifications in 2035, each for the three strategies highlighted in the main analysis. We have included dashed lines at each of the three thresholds to aid interpretation of the results. These results were calculated using the epiR package.[69]

For reduction of incidence (Supplementary Figure 20) most parameters have limited to no correlation with the outcome. Across all four countries, the adjustment to treatment coverage for women is strongly positively correlated with the reduction in incidence when increasing men's treatment coverage. This is because the increase of men's treatment coverage is to meet women's treatment coverage. Similarly, the adjustment for treatment coverage for men is strongly negatively correlated as the higher men's treatment coverage is, and the closer it is to women's treatment coverage, the less space there is for improvement within this strategy. Men's excess risk was similarly highly correlated to reduction in incidence under the risk reduction strategy, as the more risk that can be reduced, the greater the reduction in incidence. Rate of progression to disease (ν) and related parameters were also moderately negatively correlated for the risk reduction strategy, as was the adjustment for treatment coverage for asymptomatic disease in all countries other than Kenya. The reduction in notifications (Supplementary Figure 21) showed very similar patterns.

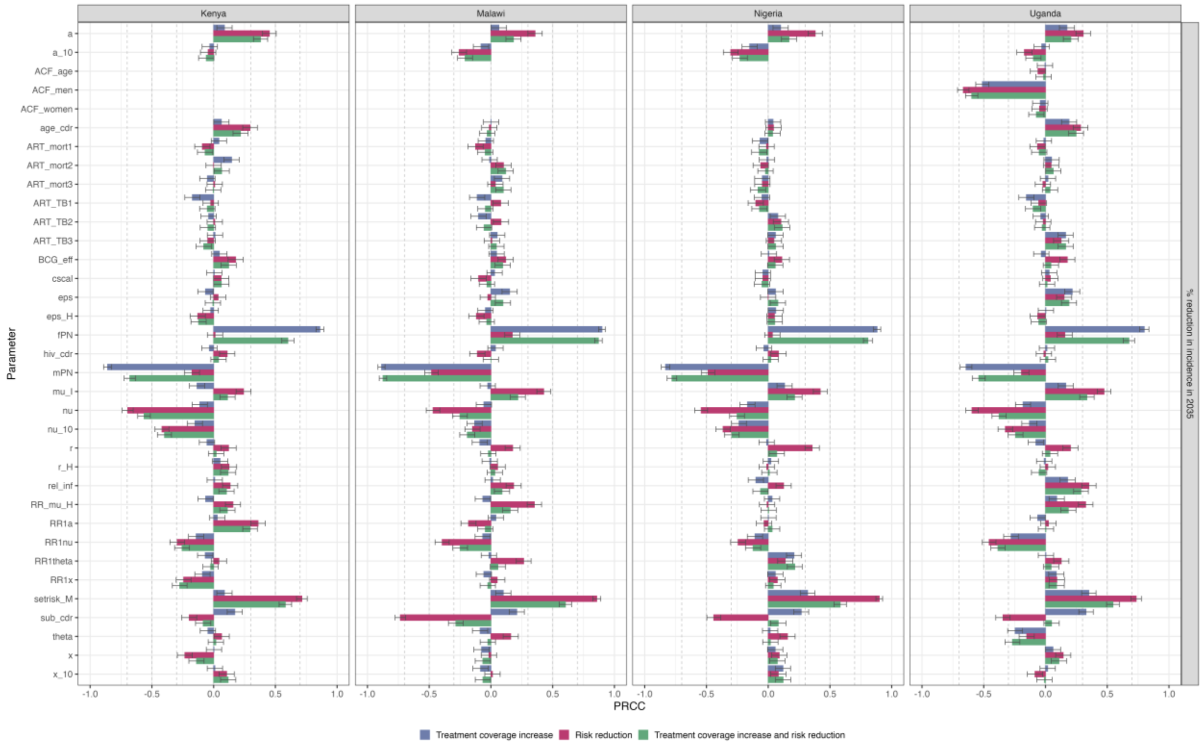

Supplementary Figure 20: PRCC for all the countries for all the parameters for the reduction in incidence

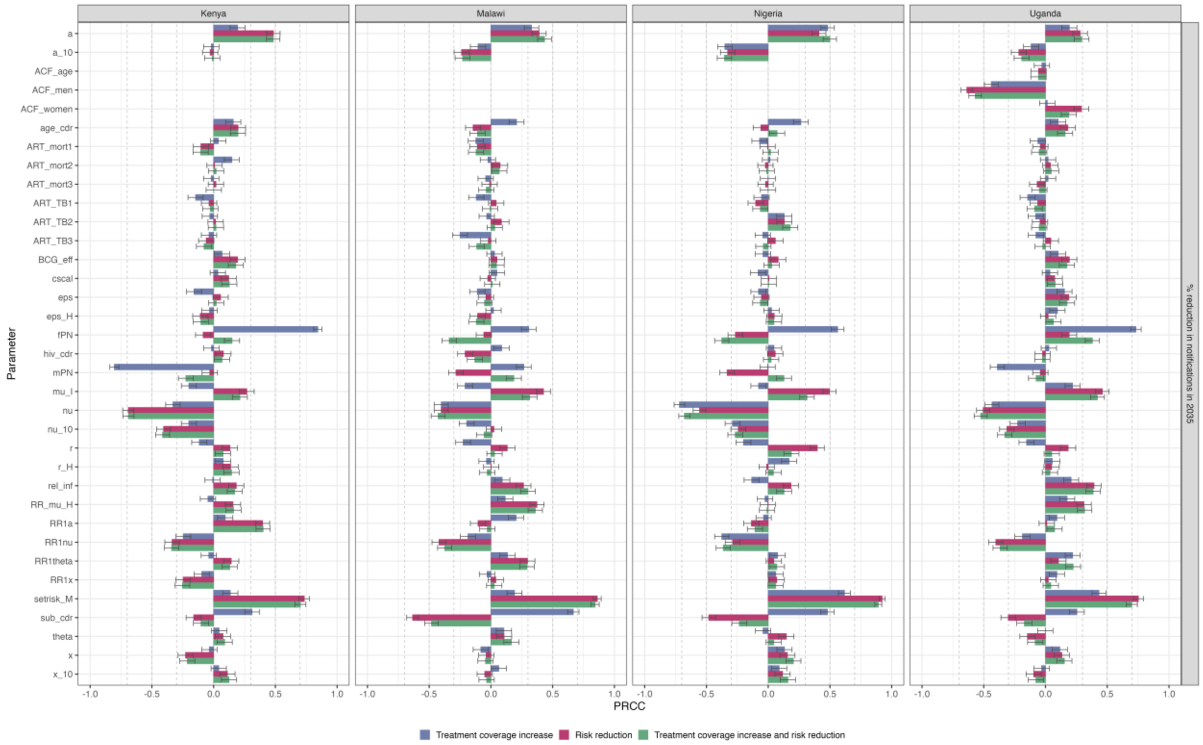

Supplementary Figure 21: PRCC for all the countries for all the parameters for the reduction in notifications

7.2 Impact of interventions on mortality and prevalence compared to incidence

Each strategy generally has a slightly larger impact on population-level mortality than it does on either prevalence or incidence, which are largely the same. However, the differences are not statistically significant as can be seen in Supplementary Figure 22 and.

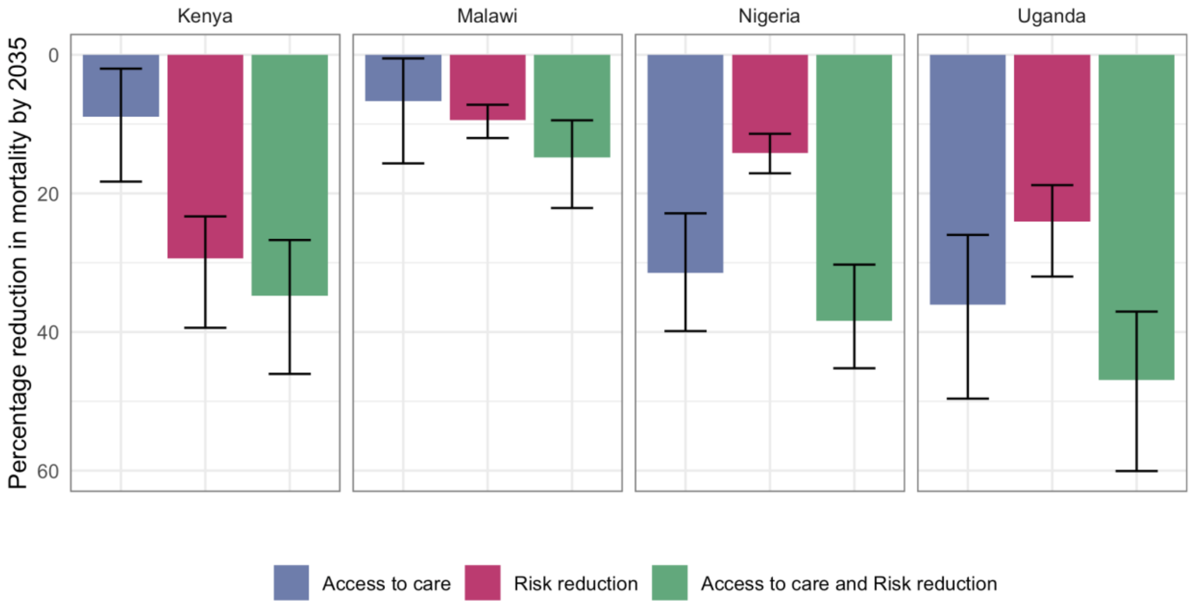

Supplementary Figure 22: The impact of each strategy on mortality at the population level

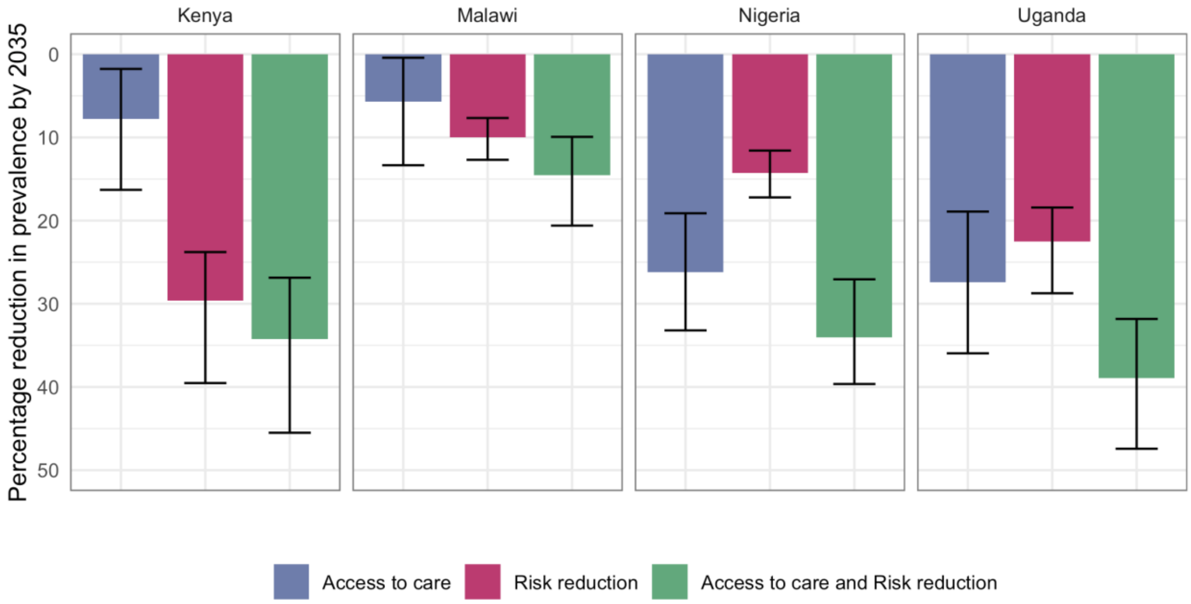

Supplementary Figure 23: The impact of each strategy on prevalence at the population level

7.3 Impact out to 2050

7.3.1 Incidence

Impact of all strategies increases by 2050 as compared to 2035, with the impact of reduction in risk increasing more than the impact of access to care as can be seen in Supplementary Figure 24, likely because reduction in risk takes a longer time to take effect.

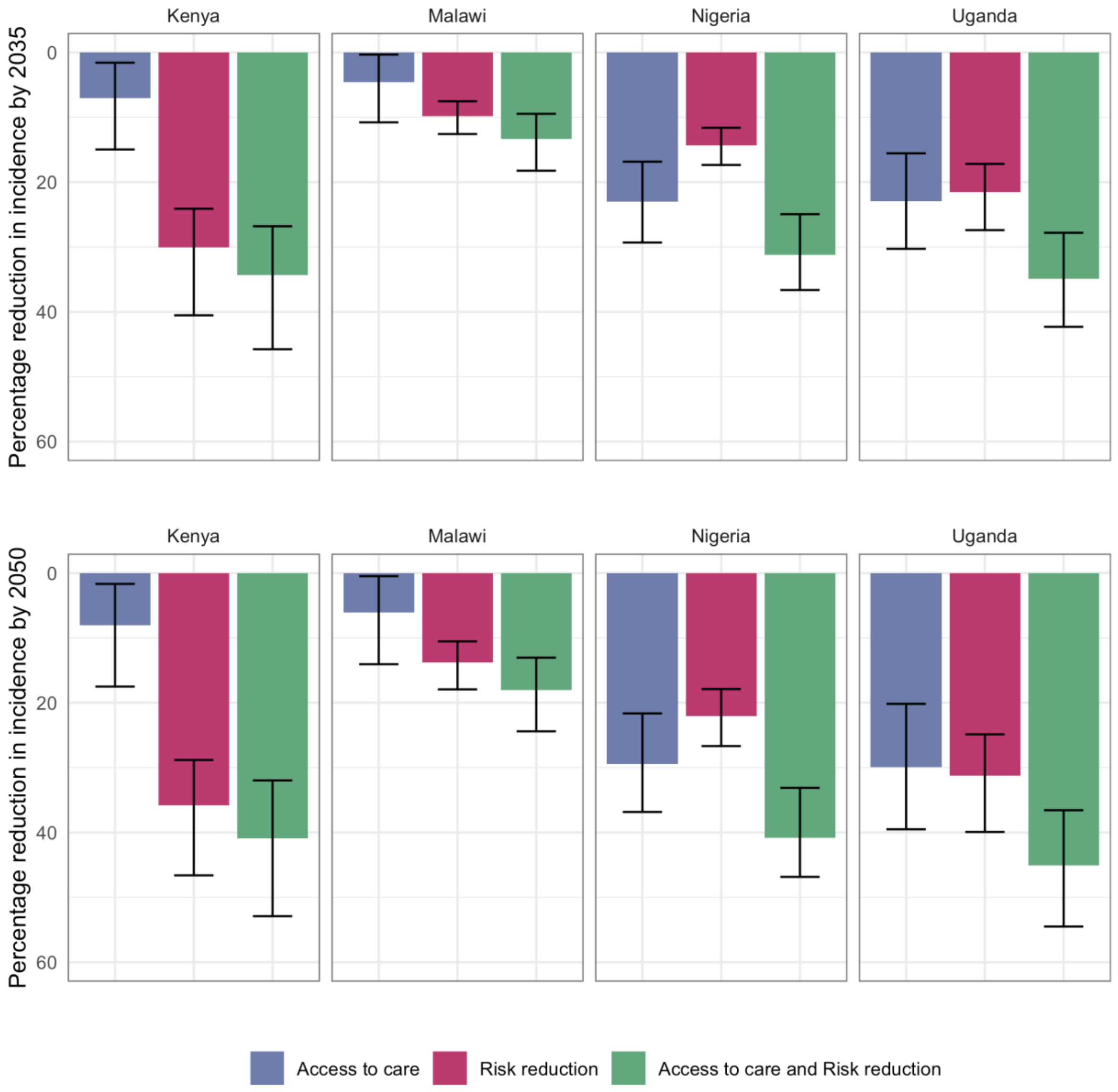

Supplementary Figure 24: The reduction in population-level incidence across the three strategies and across three different risk reduction levels

7.3.2 Notifications

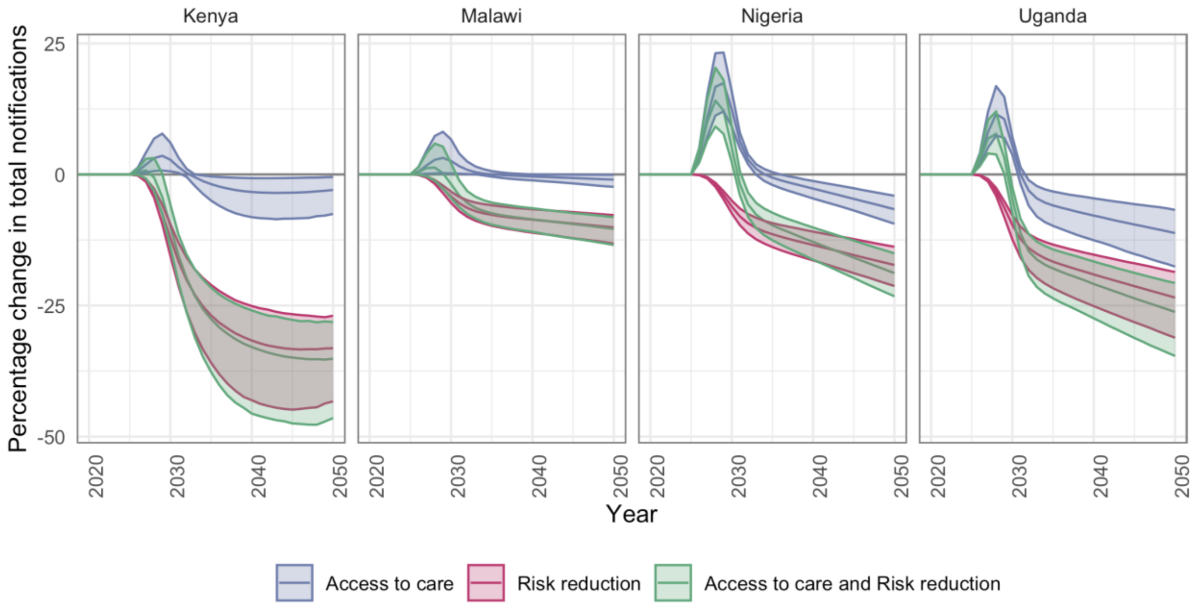

Supplementary Figure 25: The change in notifications for each scenario from 2020 to 2050 after the introduction of the three strategies between 2025 and 2030

7.4 Impact of only halving the gap in access to care between men and women

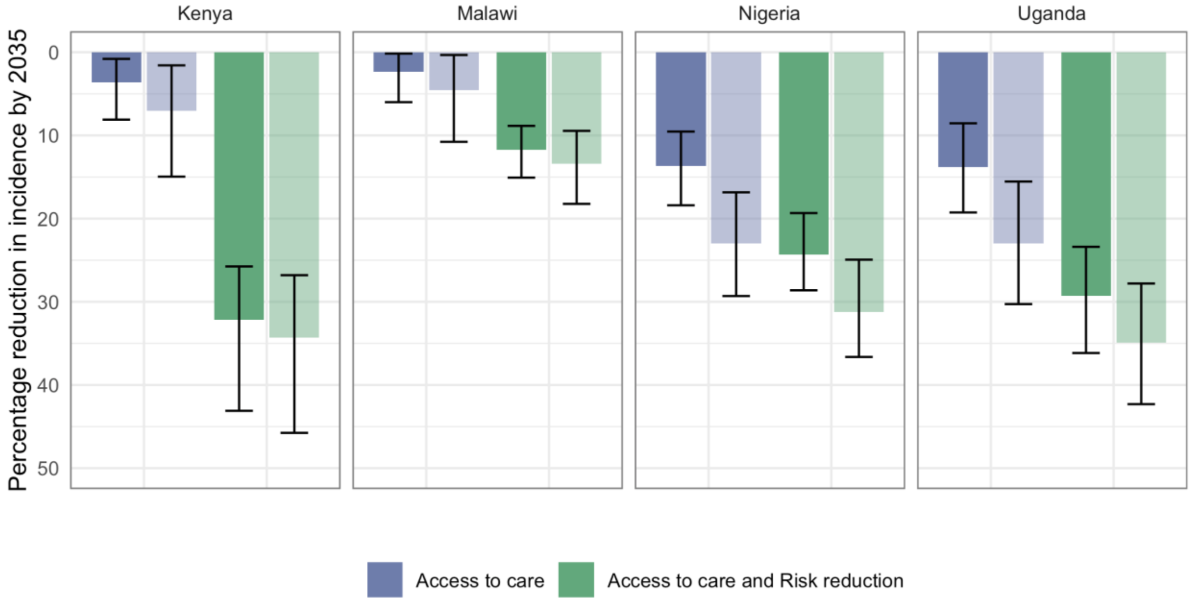

Supplementary Figure 26: The impact of the scenarios where access to care is varied on incidence at the population level, comparing the main analysis where men's access to care is increased to match women's (pale bars), with the supplementary analysis of the difference between men's and women's access to care being halved (bold bars)

7.5 Impact of interventions for varying levels of risk reduction

7.5.1 Incidence

Increasing the levels of risk reduction increases the benefits achieved by strategies reducing risk, but brings less of an improvement to a combined strategy than it does to the single strategy as can be seen in Supplementary Figure 27

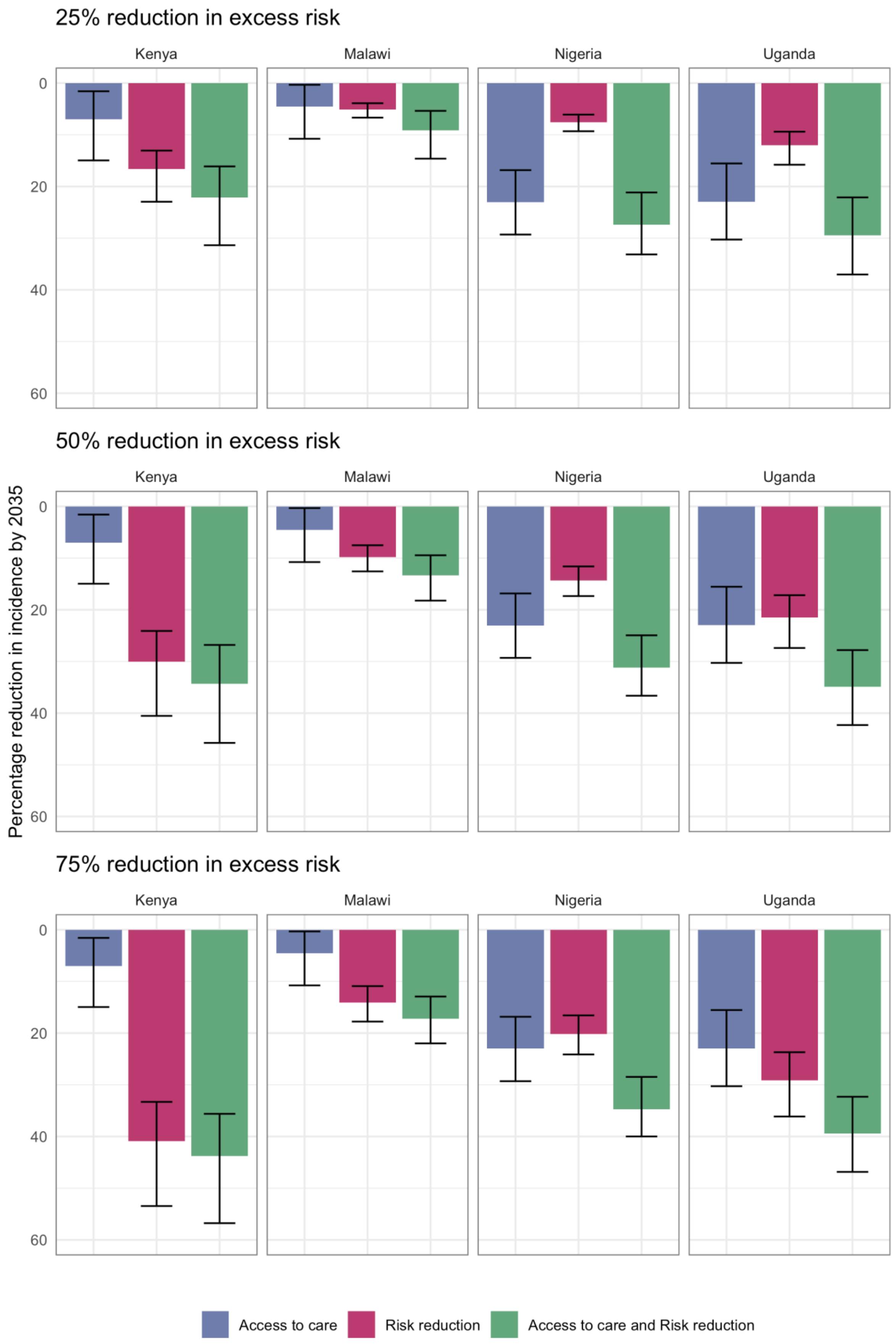

25% reduction in excess risk
50% reduction in excess risk
75% reduction in excess risk
Kenya
Malawi
Nigeria
Uganda
0
20
40
60
Percentage reduction in incidence by 2035
Access to care
Risk reduction
Access to care and Risk reduction

Supplementary Figure 27: The reduction in population-level incidence across the three strategies and across three different risk reduction levels

7.5.2 Notifications

The higher the level of risk reduction, the lower the peak of excess notifications in the combined strategy, and the closer the risk reduction and combined strategies are in change in notifications by 2035, as can be seen in Supplementary Figure 28.

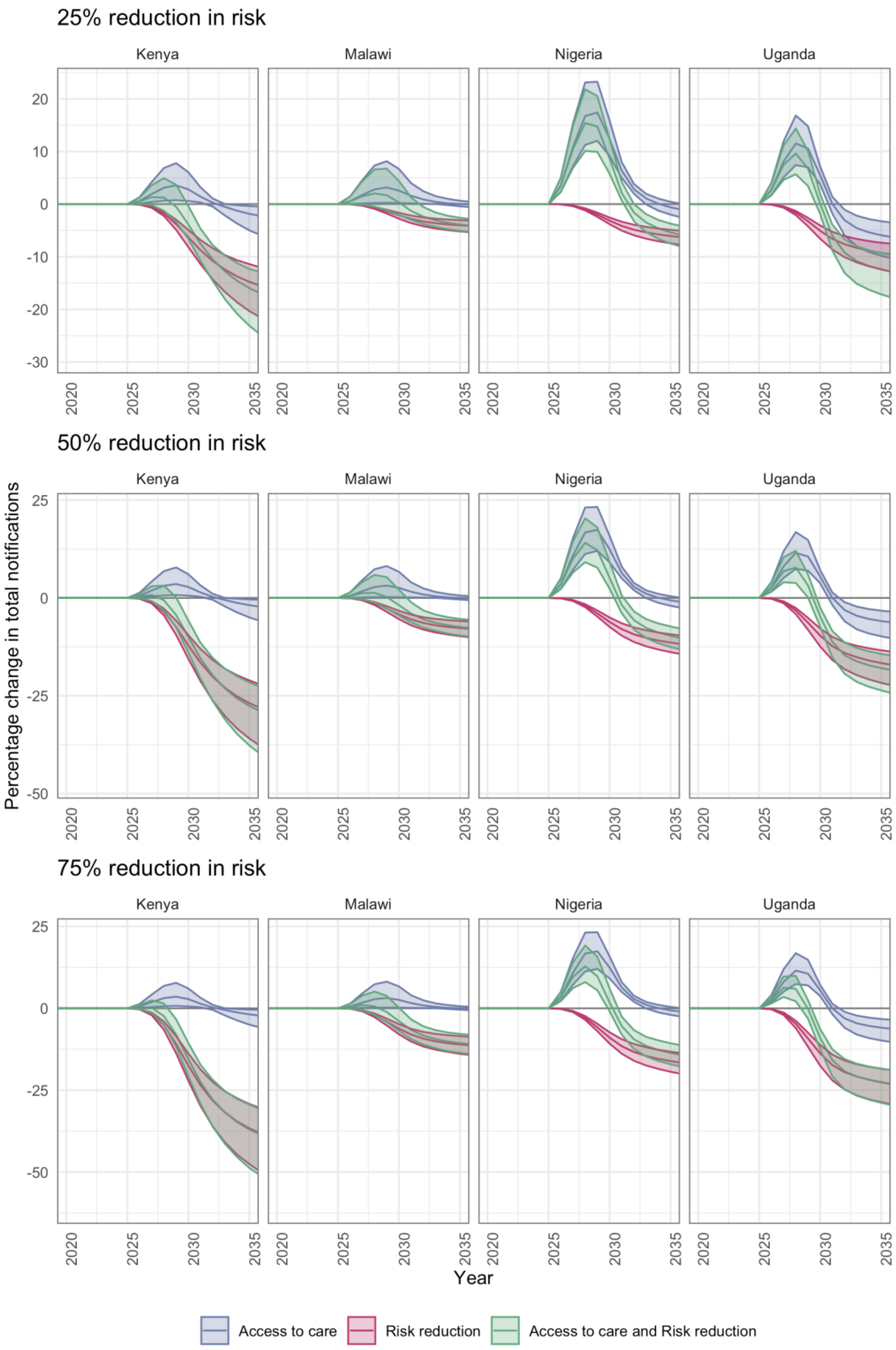

25% reduction in risk
50% reduction in risk
75% reduction in risk
Kenya
Malawi
Nigeria
Uganda
Percentage change in total notifications
Year
Access to care
Risk reduction
Access to care and Risk reduction

Supplementary Figure 28: The change in notifications as risk reduction increases

7.6 Increasing access to care

7.6.1 Incidence overall

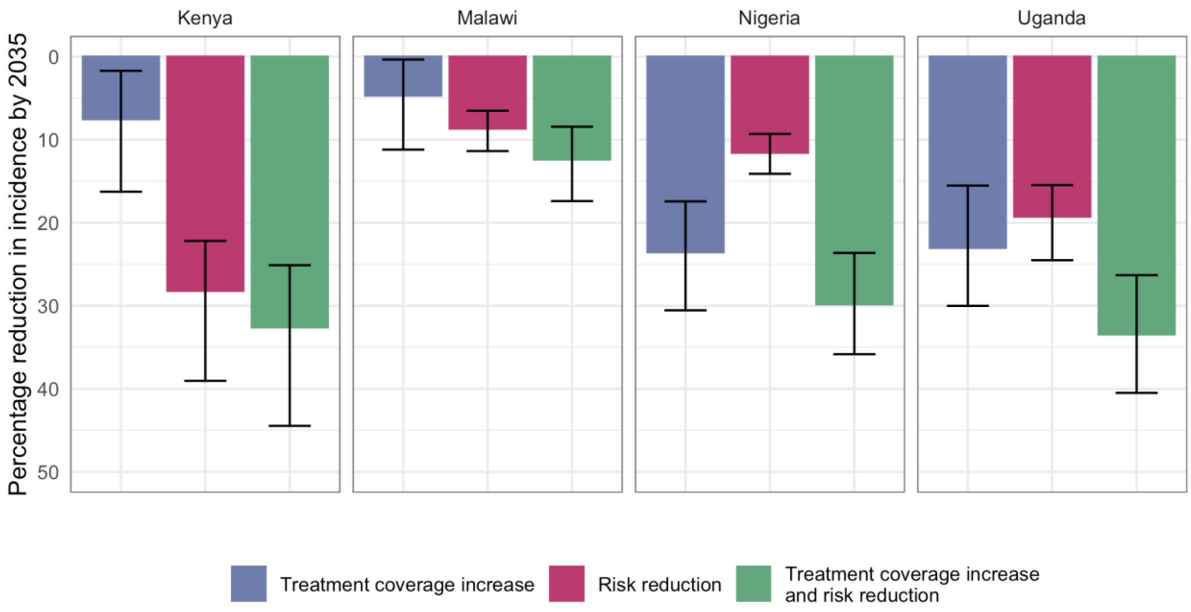

Supplementary Figure 29: Percentage reductions in 2035 TB incidence, relative to a business-as-usual scenario, across the three strategies to address gender disparities in TB - increasing treatment coverage in men, reducing men's excess risk of TB by 50%, and the combination of these two. Coloured bars show the median estimate and error bars show the 95% uncertainty interval.

7.6.2 Incidence Men/Women/Children

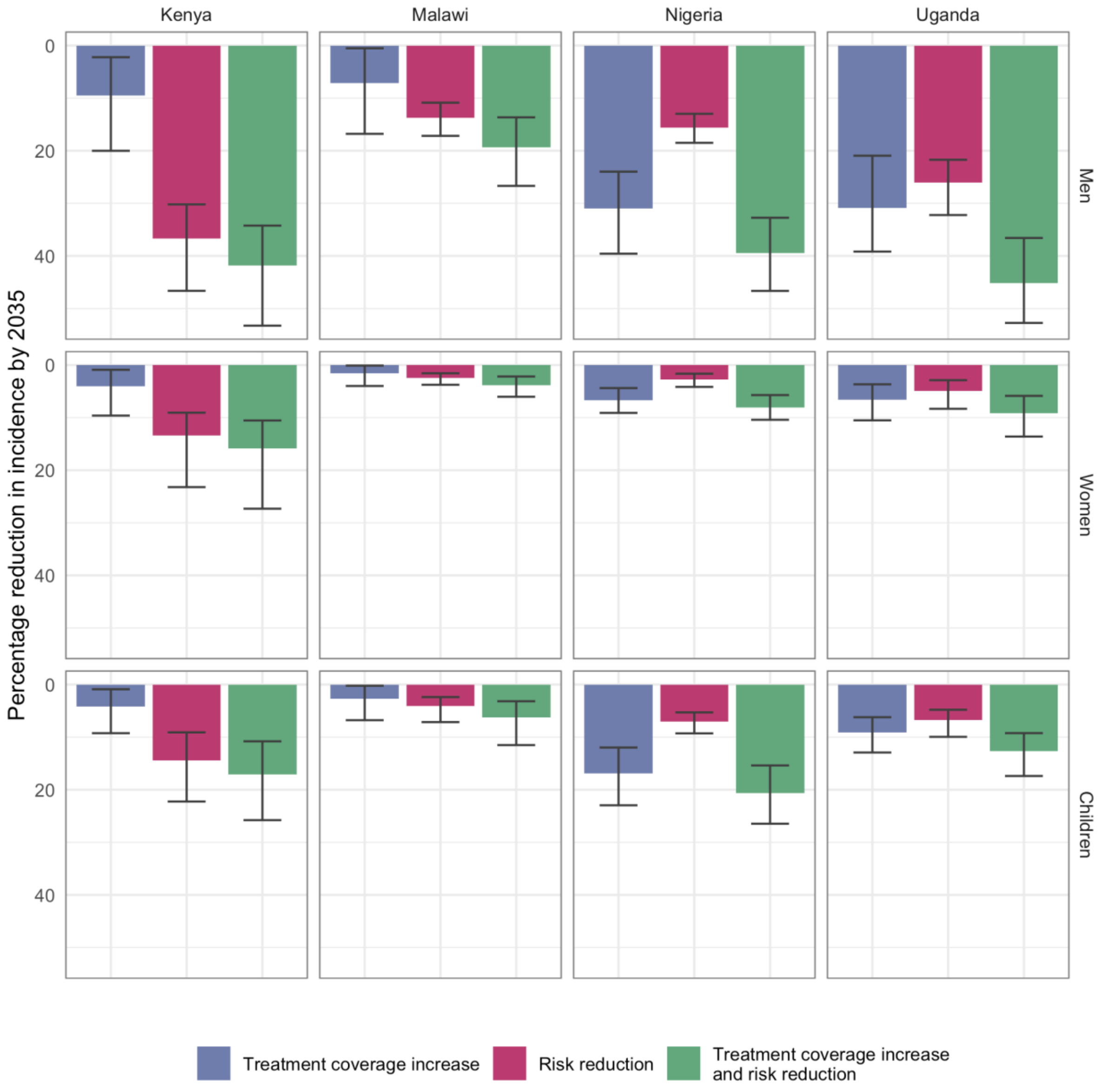

Supplementary Figure 30: Percentage reductions in 2035 TB incidence in men, women, and children, relative to a business-as-usual scenario, across the three strategies to address gender disparities in TB - increasing treatment coverage in men, reducing men's excess risk of TB by 50%, and the combination of these two. Coloured bars show the median estimate and error bars show the 95% uncertainty interval.

7.6.3 Notifications overall

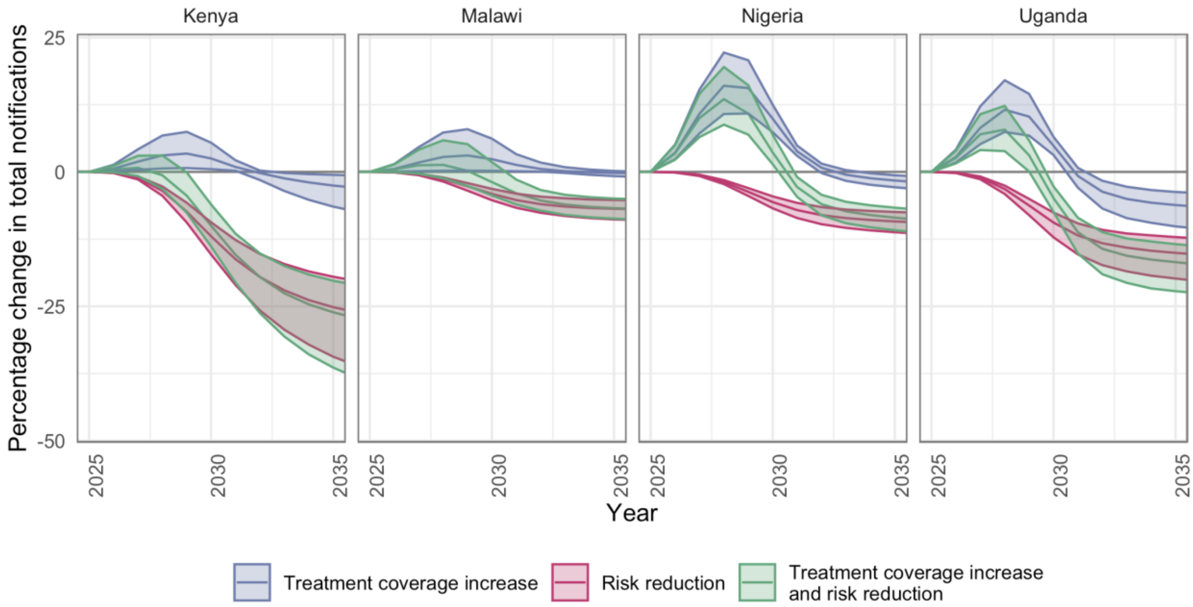

Supplementary Figure 31: The percentage change in notifications from a baseline of no intervention for the five years before, the five years during, and the five years after the implementation of each strategy (increasing treatment coverage in men, reducing men's excess risk of TB by 50%, and the combination of these two). The central lines show the median estimate for the relative percentage reduction and the shaded regions show the 95% uncertainty interval.

8 References

1. Horton KC, White RG, Hoa NB, Nguyen HV, Bakker R, Sumner T, et al. Population benefits of addressing programmatic and social determinants of gender disparities in tuberculosis in viet nam: A modelling study. PLOS Global Public Health. 2022;2: e0000784. doi:10.1371/journal.pgph.0000784

2. Richards AS, Sossen B, Emery JC, Horton KC, Heinsohn T, Frascella B, et al. Quantifying progression and regression across the spectrum of pulmonary tuberculosis: A data synthesis study. The Lancet Global Health. 2023;11: e684–e692. doi:10.1016/S2214-109X(23)00082-7

3. Horton KC, Richards AS, Emery JC, Esmail H, Houben RMGJ. Reevaluating progression and pathways following mycobacterium tuberculosis infection within the spectrum of tuberculosis. Proceedings of the National Academy of Sciences. 2023;120: e2221186120. doi:10.1073/pnas.2221186120

4. World Health Organization. Global tuberculosis report 2024. World Health Organization; 2024. Available: https://www.who.int/teams/global-tuberculosis-programme/tb-reports/global-tuberculosis-report-2024

5. Houben RMGJ, Lalli M, Sumner T, Hamilton M, Pedrazzoli D, Bonsu F, et al. TIME impact a new user-friendly tuberculosis (TB) model to inform TB policy decisions. BMC Medicine. 2016;14: 56. doi:10.1186/s12916-016-0608-4

6. Stover J, McKinnon R, Winfrey B. Spectrum: A model platform for linking maternal and child survival interventions with AIDS, family planning and demographic projections. International Journal of Epidemiology. 2010;39: i7–i10. doi:10.1093/ije/dyq016

7. United Nations. World population prospects - population division - united nations. https://population.un.org/wpp/downloads; 2024.

8. Emery JC, Dodd PJ, Banu S, Frascella B, Garden FL, Horton KC, et al. Estimating the contribution of subclinical tuberculosis disease to transmission: An individual patient data analysis from prevalence surveys. Kana BD, editor. eLife. 2023;12: e82469. doi:10.7554/eLife.82469

9. Dye C, Bassili A, Bierrenbach A, Broekmans J, Chadha V, Glaziou P, et al. Measuring tuberculosis burden, trends, and the impact of control programmes. The Lancet Infectious Diseases. 2008;8: 233–243. doi:10.1016/S1473-3099(07)70291-8

10. Abu-Raddad LJ, Sabatelli L, Achterberg JT, Sugimoto JD, Longini IM, Dye C, et al. Epidemiological benefits of more-effective tuberculosis vaccines, drugs, and diagnostics. Proceedings of the National Academy of Sciences. 2009;106: 13980–13985. doi:10.1073/pnas.0901720106

11. Knight GM, Griffiths UK, Sumner T, Laurence YV, Gheorghe A, Vassall A, et al. Impact and cost-effectiveness of new tuberculosis vaccines in low- and middle-income

countries. Proceedings of the National Academy of Sciences. 2014;111: 15520–15525. doi:10.1073/pnas.1404386111

12. Baguelin M, Flasche S, Camacho A, Demiris N, Miller E, Edmunds WJ. Assessing optimal target populations for influenza vaccination programmes: An evidence synthesis and modelling study. PLOS Medicine. 2013;10: e1001527. doi:10.1371/journal.pmed.1001527

13. Del Fava E, Adema I, Kiti MC, Poletti Pp, Merler S, Nokes DJ, et al. Individual's daily behaviour and intergenerational mixing in different social contexts of kenya | scientific reports. Scientific Reports. 2021;11: 21589. doi:10.1038/s41598-021-00799-1

14. Thindwa D, Jambo KC, Ojal J, MacPherson P, Dennis Phiri M, Pinsent A, et al. Social mixing patterns relevant to infectious diseases spread by close contact in urban Blantyre, Malawi. Epidemics. 2022;40: 100590. doi:10.1016/j.epidem.2022.100590

15. Kleynhans J, Tempia S, McMorrow ML, Gottberg A von, Martinson NA, Kahn K, et al. A cross-sectional study measuring contact patterns using diaries in an urban and a rural community in South Africa, 2018. BMC public health. 2021;21: 1055. doi:10.1186/s12889-021-11136-6

16. Miller PB, Zalwango S, Galiwango R, Kakaire R, Sekandi J, Steinbaum L, et al. Association between tuberculosis in men and social network structure in Kampala, Uganda. BMC infectious diseases. 2021;21: 1023. doi:10.1186/s12879-021-06475-z

17. Potter GE, Wong J, Sugimoto J, Diallo A, Victor JC, Neuzil K, et al. Networks of face-to-face social contacts in Niakhar, Senegal. PloS One. 2019;14: e0220443. doi:10.1371/journal.pone.0220443

18. Marquez C, Chen Y, Atukunda M, Chamie G, Balzer LB, Kironde J, et al. The association between social network characteristics and tuberculosis infection among adults in 9 rural ugandan communities. Clinical Infectious Diseases. 2023;76: e902–e909. doi:10.1093/cid/ciac669

19. Horton KC, MacPherson P, Houben RMGJ, White RG, Corbett EL. Sex differences in tuberculosis burden and notifications in low- and middle-income countries: A systematic review and meta-analysis. PLOS Medicine. 2016;13: e1002119. doi:10.1371/journal.pmed.1002119

20. World Health Organization. Estimate of current tobacco smoking prevalence (%) (age-standardized rate). Available: https://www.who.int/data/gho/data/indicators/indicator-details/GHO/gho-tobacco-control-monitor-current-tobaccouse-tobaccosmoking-cigarrettesmoking-agestd-tobagestdcurr

21. World Health Organization. Alcohol, consumers past 12 months (%), age-standardized. Available: https://www.who.int/data/gho/data/indicators/indicator-details/GHO/alcohol-consumers-past-12-months-(-)

22. NCD-RisC. Prevalence of BMI<18.5 kg/m^2 (underweight) age-standardised. Available: https://ncdrisc.org/data-downloads-adiposity.html

23. World Prison Brief. Prison population total. Available: https://prisonstudies.org/world-prison-brief-data

24. Seddon JA, Chiang SS, Esmail H, Coussens AK. The wonder years: What can primary school children teach us about immunity to mycobacterium tuberculosis? Frontiers in Immunology. 2018;9. doi:10.3389/fimmu.2018.02946

25. Dowdy DW, Chaisson RE. The persistence of tuberculosis in the age of DOTS: Reassessing the effect of case detection. Bulletin of the World Health Organization. 2009;87: 296–304. doi:10.2471/BLT.08.054510

26. Menzies NA, Cohen T, Lin H-H, Murray M, Salomon JA. Population health impact and cost-effectiveness of tuberculosis diagnosis with xpert MTB/RIF: A dynamic simulation and economic evaluation. PLOS Medicine. 2012;9: e1001347. doi:10.1371/journal.pmed.1001347

27. Dye C, Garnett GP, Sleeman K, Williams BG. Prospects for worldwide tuberculosis control under the WHO DOTS strategy. The Lancet. 1998;352: 1886–1891. doi:10.1016/S0140-6736(98)03199-7

28. Eamranond P, Jaramillo E. Tuberculosis in children: Reassessing the need for improved diagnosis in global control strategies. The International Journal of Tuberculosis and Lung Disease. 2001;5: 594–603.

29. World Health Orgaanization. Bacillus calmette-guérin (BCG) vaccination coverage. https://immunizationdata.who.int/pages/coverage/BCG.html?CODE=Global+KEN+NGA+UGA+MWI&YEAR=; 2024.

30. Dutta T. Sex-disaggregated immunization coverage data: Input from key stakeholders. PATH; 2010 Oct.

31. Dodd PJ, Gardiner E, Coghlan R, Seddon JA. Burden of childhood tuberculosis in 22 high-burden countries: A mathematical modelling study. The Lancet Global Health. 2014;2: e453–e459. doi:10.1016/S2214-109X(14)70245-1

32. Sonnenberg P, Glynn JR, Fielding K, Murray J, Godfrey-Faussett P, Shearer S. How soon after infection with HIV does the risk of tuberculosis start to increase? A retrospective cohort study in south african gold miners. The Journal of Infectious Diseases. 2005;191: 150–158. Available: https://www.jstor.org/stable/30077675

33. Lawn SD, Kranzer K, Wood R. Antiretroviral therapy for control of the HIV-associated tuberculosis epidemic in resource-limited settings. Clinics in Chest Medicine. 2009;30: 685–699. doi:10.1016/j.ccm.2009.08.010

34. Ferebee SH. Controlled chemoprophylaxis trials in tuberculosis. A general review. Bibl Tuberc. 1970;26: 28–106. Available: http://www.ncbi.nlm.nih.gov/pubmed/4903501

35. Gilks CF, Godfrey-Faussett P, Batchelor BIF, Ojoo JC, Ojoo SJ, Brindle RJ, et al. Recent transmission of tuberculosis in a cohort of HIV-1-infected female sex workers in

nairobi, kenya. AIDS. 1997;11: 911. Available: https://journals.lww.com/aidsonline/abstract/1997/07000/recent_transmission_of_tuberculosis_in_a_cohort_of.11.aspx

36. Sutherland I, Svandova E, Radhakrishna S. The development of clinical tuberculosis following infection with tubercle bacilli. Tubercle. 1982;63: 255–68. Available: https://doi.org/10.1016/S0041-3879(82)80013-5

37. Cohen T, Lipsitch M, Walensky RP, Murray M. Beneficial and perverse effects of isoniazid preventive therapy for latent tuberculosis infection in HIVtuberculosis coinfected populations. Proceedings of the National Academy of Sciences. 2006;103: 7042–7047. doi:10.1073/pnas.0600349103

38. Hamada Y, Lujan J, Schenkel K, Ford N, Getahun H. Sensitivity and specificity of WHO’s recommended four-symptom screening rule for tuberculosis in people living with HIV: A systematic review and meta-analysis. The Lancet HIV. 2018;5: e515–e523. doi:10.1016/S2352-3018(18)30137-1

39. Stuck L, Klinkenberg E, Abdelgadir Ali N, Basheir Abukaraig EA, Adusi-Poku Y, Alebachew Wagaw Z, et al. Prevalence of subclinical pulmonary tuberculosis in adults in community settings: an individual participant data meta-analysis. The Lancet Infectious Diseases. 2024;24: 726–736. doi:10.1016/S1473-3099(24)00011-2

40. Teo AKJ, MacLean EL-H, Fox GJ. Subclinical tuberculosis: a meta-analysis of prevalence and scoping review of definitions, prevalence and clinical characteristics. European Respiratory Review. 2024;33. doi:10.1183/16000617.0208-2023

41. Dye C, Williams BG. Criteria for the control of drug-resistant tuberculosis. Proceedings of the National Academy of Sciences of the United States of America. 2000;97: 8180–8185. doi:10.1073/pnas.140102797

42. Horton KC, Sumner T, Houben RMGJ, Corbett EL, White RG. A Bayesian approach to understanding gender differences in tuberculosis disease burden. American Journal of Epidemiology. 2018;187: 2431--2438. doi:10.1093/aje/kwy131

43. World Health Organization Global Tuberculosis programme. WHO TB burden estimates. https://www.who.int/teams/global-tuberculosis-programme/data; 2024.

44. Kenya Ministry of Health, National Tuberculosis, Leprosy and Lung Disease Programme. Kenya national strategic plan for 2023/24 to 2027/28. 2023. Available: https://nltp.co.ke/national-strategic-plan-2019-2023-3/

45. Turyahabwe S, Bamuloba M, Mugenyi L, Amanya G, Byaruhanga R, Fry Imoko J, et al. Community tuberculosis screening, testing and care, uganda. Bulletin of the World Health Organization. 2024;102: 400–409. doi:10.2471/BLT.23.290641

46. Corbett EL, Bandason T, Duong T, Dauya E, Makamure B, Churchyard GJ, et al. Comparison of two active case-finding strategies for community-based diagnosis of symptomatic smear-positive tuberculosis and control of infectious tuberculosis in harare, zimbabwe (DETECTB): A cluster-randomised trial. The Lancet. 2010;376: 1244–1253. doi:10.1016/S0140-6736(10)61425-0

47. World Health Organization Global Tuberculosis programme. Treatment success rate: HIV-positive TB cases. https://www.who.int/data/gho/data/indicators/indicator-details/GHO/treatment-success-rate-hiv-positive-tb-cases; 2023.

48. World Health Organization. Treatment outcomes by age group and sex. Available: https://www.who.int/teams/global-programme-on-tuberculosis-and-lung-health/data#csv_files

49. WHO Global Tuberculosis programme. TB: Treatment success. https://www.who.int/data/gho/data/themes/topics/indicator-groups/indicator-group-details/GHO/tb-treatment-success; 2024.

50. Ragonnet R, Flegg JA, Brilleman SL, Tiemersma EW, Melsew YA, McBryde ES, et al. Revisiting the natural history of pulmonary tuberculosis: A bayesian estimation of natural recovery and mortality rates. Clinical Infectious Diseases. 2021;73: e88–e96. doi:10.1093/cid/ciaa602

51. R Core Team. R: A language and environment for statistical computing. Vienna, Austria: R Foundation for Statistical Computing; 2024. Available: https://www.R-project.org/

52. Press WH, Teukolsky SA, Vetterling WT, Flannery BP. Numerical recipes 3rd edition: The art of scientific computing. 3rd ed. Hardcover; Cambridge University Press; 2007.

53. National Tuberculosis L, Lung Disease Program R of K Ministry of Health. Kenya tuberculosis prevalence survey 2016. Nairobi, Kenya; 2018. Available: https://www.nltp.co.ke/survey-reports-2/

54. Ministry of Health NTCP. Technical report: Malawi tuberculosis prevalence survey (2013–2014). 2016.

55. Department of Public Health FR of N. Report of the first national TB prevalence survey 2012, nigeria. 2014.

56. Public Health MUS of. Report on the population-based survey of prevalence of tuberculosis disease in uganda 2014–15. Kampala, Uganda; 2016. Available: https://library.health.go.ug/index.php/communicable-disease/tuberculosis/uganda-national-tuberculosis-prevalence-survey-2014-2015-survey

57. Law I, Floyd K, Group the ATPS. National tuberculosis prevalence surveys in africa, 20082016: An overview of results and lessons learned. Tropical Medicine & International Health. 2020;25: 1308–1327. doi:10.1111/tmi.13485

58. WHO Global Tuberculosis programme. WHO TB incidence estimates disaggregated by age group, sex, and risk factor. https://www.who.int/teams/global-tuberculosis-programme/data; 2024.

59. UN World Population Prospects. Population by select age groups - male. https://population.un.org/wpp/Download/Standard/Population/; 2024.

60. UN World Population Prospects. Population by select age groups - female. https://population.un.org/wpp/Download/Standard/Population/; 2024.

61. World Health Organization. Methods used by WHO to estimate the global burden of TB disease for publication in the global tuberculosis report 2024. 2024 Dec. Available: https://cdn.who.int/media/docs/default-source/global-tuberculosis-report-2024/technical-appendix-gtr-2024_8november2024.pdf?sfvrsn=e9ab9675_2

62. WHO Global Tuberculosis programme. Case notifications. https://www.who.int/teams/global-tuberculosis-programme/data; 2024.

63. Tollefson D, Ngari F, Mwakala M, Gethi D, Kipruto H, Cain K, et al. Under-reporting of sputum smear-positive tuberculosis cases in kenya. The International Journal of Tuberculosis and Lung Disease. 2016;20: 1334–1341. doi:10.5588/ijtld.16.0156

64. Gidado M, Mitchell EMH, Adejumo AO, Levy J, Emperor O, Lawson A, et al. Assessment of TB underreporting by level of reporting system in lagos, nigeria. Public Health Action. 2022;12: 115–120. doi:10.5588/pha.22.0008

65. WHO Global Tuberculosis programme. WHO TB profiles. https://worldhealthorg.shinyapps.io/tb_profiles/; 2024.

66. Iskauskas A, Vernon I, Goldstein M, Scarponi D, McCreesh N, McKinley TJ, et al. Emulation and history matching using the hmer package. Journal of Statistical Software. 2024;109: 1–48. doi:10.18637/jss.v109.i10

67. RStudio Team. RStudio: Integrated development environment for r. Boston, MA: RStudio, PBC.; 2020. Available: http://www.rstudio.com/

68. Ogoamaka C, Bethrand O, Lotanna U, Chidubem O, Sani U, Nkiru N, et al. The TB surge intervention: An optimized approach to TB case-finding in nigeria. Public Health Action. 2023;13: 136–141. doi:10.5588/pha.23.0039

69. Stevenson M, Sergeant E. epiR: Tools for the analysis of epidemiological data. 2025. doi:10.32614/CRAN.package.epiR
